# Long acting progestogens versus combined oral contraceptive pill for preventing recurrence of endometriosis related pain: the PRE-EMPT pragmatic, parallel group, open label, randomised controlled trial

**DOI:** 10.1136/bmj-2023-079006

**Published:** 2024-05-15

**Authors:** Kevin G Cooper, Siladitya Bhattacharya, Jane P Daniels, Andrew W Horne, T Justin Clark, Ertan Saridogan, Versha Cheed, Danielle Pirie, Melyda Melyda, Mark Monahan, Tracy E Roberts, Emma Cox, Clive Stubbs, Lee J Middleton, PRE-EMPT Collaborative Group, Santanu Acharya, Holly Alcock, Hazel Alexander, Rebecca Amos-Hirst, Vicki Atkinson, Thomas Aust, Dolonchampa Basu, Mary Kelly Baxter, Christian Becker, Debbie Callaghan, Fiona Beale, Christian Becker, George Botros, Rebecca Boulton, Jayne Budd, Jodi Carpenter, Tony Chalhoub, Wendy Cheadle, Edel Clare, Sarah Collins, Kathie Cooke, Pamela Corlett, Lisa Cornwall, Hilary Critchley, Sophie Crowder, Elaine Denny, Jean Dent, Helen Dewart, Joanne Donnachie, Ann Doust, Jane Dumville, Sarah Ekladios, Claire Fairhurst, Annika Feilbach, Max Feltham, Leanne Fulcher, Joanne Galliford, Anne Gardner, Tarek Gelbaya, Laura Gennard, Suku George, Cheryl Gibson, Jamie Godsall, Prisca Gondo, Sharon Gowans, Julie Grindey, Janesh Gupta, Holly Hancock, Philip Harris, Marrina Harrison, Helen Harwood, Lesley Hewitt, Pinky Khatri Mary Hodgers, Shahzya Huda, Coralie Huson, Virginia Iqbal, Georgina Jones, Sanjaya Kalkur, Elizabeth Kane, Alison Kimber, Marina Laverdino, Lisa Leighton, Montasser Mahran, Kingshuk Majumder, Nicholas Matthews, Dimitrios Mavrelos, Rachel McCarthy, Shanteela McCooty, Emma Meadows, Bronwyn Middleton, Rupa Modi, Sally Moore, Shoshana Morecroft, Caro Moulds, Eunis Mshengu, Laura Ocansey, Cheryl Padilla, Jennifer Parratt, Minimol Paulose, Deborah Phillips, Una Poultney, Martin Powell, Andrew Prentice, Christina Pritchard, Jyothi Rajeswary, Bruce Ramsay, Kerry Rennie, Samantha Roche, Brice Rodriquez, Fenella Roseman, Fawzia Sanaullah, Jane Scollen, Seema Sen, Manju Singh, Katie Slack, Gillian Smith, Kirandeep Sunner, Amy Sutton, Tracy Taylor, Julie Tebbutt, Premila Thampi, Anne Todd, Konstantinos Tryposkiadis, Louise Underwood, Clare Waters, Christopher Wayne, Lucinda Wilson, Catherine Whittall, Ajith Wijesiriwardana, Adrian Wilcockson, Toni Wilson, Louise Winter, Ahmar Shah Dianne Wood

**Affiliations:** 1 Aberdeen Royal Infirmary, NHS Grampian, Aberdeen, UK; 2 School of Medicine, University of Aberdeen, Aberdeen, UK; 3 Nottingham Clinical Trials Unit, University of Nottingham, Nottingham, UK; 4 MRC Centre for Reproductive Health, University of Edinburgh, Edinburgh, UK; 5 Academic Department of Obstetrics and Gynaecology, Birmingham Women’s and Children’s NHS Foundation Trust, Birmingham, UK; 6 Elizabeth Garrett Anderson Institute for Women's Health, University College London Hospitals NHS Foundation Trust, London, UK; 7 Birmingham Clinical Trials Unit, University of Birmingham, Birmingham, UK; 8 Health Economics Unit, University of Birmingham, Birmingham, UK; 9 Endometriosis UK, London, UK

## Abstract

**Objectives:**

To evaluate the clinical effectiveness of long acting progestogens compared with the combined oral contraceptive pill in preventing recurrence of endometriosis related pain.

**Design:**

The PRE-EMPT (preventing recurrence of endometriosis) pragmatic, parallel group, open label, randomised controlled trial.

**Setting:**

34 UK hospitals.

**Participants:**

405 women of reproductive age undergoing conservative surgery for endometriosis.

**Interventions:**

Participants were randomised in a 1:1 ratio using a secure internet facility to a long acting progestogen (depot medroxyprogesterone acetate or levonorgestrel releasing intrauterine system) or the combined oral contraceptive pill.

**Main outcome measures:**

The primary outcome was pain measured three years after randomisation using the pain domain of the Endometriosis Health Profile 30 (EHP-30) questionnaire. Secondary outcomes (evaluated at six months, one, two, and three years) included the four core and six modular domains of the EHP-30, and treatment failure (further therapeutic surgery or second line medical treatment).

**Results:**

405 women were randomised to receive a long acting progestogen (n=205) or combined oral contraceptive pill (n=200). At three years, there was no difference in pain scores between the groups (adjusted mean difference −0.8, 95% confidence interval −5.7 to 4.2, P=0.76), which had improved by around 40% in both groups compared with preoperative values (an average of 24 and 23 points for long acting progestogen and combined oral contraceptive pill groups, respectively). Most of the other domains of the EHP-30 also showed improvement at all time points compared with preoperative scores, without evidence of any differences between groups. Women randomised to a long acting progestogen underwent fewer surgical procedures or second line treatments compared with those randomised to the combined oral contraceptive pill group (73 *v* 97; hazard ratio 0.67, 95% confidence interval 0.44 to 1.00).

**Conclusions:**

Postoperative prescription of a long acting progestogen or the combined oral contraceptive pill results in similar levels of improvement in endometriosis related pain at three years, with both groups showing around a 40% improvement compared with preoperative levels. While women can be reassured that both options are effective, the reduced risk of repeat surgery for endometriosis and hysterectomy might make long acting reversible progestogens preferable for some.

**Trial registration:**

ISRCTN registry ISRCTN97865475.

## Introduction

Endometriosis is an oestrogen dependent condition that affects up to one in 10 women of reproductive age.^1^ Characterised by the growth of endometrial-like tissue outside the uterus, it can cause severe pelvic pain and infertility that can have a serious impact on quality of life.^2^ ^3^ ^4^ The condition requires a laparoscopy for definitive diagnosis and is frequently treated by excision or ablation of affected tissue at the same time.

Recurrence of endometriosis after surgery is common and poses a major challenge to its successful management. Population based data from Scotland shows that, after initial surgery for endometriosis, 62% of treated women have at least one repeat operation, 45% have two or more, and nearly 25% need surgical removal of their ovaries, often combined with a hysterectomy.^5^

The UK National Institute for Health and Care Excellence and the European Society of Human Reproduction and Embryology recommend the use of hormonal preparations including the combined oral contraceptive pill (COCP) and progestogens to treat endometriosis related pain.^6^ ^7^ It is unclear as to which of these two treatment regimens is better at preventing the recurrence of endometriosis related pain after surgical treatment. Additionally, continuation rates and adherence to treatment might be improved by use of long acting progestogens (LAPs) as there is no need to take drugs on a daily basis.

Our aim was to evaluate whether LAPs or COCP were more effective in preventing the recurrence of pain in women undergoing conservative surgery for endometriosis. The economic results from a parallel cost effectiveness evaluation will be presented in a separate paper.

## Methods

### Trial design

The PRE-EMPT (preventing recurrence of endometriosis) trial was a multicentre, pragmatic, parallel group, open label, superiority randomised controlled trial. In response to clinician and patient feedback that treatment preferences might prevent randomisation to a multiarm trial, the study was designed prospectively to be adaptive, based on feasibility of recruitment; the full methods have been detailed previously.^8^ In brief, during an internal pilot phase, patients could enter the study provided they were willing to be randomised to at least one form of LAP (depot medroxyprogesterone acetate—DMPA, or the levonorgestrel releasing intrauterine system—LNG-IUS) and at least one intervention that was not a LAP (COCP or no treatment). At the end of this pilot phase, a report was provided to a joint trial steering committee and data monitoring committee describing the frequency of randomisation options chosen so that a feasible design including the most commonly chosen options could be agreed for the remainder of the study. A qualitative assessment was also conducted during this time, the results of which fed into any decisions about trial design.^9^ The treatment options described below reflect the revised design that compares LAP as a class of treatments (DMPA or LNG-IUS) versus COCP.

The protocol (supplementary material 1) received clinical trial authorisation (EudraCT 2013-001984-21) from the Medicines and Healthcare products Regulatory Authority and a favourable ethical opinion from the East of Scotland Ethics Committee (14/ES1004). The trial was prospectively registered in the ISRCTN Registry (ISRCTN97865475; https://www.isrctn.com/ISRCTN97865475). A statistical analysis plan was generated for the clinical trial (supplementary material 2), and all participants provided written informed consent. We used the CONSORT (Consolidated Standards of Reporting Trials) checklist when writing this report.^10^

### Participant selection

Recruitment under the definitive design was from 23 November 2015 to 25 March 2019, with 92 participants recruited in the internal pilot phase (from March 2014 to November 2015). Women aged 16-45 years, with symptoms suggestive of endometriosis and scheduled for diagnostic laparoscopy with concurrent surgery for endometriosis (if confirmed), or with a previous laparoscopic diagnosis and scheduled for conservative surgery, were potentially eligible. Exclusion criteria were infertility, immediate plans to conceive, plans for elective endometriosis surgery for deep disease or endometrioma, contraindications to use of hormonal treatment, and suspicion of malignancy at laparoscopy. Previous use of any trial interventions did not preclude participation, while a four week washout period was required before laparoscopy for women using gonadotrophin releasing hormone analogues. An intraoperative diagnosis of treatable peritoneal endometriosis confirmed eligibility.

### Randomisation and interventions

Eligible consenting participants were randomised 1:1 to receive a LAP or COCP. In the LAP group, the options were DMPA, administered at a dose of 150 mg in an aqueous suspension by intramuscular injection every three months, or LNG-IUS that delivers a daily dose of 20 µg of levonorgestrel for five years. Those randomised to COCP were prescribed a formulation containing 30 μg ethinylestradiol and 150 μg levonorgestrel, taken cyclically each month, continuously or in a tricycle regimen. Participants and investigators were not blinded to treatment allocation owing to the substantial differences in formulations and their routes of delivery.

Randomisation occurred intraoperatively or immediately postoperatively using a central internet randomisation service provided by the Birmingham Clinical Trials Unit. Minimisation variables were stage of endometriosis (using the American Society for Reproductive Medicine classification, stage I or II *v* stage III or IV); extent of excision or ablation of endometriosis (complete *v* incomplete, as judged by the surgeon at the time of conservative surgery); age in years (<35 years *v* ≥35 years); randomising centre; intended LAP (if randomised to LAP); reason for selection of LAP (patient preference, clinician advice, or no preference). If the participant had no preference for a particular LAP, the LAP was randomly allocated before LAP versus COCP randomisation using a random blocked list (variable length) incorporated into the computer based algorithm. Patient choice of LAP before randomisation gave them some control over which LAP they would be treated with if randomised to this class of treatment.

### Outcome measures

The primary outcome was the recurrence of symptoms as evaluated by the pain domain of the Endometriosis Health Profile-30 (EHP-30, where 0 is best score and 100 is worst score) three years after randomisation.^11^ ^12^ The EHP-30 is a validated condition specific tool to assess impact on quality of life by endometriosis.

Secondary clinical outcome measures included the remaining EHP-30 core domains (control and powerlessness, emotional wellbeing, social support, self-image) and optional modular domains (work, relationship with children, sexual relationship, feelings about the medical profession, treatment, and infertility). Other secondary outcomes were pain during periods, during intercourse, and at any other time (measured by a visual analogue scale, where 0 was no pain and 100 was worst imaginable pain), a four point ordinal global impression of change in pain, menstrual regularity on a four point ordinal scale, the Fatigue Severity Score,^13^ generic quality of life (measured by EQ-5D-5L),^14^ ^15^ and capabilities (ICECAP-A—ICEpop CAPability measure for Adults).^16^ The results of ICECAP will be presented and discussed in a separate economics paper. Further surgery (laparoscopy to investigate recurrent pain, to treat endometriosis, or hysterectomy) or the use of gonadotrophin releasing hormone analogues were used as a proxy for treatment failure, with a return to preoperative EHP-30 scores also added to these as a further outcome. Change or cessation of randomised treatment, which did not necessitate withdrawal from the trial, were classed as discontinuation. Serious events were classed as those requiring hospital admission or resulting in death or disability, and were categorised as expected or unexpected, and related or unrelated to trial treatment.

Outcomes were collated in a participant completed questionnaire booklet at baseline and then at six months, one, two, and three years. Participants who did not return questionnaires were contacted by telephone to collect the primary outcomes (clinical and economic) and information on further treatment or pregnancy. Other secondary outcomes were not obtained for the telephone completed shortened questionnaire. Data on further surgical procedures and second line medical treatments for endometriosis were obtained directly from participants and also the hospital records of non-responders.

### Sample size

The final sample size calculation reflected the changes to the trial design at the end of the internal pilot phase. The original sample size conservatively assumed the possibility that all treatment options would be taken forward and up to six comparisons would be made; this would require extensive multiplicity adjustments. Because only one main comparison was taken forward, a smaller sample size was needed in the final design. The revised estimate of the standard deviation was taken from pooled baseline data at the end of this pilot phase. These changes were approved by the trial steering committee and data monitoring committee and were made blind to any accruing follow-up data.

To detect an eight point difference on the EHP-30 pain domain with 90% power (P=0.05) and assuming a standard deviation of 22 points required 160 participants per group, 320 in total. To account for a 20% loss to follow-up, this target was inflated to 400. The size of difference targeted (0.36 standard deviation) was considered to be small (0.2 standard deviation) to moderate (0.5 standard deviation). This sample size would also provide 80% power to detect a 10-point difference in the two stratified analyses of LNG-IUS versus COCP, and DMPA versus COCP provided similar numbers were recruited to the DMPA and LNG-IUS groups.

### Statistical analysis

The statistical analysis plan was generated and reviewed by the trial steering committee and data monitoring committee before any analyses were undertaken. Participants were analysed in the treatment group to which they were randomised (intention to treat), irrespective of adherence to the treatment protocol. All participants recruited from 23 October 2015 were included in the final analysis population, along with 92 from the internal pilot phase who were randomised to combinations of treatments that only included LAPs and COCP.

For the primary outcome (EHP-30 pain scores at three years), a mixed effects linear regression model for repeated measures^17^ calculated the adjusted difference between group means, along with 95% confidence intervals (CIs). Parameters for participant, treatment group, time, time by treatment, baseline pain score (as a continuous variable), and the minimisation variables were included; centre was included as a random effect. Secondary outcomes measured on a continuous scale were analysed in a similar manner and other variables using appropriate regression models, dependent on the data type. All estimates of differences between groups were presented with two sided 95% CIs.

Preplanned subgroup analyses on the primary outcome were completed on the minimisation variables, including the selection of LAP (LNG-IUS or DMPA) before randomisation. The effects of these subgroups were examined by adding the subgroup by treatment group interaction parameters to the linear model described above. Sensitivity analysis was performed on the primary outcome to investigate the assumption that missing data were missing at random; this incorporated a delta based multiple imputation approach, which assumes missing data are missing not at random.^18^

Interim analyses of effectiveness and safety endpoints were performed on behalf of the data monitoring committee approximately every year during the period of recruitment. These analyses were conducted using the Haybittle-Peto principle^19^; therefore, no adjustment was made in the final P values to determine significance.

### Patient and public involvement

Input from patients and the public was crucial in shaping the design of the internal pilot and the main trial, and in the choice of the primary and secondary outcomes. Patient and public involvement (PPI) at the design stage of the trial led to the inclusion of the fatigue scale as an outcome measure. As coapplicant, our lead PPI representative provided a patient centred perspective to all discussions and decisions on recruitment, follow-up, and the use of language within documents aimed at participants.

PPI colleagues also influenced our recruitment and follow-up strategies, especially the decision to opt for telephone follow-up for participants after two unsuccessful attempts to contact them by mail. Finally, input from PPI colleagues has been invaluable in interpreting trial results. PPI groups including Endometriosis UK supported the use of several complementary routes of communication to engage with patients from all backgrounds and ensure that the key messages from this trial were available to all those with endometriosis, their families, and all those who care for them.

## Results

**Fig 1:**
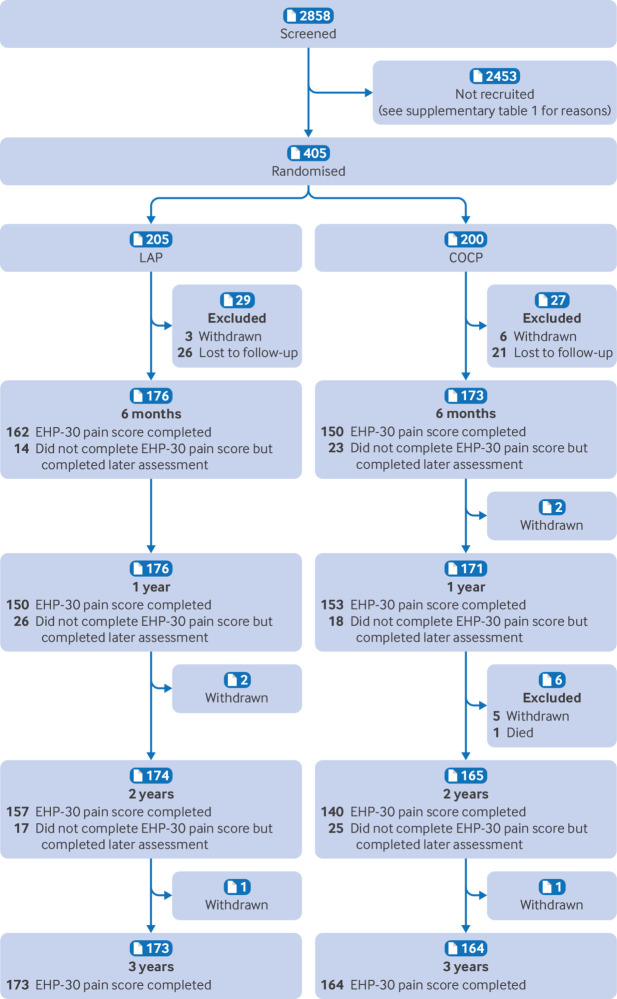
CONSORT (Consolidated Standards of Reporting Trials) trial profile. Completed EHP-30 pain score at any assessment time: LAP, n=195; COCP, n=186. COCP=combined oral contraceptive pill; EHP-30=Endometriosis Health Profile 30; LAP=long acting progestogen

Across 34 UK gynaecology clinics, 2858 women were screened for eligibility and 405 were randomised (fig 1). Supplementary table 1 lists reasons for ineligibility. The follow-up rate for the primary outcome was 337 of 405 (83%) at three years; 381 of 405 (94%) provided an EHP-30 pain score for at least one of the follow-up time points. Final follow-up data were obtained in July 2022.

Participants had a mean age of 29 years (standard deviation 6.6) and most (91%, 369 of 405) described their ethnicity as white (table 1). Endometriosis was graded by the surgeon as stage I or stage II (American Society for Reproductive Medicine classification of minimal or mild) in 79% (319 of 405) of participants and endometrial tissue was deemed to have been completely excised at operation in 91% (369 of 405). The minimisation algorithm ensured balance between groups in terms of age, extent of excision as judged by surgeon, stage of endometriosis, LAP selection, and centre; the groups were also well balanced for the other baseline characteristics.

**Table 1:** Baseline characteristics of participants by randomised group

Characteristics	LAP (n=205)	COCP (n=200)
**Age (years)**		
<35	161 (79)	158 (79)
≥35	44 (21)	42 (21)
Mean (SD)	29.6 (6.7)	29.3 (6.6)
**Body mass index**		
Mean (SD)	27.0 (10.6)	26.3 (5.5)
Missing	12	12
**Ever smoker**		
Yes	38 (26)	39 (26)
No	110 (74)	112 (74)
Missing	57	49
Extent of excision as judged by surgeon*		
Complete	188 (92)	181 (90)
Incomplete	17 (8)	19 (10)
Stage of endometriosis*		
I	88 (43)	82 (41)
II	73 (36)	76 (38)
III	25 (12)	23 (12)
IV	19 (9)	19 (10)
**Self-declared ethnicity**		
White	186 (91)	183 (92)
Mixed	3 (1)	2 (1)
Asian	5 (2)	3 (1)
Black	2 (1)	3 (1)
Other ethnic group	0 (-)	1 (<1)
Not stated	0 (-)	0 (-)
Missing	9	8
**Parity**		
0	103 (50)	120 (60)
1	46 (22)	34 (17)
2	27 (13)	24 (12)
≥3	16 (8)	11 (7)
Missing	13	11
**LAP selection if randomised to LAP (pilot phase recruits n=92)**		
LNG-IUS	17 (35)	16 (36)
DMPA	21 (44)	23 (52)
LNG-IUS or DMPA	10 (21)	5 (12)
LAP selection if randomised to LAP (main phase recruits n=313)*		
LNG-IUS	59 (38)	55 (35)
DMPA	77 (49)	81 (52)
Randomly allocated	21 (13)	20 (13)
Mode of LAP selection† (main phase recruits n=313)*		
Patient’s preference	126 (80)	128 (82)
Clinician advice	10 (6)	8 (5)
Neither	21 (13)	20 (13)
**Previous treatment (more than one modality possible)‡**		
LNG-IUS	27 (7)	21 (5)
DMPA	31 (8)	28 (7)
COCP	48 (12)	44 (11)

Data are number (%) of participants unless stated otherwise.

COCP=combined oral contraceptive pill; DMPA=depot medroxyprogesterone acetate; LAP=long acting progestogen; LNG-IUS=levonorgestrel releasing intrauterine system; SD=standard deviation.

* Minimisation variable.

† Selection before randomisation.

‡ Figures might total more than number randomised because treatments are not mutually exclusive.

Of the 205 women randomised to LAP, a few more were offered treatment with DMPA compared with LNG-IUS (114 (56%) *v* 91 (44%)). Approximately four-fifths (81%, 254 of 313; table 1) of these treatment options were driven by patient preference. Approximately 65% of participants allocated a LAP were still using a LAP at one year, reducing to 37% by three years. The equivalent figures in the COCP group were lower at 53% and 25%, respectively (supplementary figure 1, panel A). Switching from one LAP treatment to another (ie, from LNG-IUS to DMPA or vice versa) or supplementation of a (related) non-trial drug was also a relatively common occurrence. Adherence to the initially allocated treatment (without any treatment change at all) occurred in 56% and 48% of participants at one year and 26% and 24% at three years in the LAP and COCP groups, respectively (supplementary figure 1, panel B; data are provided for LNG-IUS and DMPA separately in supplementary figure 2). Supplementary tables 2 and 3 summarise reasons for non-adherence.

### Primary outcome measure

**Fig 2:**
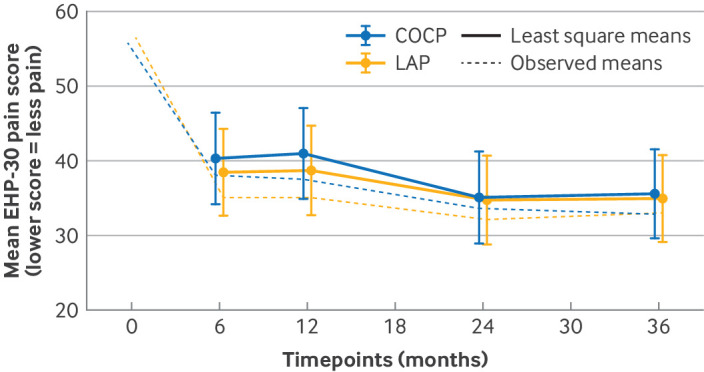
EHP-30 pain scores over all time points. COCP=combined oral contraceptive pill; EHP-30=Endometriosis Health Profile 30; LAP=long acting progestogen

Three years after randomisation, no evidence was found of a statistically significant difference in pain scores between groups (adjusted mean difference −0.8, 95% CI −5.7 to 4.2; P=0.76), with both groups showing a similar reduction of around 40% (on average, 24 points for LAP group and 23 points for COCP group) compared with preoperative values (table 2). On average, both groups maintained improved pain scores at all follow-up intervals compared with their preoperative scores (supplementary table 4; fig 2). We did not find any differential effect in any of the prespecified subgroups relating to the primary outcome (supplementary table 5). Sensitivity analysis conducted to investigate missing data assumptions did not alter the initial interpretation (supplementary table 6).

**Table 2:** Results of primary and secondary outcomes at three years

Outcome	Long acting progestogen*	Combined oral contraceptive pill*	Adjusted mean difference (95% CI)
**Primary outcome—EHP-30 pain score**†			
Baseline	56.6 (17.3), 197	55.8 (19.9), 192	—
3 years	32.9 (25.0), 173	32.9 (27.6), 164	−0.8 (−5.7 to 4.2)‡
**Secondary outcome—EHP-30 core domains**†			
Control and powerlessness			
Baseline	69.1 (19.7), 198	66.6 (23.4), 193	—
3 years	40.9 (28.5), 103	45.4 (34.2), 99	−2.4 (−10.0 to 5.2)
Social support			
Baseline	56.8 (23.5), 198	56.5 (26.5), 193	—
3 years	40.7 (31.5), 102	48.4 (36.1), 99	−5.1 (−12.6 to 2.5)
Emotional wellbeing			
Baseline	53.0 (20.3), 198	52.4 (23.2), 193	—
3 years	35.6 (26.6), 103	38.6 (31.1), 99	−1.8 (−8.2 to 4.7)
Core domain: self-image			
Baseline	54.3 (28.4), 198	52.6 (29.0), 194	—
3 years	43.7 (34.4), 103	48.1 (36.7), 99	−1.7 (−9.6 to 6.2)
**Secondary outcome—EHP-30 modular domains**†			
Work life			
Baseline	51.2 (25.9), 165	50.2 (28.0), 168	—
3 years	23.5 (25.4), 94	23.2 (27.4), 79	−0.7 (−8.5 to 7.1)
Relationship with children			
Baseline	40.5 (29.9), 107	33.5 (26.6), 87	—
3 years	19.9 (25.8), 47	19.3 (28.7), 42	−4.2 (−14.7 to 6.4)
Sexual relationship			
Baseline	68.4 (26.0), 173	69.6 (24.3), 169	—
3 years	53.4 (31.7), 87	55.9 (32.5), 87	−0.0 (−8.4 to 8.4)
Feelings about medical profession			
Baseline	36.0 (29.0), 169	31.2 (27.9), 162	—
3 years	41.3 (33.0), 53	43.1 (34.1), 58	−3.4 (−15.0 to 8.1)
Feelings about treatment			
Baseline	48.3 (26.1), 121	46.4 (27.5), 115	—
3 years	40.4 (27.2), 65	39.1 (32.7), 67	2.5 (−8.7 to 13.7)
Feelings about infertility			
Baseline	49.9 (32.5), 110	48.5 (33.7), 110	—
3 years	55.9 (31.2), 35	44.9 (36.3), 45	4.3 (−9.8 to 18.4)
**Secondary outcome—pelvic pain using visual analogue scale§**			
Pain during periods			
Baseline	7.8 (1.4), 158	7.9 (1.5), 152	—
3 years	7.0 (1.7), 44	7.0 (2.1), 53	−0.4 (−1.2 to 0.4)
Pain during intercourse			
Baseline	6.4 (2.4), 150	6.4 (2.6), 159	—
3 years	5.4 (3.0), 63	5.6 (2.8), 74	0.2 (−0.6 to 1.1)
Pain at any other time			
Baseline	6.4 (2.0), 180	5.8 (2.1), 175	—
3 years	5.3 (2.3), 81	5.4 (2.5), 78	0.3 (−0.4 to 1.0)
Secondary outcome—Fatigue Severity Score¶			
Baseline	43.6 (14.1), 197	42.3 (13.4), 191	—
3 years	43.0 (15.1), 102	42.0 (17.1), 98	1.5 (−2.0 to 5.1)
Secondary outcome—EQ-5D-5L**			
Baseline	0.63 (0.24), 198	0.63 (0.24), 190	—
3 years	0.69 (0.27), 176	0.69 (0.29), 167	−0.01 (−0.06 to 0.04)
**Secondary outcome—Likert scale changes in pelvic pain,**††** number (%)**			
Got much better	5 (6)	5 (6)	0.83 (0.44 to 1.57)‡‡
Got a little better	4 (5)	6 (8)	
Not changed much	42 (50)	38 (48)	
Got worse	33 (39)	30 (38)	
Total number	84	79	
**Secondary outcome—still experiencing periods (menstrual status), number (%)**			
Yes	51 (51)	62 (63)	
No	50 (50)	36 (37)	
Total number	101	98	
**Secondary outcome—menstrual cycle regularity, number (%)**			
Regular	12 (25)	13 (21)	1.43 (0.63 to 3.24)‡‡,§§
Fairly regular	15 (31)	16 (26)	
Irregular	15 (31)	24 (39)	
Bleeding on and off	6 (13)	8 (13)	
Total number	48	61	

CI=confidence interval; EHP-30=Endometriosis Health Profile 30; LAP=long acting progestogen; SD=standard deviation.

* Data are mean (standard deviation), number.

† EHP-30 pain domain; score ranges from 0 (not affected) to 100 (worst affected).

‡ Differences <0 favour LAP.

§ Visual analogue scale scores range from 0 (best outcome) to 10 (worse outcome); scores <0 favour LAP.

¶ Fatigue Severity Scale scores range from 9 to 63 (higher score=greater fatigue severity); scores <0 favour LAP.

** EQ-5D-5L scores range from −0.59 (worse outcome) to 1.00 (best outcome); scores >0 favour LAP.

†† Odds ratio from proportional odds model shown; estimates <1 favour LAP.

‡‡ Adjusted odds ratio (95% confidence interval).

§§ Centre removed from model owing to lack of convergence.

### Secondary outcomes

Most of the domains of the EHP-30 were improved in both groups at all time points compared with preoperative scores, but there was no consistent evidence of any difference between groups (table 2 for results at three years; supplementary table 7 other time points). Pain scores as measured by a visual analogue scale marginally improved at all time points compared with preoperative scores, and when pain was measured by a Likert scale, responses appeared consistent throughout, with most women reporting that their pelvic pain had not changed much or had become worse over the past month. There was no evidence of consistent differences between the groups (supplementary table 8).

The Fatigue Severity Scale results (supplementary table 9) were similar to baseline scores throughout in both groups, while generic quality of life scores showed marginal improvement compared with preoperative values (supplementary table 10). The numbers of participants reporting menstrual periods remained relatively consistent throughout and were lower in the LAP group than the COCP group (54% (87/161) at six months, 51% (51/101) at three years *v* 76% (116/152) at six months, 63% (62/98) at three years, respectively; supplementary table 11); these periods appeared to be less regular in the LAP group during the early stages of follow-up (supplementary table 12), but were comparable at three years (table 2). The number of recorded pregnancies was 17 in the LAP group and 24 in the COCP group (supplementary table 13).

**Fig 3:**
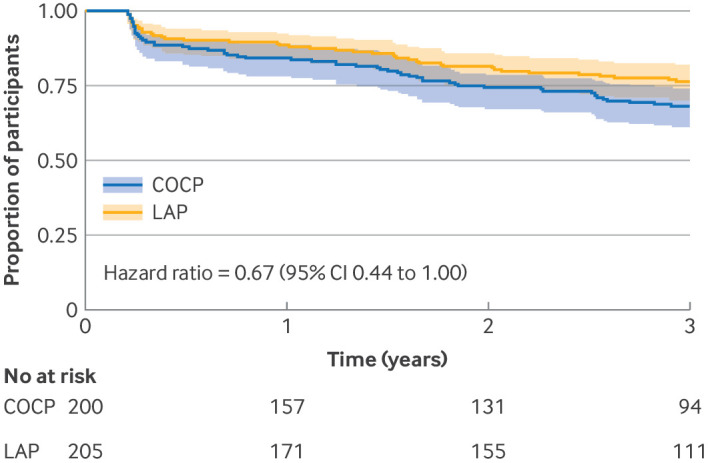
Kaplan-Meier plot of time without further therapeutic surgery or second line treatment. CI=confidence interval; COCP=combined oral contraceptive pill; LAP=long acting progestogen

Fewer women required additional treatment in the LAP group compared with the COCP group (73 *v* 97 events, occurring in 50 *v* 61 women because of several repeat interventions in some participants; supplementary table 14), translating to a 33% reduction in time to treatment failure (fig 3; hazard ratio 0.67, 95% CI 0.44 to 1.00). Inclusion of return to prerandomisation EHP-30 pain score into the definition of treatment failure showed 11% fewer failures in the LAP group than in the COCP group (supplementary figure 3, hazard ratio 0.89, 95% CI 0.66 to 1.19).

There were 21 serious adverse events in 14 women in the LAP group and 17 events in 15 women in the COCP group (P=0.79), none directly related to the trial treatment. Seven reports (four in LAP group, three in COCP group) were linked to planned pregnancy and birth, eight (four in each group) associated with recurrent pain, and seven (four LAP, three COCP) were associated with the index endometriosis surgery. The remainder were incidental hospital admissions.

## Discussion

### Statement of principal findings

A strategy of prescribing LAP or COCP after surgery for endometriosis resulted in similar levels of pain at three years, with both groups reporting an improvement of almost 40% from pretreatment levels on average. Choice of a particular LAP (LNG-IUS or DMPA) before randomisation did not alter these findings. Use of LAPs reduced the risk of second line medical treatments and further surgery.

### Strengths and weaknesses of the study

This large randomised trial evaluated hormonal treatments for endometriosis related pain with a long follow-up at three years, and also included an economic evaluation of postoperative use of LAP or COCP (the results will be reported in a separate paper). In addition to strict randomisation and flexibility in the interventions, the major strengths of this trial include its focus on patient centred outcomes, and the availability of primary outcome data on more than 80% (337/405) of participants. The pragmatic nature of the trial is more likely to enhance the generalisability of our findings, although the predominance of white women in the recruited sample limits our confidence about extrapolating the results to women from other ethnic groups.

The three year follow-up period and the pragmatic design meant that relatively few women continued on their initially allocated drug, changing or stopping their treatments depending on their circumstances, including changes in reproductive plans. The assumed improved continuation rate over COCP (25%) was marginal for DMPA (30%) but was evident for LNG-IUS (46%), which might mean the delivery method was better tolerated, but could equally represent the need to have the LNG-IUS removed at a medical facility. While these low adherence rates will have decreased the ability of the trial to detect a meaningful difference in efficacy between the two interventions, they do not necessarily detract from our ability to address the main aim of this pragmatic trial, which was to compare a policy of prescribing COCP or LAP after surgery for endometriosis over a three year time period.

PRE-EMPT provides data on only two of the three symptom outcomes in the core outcome set for endometriosis, which were published after the trial started.^20^ The precision of comparison of secondary outcomes was decreased by missing data owing to the prioritisation of methods designed to capture the primary outcome.

Treating the two LAP preparations as a single intervention assumes a comparable mechanism of action and potential impact on symptoms. Both treatments cause progestogenic effects, but there might be other modes of action: LNG-IUS acts locally in the uterus while DMPA is systemic and results in ovarian suppression. Balanced subgroup analysis did not show any differential effect on primary outcome measures. The current design also limits power for meaningful comparisons between LNG-IUS or DMPA individually with COCP. While these factors make it difficult to comment on the efficacy of LAPs and COCP, the results of this trial allow a clear understanding of the medium term value of prescribing either class of drug after endometriosis surgery.

### Strengths and weaknesses in relation to other studies

The prolonged duration of this trial, which started recruitment in 2014, means that newer hormonal treatment options for endometriosis have become available, including the fourth generation synthetic oral progestogen dienogest^21^ and oral gonadotrophin releasing hormone antagonists^22^ containing add-back hormone replacement. Importantly, however, LAPs and COCP are commonly used hormonal contraceptives worldwide; they are cheap, easily accessed, and have a well known side effect and safety profile. Although the follow-up period is the longest of any comparable trial,^23^ the evidence provided by this trial is only relevant for the three years after surgery in a condition that can persist until menopause and often requires several episodes of further treatment.^5^

The absence of a no treatment option prevented exploration of the impact of surgery alone, although a systematic review involving 17 studies of various hormonal treatments for different endometriosis subtypes showed a decreased risk of recurrence associated with their use.^24^ Our trial also assumes an inherent benefit from surgery, which has not been conclusively shown.^25^ However, the first six months after surgery does reveal the biggest reduction in self-reported pain scores. An ongoing trial, ESPRIT 2 (https://www.ed.ac.uk/centre-reproductive-health/esprit2), aims to assess the short term impact of destruction of superficial endometriosis lesions compared with laparoscopy alone, but as choice of postoperative hormones will be determined by participants, LAPs and COCP will not be compared. Although recruitment was completed before the covid-19 pandemic, the restrictions on elective surgeries in 2020 and the length of subsequent surgical waiting lists might have reduced the number of repeat procedures.

### Meaning of the study

The results of this trial show that prescribing a LAP or COCP is equally effective in reducing pain three years after endometriosis surgery, and reinforce current guidance recommending routine postsurgical hormonal treatment in this context. Women undergoing laparoscopic surgery can be informed that either class of hormonal drug reduces pain over a three year period and that LAPs could lower the risk of further surgery. Healthcare providers can note that prescribing LAPs reduces the need for further second line treatments.

### Unanswered questions and future research

Other hormonal drugs, including dienogest and combination gonadotrophin releasing hormone antagonists with add-back hormone preparations, should be compared against LAPs and the COCP to determine relative effectiveness in preventing recurrence of pain, and their costs. The identification of non-invasive methods to diagnose endometriosis (radiological or by reliable blood and urinary biomarkers) to avoid the need for initial and repeat laparoscopy would be hugely beneficial. Therefore, future research should focus on early, non-invasive diagnosis and effective treatment of endometriosis to ensure long term alleviation of pain and improved quality of life.

**What is already known on this topic**

Laparoscopic excisional or ablative surgery for endometriosis has been shown to improve symptoms of pain, but postoperative recurrence is common
The combined oral contraceptive pill (COCP) and progestogens are widely used to treat endometriosis related pain; long acting progestogens (LAPs) have the advantage of requiring less frequent administration
Uncertainty exists about which hormonal option (COCP or LAPs) is better for preventing recurrence of pain after surgery to remove endometriosis

**What this study adds**

Prescribing the COCP or LAPs after surgery for endometriosis resulted in a 40% reduction in pain scores in both treatment groups at three years
Women in the LAP treatment group were less likely to need second line medical treatments and further surgery

## Data Availability

All data requests should be submitted to bctudatashare@contacts.bham.ac.uk for consideration. Access to anonymised data might be granted after review.

## Web extra

Extra material supplied by authors

Web appendix: Supplementary material 1—protocol

Web appendix: Supplementary material 2—statistical analysis plan

Web appendix: Supplementary material 3—acknowledgments

Web appendix: Supplementary material 4—tables and figures

## Notes

Contributors: KGC, SB, JPD, TER, and LJM conceived the study, acquired funding, defined the methodology, supervised the research and drafted the manuscript. TJC, AWH, and ES also contributed to the acquisition of funding. LJM and VC conducted the analyses and created the data visualisations. All authors were involved in the investigation and reviewed and edited the manuscript. The lead author attests that all listed authors meet authorship criteria and that no others meeting the criteria have been omitted. KGC is the guarantor.

Transparency: The lead author (the manuscript’s guarantor) affirms that the manuscript is an honest, accurate, and transparent account of the study being reported; that no important aspects of the study have been omitted; and that any discrepancies from the study as originally planned (and, if relevant, registered) have been explained.

Dissemination to participants and related patient and public communities: The authors will work closely with the national patient organisation, Endometriosis UK (the CEO, Emma Cox, is a coauthor), the University of Birmingham, and BMJ press officers to produce a press release to accompany the publication of the article. Together with Endometriosis UK, the authors will prepare a lay version of the article for publication on their website and sharing through their social media channels (X (formerly Twitter), Instagram, Facebook, LinkedIn, and TikTok). The authors will work with the World Endometriosis Society to produce a summary of the results (linked to the article) for their website (the president-elect, Andrew Horne, is a coauthor), and for distribution to Endometriosis UK’s network of volunteers, members, and supporters. A similar article will be produced for the British Society of Gynaecological Endoscopy website (past presidents, Justin Clark and Ertan Saridogan, are coauthors). The authors will submit an abstract of the data for presentation at the World Conference on Endometriosis (WCE2027). In addition, the authors will engage with the government’s women’s health teams in four nations in the UK, along with other relevant bodies including the Scottish Government’s Women’s Health Group, the Scottish Government Cross Party Group on Women’s Health, and the UK Government’s All Party Parliamentary Group for Endometriosis (Endometriosis UK provide the secretariat) to ensure that the results are used to influence endometriosis management policy. Endometriosis UK will also work with partners and grassroots organisations to extend reach to diverse groups, including from different ethnic backgrounds A full trial report with clinical and economic results will also be published in Health Technology Assessment (ISSN: 2046-4924).

Provenance and peer review: Not commissioned, externally peer reviewed.
