# Supplementary material for: Long acting progestogens versus combined oral contraceptive pill for preventing recurrence of endometriosis related pain: the PRE-EMPT pragmatic, parallel group, open label, randomised controlled trial
Source: BMJ. 2024 May 15;385:e079006. doi: 10.1136/bmj-2023-079006 (PMC11094611; doi:10.1136/bmj-2023-079006)
Supplement: Supplementary file 1 — Web appendix: Supplementary material 1—protocol [file cook079006.ww1.pdf]

The PRE-EMPT Trial: Preventing REcurrence of Endometriosis by Means of long acting Progestogen Therapy

PROTOCOL

Plain English Summary

Endometriosis is a common condition where cells similar to those within the lining of the womb are found in abnormal locations elsewhere in the body, commonly within the pelvis. Like the lining of the womb itself, these cells respond to oestrogen produced by the ovaries and go through a phase of growth followed by breakdown and bleeding. This internal bleeding within the pelvis causes inflammation, the formation of adhesions and is associated with pain. Endometriosis occurs in 6-10% of women of reproductive age. The condition is painful and can have a serious impact on their lives. Many will need surgery to remove areas of endometriosis in order to relieve pain. However, symptoms of endometriosis tend to return and women need to go through repeated surgery including removal of their womb and ovaries. Endometriosis costs the UK >£2.8 billion/year in loss of productivity mainly due to associated pelvic pain symptoms.

Drugs which reduce levels of oestrogen can prevent the re-growth of endometriosis. Previous research has suggested that medicines containing other hormones such as progestogens can reduce the chances of symptoms returning. However, these studies were done with small numbers of participants and were unable to provide definitive results. This is a protocol for a large randomised controlled clinical trial in which women undergoing surgery for endometriosis will be randomly allocated to take long acting progestogens (either as three monthly injections or as a contraceptive coil, or long term treatment with the oral contraceptive pill or no treatment). The trial will provide information on which treatment is the most effective in terms of symptom relief, side-effects, acceptability and costs. This information will be vital in terms of future clinical decision making in an area of uncertainty.

This study is funded by the National Institute for Health Research (NIHR) Health Technology Assessment Programme (project reference 11/114/01). The views expressed are those of the author(s) and not necessarily those of the NIHR or the Department of Health and Social Care.

PROTOCOL SUMMARY

DESIGN:	**A randomised, pragmatic multicentre trial with integrated economic evaluation.**
SETTING:	Up to 40 NHS hospitals within the United Kingdom
TARGET POPULATION:	Women of reproductive age, who are undergoing a laparoscopy to investigate whether their pelvic pain is due to endometriosis. Exclusion criteria: current infertility, immediate plans to conceive.
HEALTH TECHNOLOGIES ASSESSED:	The main comparison is long-acting reversible contraception (LARC) versus combined oral contraceptive pill (COCP). Participants can have a pre-randomisation choice of LARC (or alternatively one will be randomly allocated): i) levonorgestrel-releasing intra-uterine system (LNG-IUS) (fitted by gynaecologist) or ii) 3 monthly depot medroxyprogesterone acetate (DMPA) injection (administered by the patient's gynaecologist or general practitioner); sub-comparisons will be stratified by this choice.
OUTCOMES:	The primary outcome is the recurrence of symptoms as evaluated by the pain domain of the Endometriosis Health Profile – 30 (EHP-30) questionnaire at 36 months post-randomisation. The EHP-30 is a validated, responsive health related quality of life measure for endometriosis. It will also be assessed prior to randomisation and at 6, 12 and 24 months.<br>Secondary outcomes:<br>• All other symptom and quality of life (QoL) domains of the EHP-30<br>• Non-menstrual pelvic pain and dysmenorrhea measured by 0-100 visual analogue (VAS) pain scale<br>• Fatigue, as measured by Fatigue Severity Score<br>• Menstrual regularity<br>• Generic QoL (EQ-5D) and capabilities, as measure of wellbeing (ICE-CAP)<br>• Further diagnostic and therapeutic surgery for endometriosis (as a proxy for recurrence)<br>• Discontinuation rates of randomised treatment, with reasons for change, serious adverse events<br>• Cost per quality adjusted life year (QALY) and cost per change in symptom score.<br>• An increased knowledge of issues identified as important by the participants regarding their treatment and its impact on their lives
ANALYSIS:	The main comparison will be LARC v COCP, with sub-comparisons of the groups where the intention is to treat with either LNG-IUS or DMPA if randomised to LARC. The primary outcome will be analysed using a linear regression model including a variable for each treatment group and including baseline score and the minimisation factors as covariates. Effect sizes will be presented as point estimates and 95% confidence intervals. Standard statistical methods will be used for other outcomes. All analysis will be by intention to treat.
SAMPLE SIZE:	The study will have 90% power (p=0.05) to detect an 8 point difference in the main comparison assuming the standard deviation of the EHP-30 pain domain is 22 points. This will require 160 women per group, 320 in total. To account for 20% loss to follow-up this target has been inflated to 400 women in total.

PRE-EMPT Trial Management Group

Chief Investigator

Professor Kevin Cooper
Consultant Obstetrician & Gynaecologist
NHS Grampian.
Aberdeen Royal Infirmary,
Foresterhill,
ABERDEEN,
AB25 2ZN
Email: Kevin.cooper@nhs.scot

Trial Coordination and Statistics

Professor Jane Daniels
Email: jane.daniels@nottingham.ac.uk

Clive Stubbs
Email: pre-empt@trials.bham.ac.uk

Jamie Godsall
Email: pre-empt@trials.bham.ac.uk

Lee Middleton
Email: l.j.middleton@bham.ac.uk

Alicia Gill
Email: A.Gill.2@bham.ac.uk
Birmingham Clinical Trials Unit
University of Birmingham

Health Economics

Professor Tracy Roberts
Email: t.e.roberts@bham.ac.uk

Clinical Lead Investigators

Dr Andrew Prentice
University of Cambridge
Email: ap.128@outlook.com
Professor Andrew Horne
University of Edinburgh
Email: andrew.horne@ed.ac.uk

Professor Hilary OD Critchley
University of Edinburgh
Email: hilary.critchley@ed.ac.uk

Professor Janesh K. Gupta
Birmingham Women's Hospital
Email: j.k.gupta@bham.ac.uk

Professor T Justin Clark
Birmingham Women's Hospital
Email: Justin.Clark@bwnft.nhs.uk

Professor Christian Becker
University of Oxford
Email: christian.becker@obs-gyn.ox.ac.uk

Mr Dimitrios Mavrelos
University College London Hospitals
Email: d.mavrelos@ucl.ac.uk

Professor Siladitya Bhattacharya

University of Aberdeen
Email: s.bhattacharya@abdn.ac.uk

Independent Trial Steering Committee

For independent oversight

Chair: Professor Mary-Ann Lumsden

University of Glasgow

Professor Ying Cheong (University of Southampton)

Dr Jayne Tullett (West Heath Medical Centre, Bham)

Mr Sanjay Vyas **(**British Society for Gynaecological Endoscopy)

Data Monitoring and Ethics Committee

For patient safety, interim analysis and response to specific concerns

Chair: Professor Lucy Chappell

(King's College London)

Dr Patrick Chien (Ninewells Hospital, Dundee)

Sally Kerry (Barts and The London School of Medicine and Dentistry

PRE-EMPT Trials Office

Birmingham Clinical Trials Unit (BCTU)
Institute of Applied Health Research
Public Health Building
University of Birmingham
Edgbaston
Birmingham B15 2TT
Telephone: 0121 415 9109 (Voicemail outside office hours)
Fax: 0121 415 9136
E-mail: pre-empt@trials.bham.ac.uk
Website: www.birmingham.ac.uk/pre-empt

Clinical queries should be directed during office hours to the Chief Investigator. Other queries should be directed to the PRE-EMPT Trials Office.

FOR RANDOMISATIONS

Telephone: 0800 953 0274 (UK)

Fax: 0121 415 9136

Website https://www.trials.bham.ac.uk/pre-empt

Protocol version

6.0 dated 06th October 2020

1.0 dated 25th July 2013 1.1 dated 1st November 2014

2.0 dated 23/10/2015

3.0 dated 18/05/2017

4.0 dated 05/04/2018

5.0 dated 19th August 2020

ISRCTN 97865475

EUDRACT Number 2013-001984-21

IRAS Number 101577

Funding Body

NIHR Health Technology Assessment Programme HTA no: **11/114/01**

Sponsor and Sponsor Roles

Sponsor protocol number **3/013/13**

Co-Sponsored between University of Aberdeen and NHS Grampian.

Professor Kevin Cooper is the Chief Investigator.

The University of Birmingham is responsible for obtaining necessary approvals and for pharmacovigilance, the Trial Management Group is jointly responsible for overseeing good clinical practice and all Clinical Investigators are responsible for obtaining informed consent and care of the participants.

Signature

The investigators and the sponsor have discussed this protocol. The investigators agree to perform the investigation and to abide by this protocol except in case of medical emergency or where departures from it are mutually agreed in writing.

Chief investigator

Professor Kevin Cooper

University of Aberdeen	Signature	Date

Principal Investigator’s signature

Site:	
Principal Investigator:	
Title:	The PRE-EMPT Trial: Preventing REcurrence of Endometriosis by Means of long acting Progestogen Therapy
Version:	7.0

I confirm I have received, read and understood the aforementioned version of the trial protocol. I confirm my team and I will adhere to this version of the protocol following receipt of the required local approvals.

Principal Investigator's name:	______________________________
Signature:	______________________________
Date:	DD / MM / YYYY

The Principal Investigator should sign this page and return a copy of this page to the PRE-EMPT Trial Office

PRE-EMPT Trial Office
Birmingham Clinical Trials Unit
Institute of Applied Health Research
University of Birmingham
Edgbaston
Birmingham
B15 2TT

Fax: 0121 415 9136
Email: pre-empt@trials.bham.ac.uk

Abbreviations

AE	Adverse event
AR	Adverse reaction
BCTU	Birmingham Clinical Trials Unit at the University of Birmingham
BMI	Body Mass Index
CI	Chief Investigator
CLRN	Comprehensive Clinical Research Network
COCP	Combined Oral Contraceptive Pill
DMEC	Data Monitoring and Ethics Committee
DMPA	Depot Medroxyprogesterone Acetate injection (Depo-Provera™)
EQ-5D-5L	Quality of Life Questionnaire
EudraCT	European Clinical Trials Database
EHP-30	Endometriosis Health Profile -30
FG	Focus Group
FSS	Fatigue Severity Scale
GCP	Good Clinical Practice
GnRHa	Gonadotropin releasing hormone analogues
GP	General Practitioner
HRQOL	Health Related Quality of Life
ICECAP-A	Capability Wellbeing questionnaire
ISRCTN	International Standard Randomised Controlled Trial Number
LARCs	long acting reversible contraceptives
LNG-IUS	Levonorgestrel-releasing Intra-Uterine System (Mirena™)
MHRA	Medicines and Healthcare Products Regulatory Authority
MRI	Magnetic Resonance Imaging
NIHR	National Institute for Health Research
PI	Principal Investigator – the local lead investigator for the PRE-EMPT Trial
PPI	Patient and Public Involvement
PIS	Participant Information Sheet
REC	Research Ethics Committee
RSI	Reference Safety Information
SAE	Serious Adverse Event
SAR	Serious Adverse Reaction
SAP	Statistical Analysis Plan
SD	Standard Deviation
SF36	Short Form Health Survey Questionnaire
SOP	Standard Operating Procedure
SmPC	Summary of Product Characteristics

SUSAR	Suspected Unexpected Serious Adverse Reaction
TMG	Trial Management Group
TSC	Trial Steering Committee

CONTENTS

1. BACKGROUND 1
   1.1. Endometriosis 1
   1.2. Medical therapies for prevention of recurrence 1
   1.3. The choice of questions to be asked 3
   1.4. PRE-EMPT internal pilot study 3
   1.5. Aims and objectives of PRE-EMPT 10
2. TRIAL DESIGN 11
   2.1. Design 11
3. ELIGIBILITY 11
   3.1. Setting 11
   3.2. Source and screening of potential participants 11
   3.3. Inclusion and Exclusion Criteria 12
   3.4. Identification, consent and randomisation of PRE-EMPT participants 12
4. RANDOMISATION 15
   4.1. Pre-registration 15
5. TREATMENT ALLOCATIONS 16
   5.1. Trial treatment available 16
   5.2. Prescription of PRE-EMPT trial treatments 17
   5.3. Participant withdrawal and changes of status within trial 18
   5.4. Compliance monitoring 19
   5.5. Excluded medications or interactions 19
   5.6. Other management at discretion of local doctors 20
6. SAFETY MONITORING PROCEDURES 20
   6.1. General Definitions 20
   6.2. Reporting AEs 22
   6.3. Reporting SAEs 22
   6.4. Reporting SUSARs 24
   6.5. Notification of deaths 24
   6.6. Monitoring pregnancies for potential Serious Adverse Events 24
   6.7. Pharmacovigilance responsibilities 25
7. FOLLOW-UP AND OUTCOME MEASURES 26
   7.1. Primary outcome measure 26
   7.2. Secondary outcome measures 26

7.3. Format and timing of trial assessments 27
7.4. Data management and validation 29
7.5. Retention strategies 30
7.6. Long Term Storage of Data 30
7.7. Long-term follow-up 31
7.8. Definition of the End of Trial 31
8. ACCRUAL AND ANALYSIS 31
8.1. Sample size 31
8.2. Statistical Analysis 31
9. ECONOMIC EVALUATION 33
10. DATA ACCESS AND QUALITY ASSURANCE 35
10.1. Confidentiality of personal data 35
10.2. Data Quality Assurance 35
10.3. Independent Trial Steering Committee 36
10.4. Data Monitoring and Ethics Committee 36
10.5. Long-term storage of data 37
11. ORGANISATION AND RESPONSIBILITIES 38
11.1. Centre eligibility 38
11.2. Local co-ordinator at each centre 38
11.3. Research nurses at each centre 38
11.4. The PRE-EMPT Trial Office 38
11.5. Research Governance 39
11.6. Regulatory and Ethical Approval 39
11.7. Funding and Cost implications 39
11.8. Indemnity 40
11.9. Publication 40
11.10. Ancillary studies 40
12. REFERENCES 41
13. APPENDICES 44
APPENDIX 1: PRE-EMPT TRIAL SCHEMA 45

1. BACKGROUND

1.1. Endometriosis

Endometriosis occurs when endometrium grows in abnormal locations such as the pelvic peritoneum, ovaries, fallopian tubes, bladder and bowel. Endometriotic deposits undergo cyclical proliferation in response to ovarian hormones (mainly oestrogen) resulting in internal bleeding and inflammation, followed by scarring and adhesion formation. This results in pain, and has a profound impact on quality of life(1, 2). The gold standard for the diagnosis of endometriosis is laparoscopy, a key-hole surgical procedure that allows direct visualization and biopsy of endometriotic tissue. Endometriosis affects up to one in ten women(3), poses a considerable socioeconomic burden(4) and has serious impact on the quality of life in affected women(1, 2, 5).

Surgical removal or destruction of endometriotic tissue is currently the preferred treatment for pain and other symptoms(6, 7) but the risk of recurrence is high. Relapse of symptoms occurs in 40-45% of women(6) and 27% of women are readmitted for surgery within 5 years(8). Half of all women diagnosed with endometriosis require a second operation and just over a quarter will undergo three or more procedures. Given the substantial cost, morbidity and prolonged recovery period associated with repeat surgery, there is an urgent need to identify an effective means of reducing the risk of recurrence of symptoms.

1.2. Medical therapies for prevention of recurrence

Reduction of pain due to recurrence involves the use of agents which reduce circulating levels of oestrogen, causing shrinkage of existing endometriotic deposits and prevention de novo lesions. A number of drugs are in current use but there is no consensus as to which is most effective and cost effective.

Gonadotropin releasing hormone analogues (GnRHa)(9), which reduces gonadotropin secretion have been shown to be more effective than no treatment(10) or danazol(11) and as effective as daily oral progestogen (dienogest)(12), combined oral contraceptive(13, 14) and LNG-IUS(15, 16). However, menopausal symptoms and loss of bone mineral density associated with GnRHa make them unsuitable for long-term use beyond 6 months. Although these drugs have been used with additional add-back hormonal therapy, there are insufficient data from randomised trials to support a specific regimen of add back therapy over others(17) In addition, this strategy is expensive and relatively uncommon in the UK.

There have been only a few small trials of the combined oral contraceptive pill (COCP) following conservative surgery(18, 19). A randomised comparison of continuous and cyclical COCP regimens found them equally effective insofar as postoperative pain and recurrence of endometrioma were concerned, although the continuous regimen was associated with significantly higher adverse effects and discontinuation rates(18).

Long acting progestogens, including depot medroxyprogesterone acetate injections (DMPA) and the levonorgestrel intrauterine system (LNG-IUS), have been shown to reduce the recurrence of symptoms after surgery for endometriosis and possess the advantage of less frequent administration. They are safe, acceptable, and relatively inexpensive and have few systematic side-effects(20, 21). Both have a prolonged duration of action, thus eliminating the need for daily administration and potentially improving compliance. DMPA, administered as a three monthly injection is less reliant on patient compliance than daily oral regimens but menstrual irregularity and weight gain are common problems while there is some

concern that its prolonged use, over 5 years, might lead to bone loss (albeit reversible on discontinuation). A single LNG-IUS can be effective for up to five years and small randomised trials(21, 22) have suggested that, despite symptoms of irregular bleeding in a fifth of women, LNG-IUS could be effective in reducing post-operative pain in women with endometriosis.

1.2.1 Commissioning brief

The NIHR Health Technology Assessment Programme issued a commissioning brief for UK researchers to design a randomised trial to address the following research question:

What is the clinical and cost-effectiveness of long acting reversible contraceptives (LARCs) in preventing recurrence of endometriosis?

The proposed interventions were the LNG-IUS, DMPA and progestogen implants, for which applicants were asked to justify their choice. The population was suggested to encompass women with endometriosis whose presenting symptoms have come under control by primary medical or surgical treatment and who do not wish to become pregnant in the next year, recruited from either primary or secondary care. The brief indicated the comparison group should be usual treatment, e.g. oral progestogens or GnRHa, again asking the applicants to define. Recurrence of symptoms had to be the primary outcome, although the definition and criteria was to be specified by the applicants, alongside adverse events, cost-effectiveness. Other outcomes that could be considered included haemoglobin levels, pain control, menstrual bleeding, health-related quality of life, patient satisfaction, acceptability, and adherence to treatment. The participants had to be followed up for a minimum of three years.

1.2.2 Rationale

The tendency of endometriosis to recur after surgery leads to renewed pain, repeated surgery and deterioration of quality of life. Existing data suggest that recurrence can be controlled by post-surgical hormonal treatment but there is uncertainty as to whether some of these treatments are better than others. A Cochrane review did not find sufficient evidence in favour of drug treatment after conservative surgery(23) however trials included in this review had small sample sizes and suboptimal follow up.

Initial reports suggest that LARCs are potentially useful in this context. A Cochrane review(24) of post-surgical use of LNG-IUS in women with endometriosis identified one small randomised controlled trial(21) comparing LNG-IUS with expectant management. Women on LNG-IUS reported fewer painful periods than those managed expectantly (OR 0.14, 95% CI 0.02 to 0.75). More women in the LNG-IUS group (75%, 15/20) were satisfied than in the non-treated group (50%, 10/20) but the difference did not reach statistical difference (OR 3.00, 95% CI 0.79 to 11.44). Data from small populations enrolled in more recent trials have suggested that LNG-IUS was better than no treatment(22) and as effective as DMPA but associated with better compliance and bone density(25). Relevant Cochrane reviews have called for well-designed, adequately powered randomised controlled trials to investigate the comparative effectiveness of LNG-IUS with other long term hormonal treatments.

A survey of members of the British Gynaecological Endoscopy Society has confirmed the lack of consensus about the use of post-surgical medical treatment to prevent recurrence(18). Of those surveyed, 24% use no post-operative treatment. Of the 76% who do, the commonest are LNG-IUS, COCP and DMPA. Relatively few use oral progestogens and very few use long term GnRH analogues. The comparisons felt to be most clinically

relevant for a clinical trial were LNG-IUS versus COCP (42%), LNG-IUS versus no treatment (38%) and LNG-IUS versus DMPA (27%).

It is critical to provide data from a definitive randomised trial on which treatment is the most effective in terms of preventing recurrence of symptoms of endometriosis in terms of symptom relief, side-effects, acceptability and costs. This information will be vital in terms of future clinical decision making in an area of uncertainty. A search of the WHO Clinical Trials Register revealed one small on-going trial (n=112; NCT01056042) comparing DMPA against COCP, and two comparing COCP against GnRHs. The magnitude of the problem, health burden, genuine uncertainty, feeling of equipoise among practitioners, lack of any other similar trials all highlight the reasons why a trial is urgently needed at the present time.

1.3. The choice of questions to be asked

The commissioning brief asked what is the effectiveness of long acting progestogens in preventing recurrence of endometriosis? The survey demonstrated that both LNG-IUS and DMPA are widely used, and that there is no consensus on non-long acting progestogen treatment, with both COCP and no post-operative treatment also frequently used as preventative strategies.

There are potentially a number of comparisons, considering the four treatment groups.

1. What is the effectiveness of any long acting progestogen compared with other drugs?
2. What is the effectiveness of any long acting progestogen compared with no treatment?
3. What is the effectiveness of LNG-IUS or DMPA compared with no treatment, or COCP.
4. Assuming long acting progestogens are more effective than no treatment, which is better?

1.4. PRE-EMPT internal pilot study

1.4.1 Pilot study objectives

We completed an internal pilot study in a restricted number of centres (six), over the course of a year from March 2014, with the aim of recruiting 100 women prior to expansion to the full study.

The pilot study aimed to assess the potential impact of strong patient preferences on recruitment and to collect information to help ensure the smooth running of the full study. The objectives were -

- To ascertain if randomising to one or more of the four treatment options will prove a barrier to overall recruitment, or conversely, suggest a four-way randomisation is feasible
- To pilot and fine-tune operational procedures, data capture forms and the assumptions around the sample size estimation
- To understand which factors motivate women to agree to be randomised for follow up treatment following surgery for endometriosis
- To explore barriers to recruitment to the trial

During the pilot phase, a ‘flexible entry’ design was employed, where an eligible participant can be randomised to two, three of four of the treatment options Figure **1**. At least one of the treatment options had to be a long acting progestogen (LNG-IUS or DMPA) whilst at least one of the other options available for randomisation had to be either COCP or no treatment to ensure compliance with the question posed in the commissioning brief.

Figure 1: Trial Schema

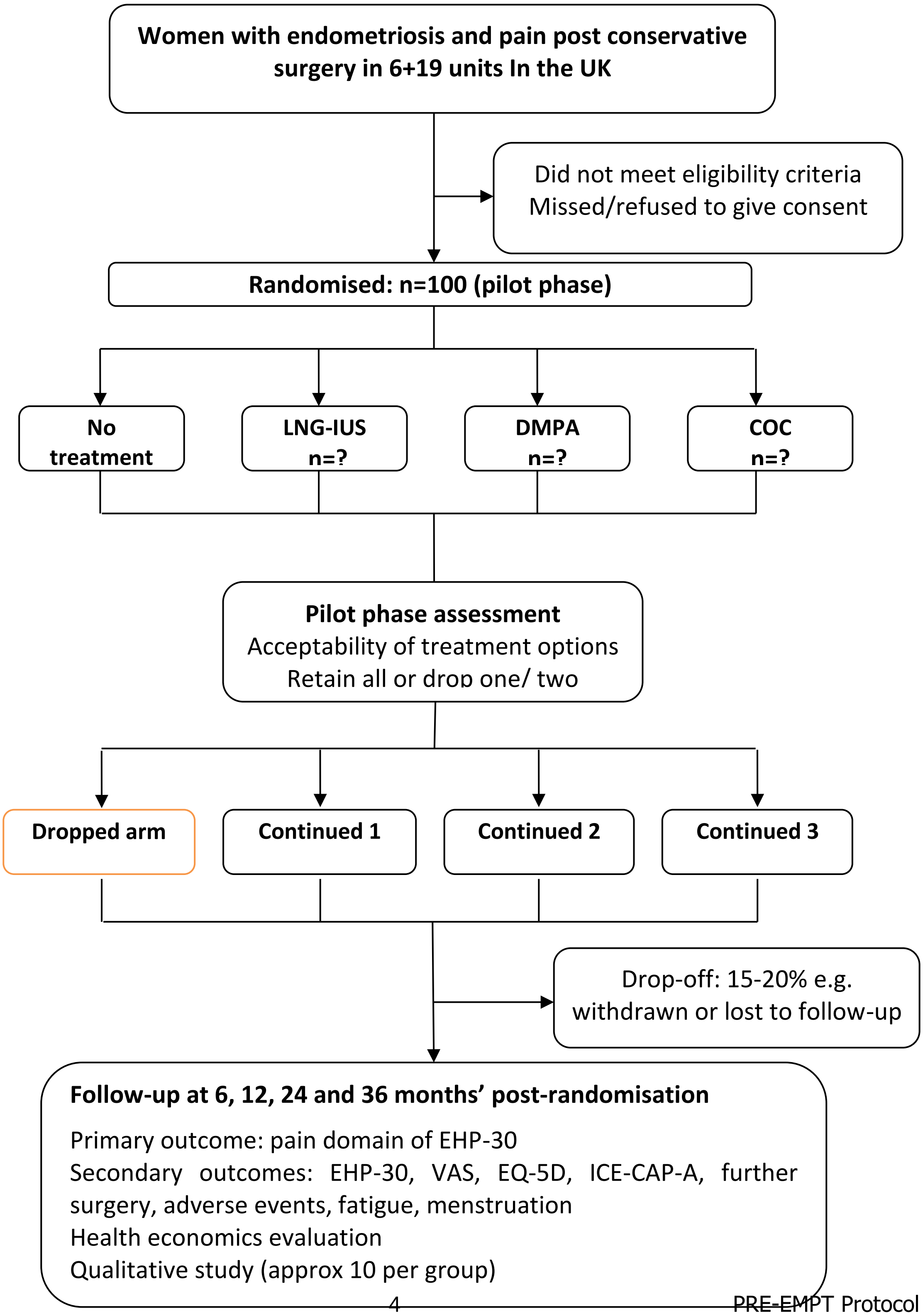

1.4.2 Decisions to be made after the pilot phase

Before the start of the PRE-EMPT Trial, we anticipated there will be significant preferences for or against certain treatment groups, from both clinician and women, and the pilot study was designed with a flexible randomisation option to accommodate these. We anticipated being faced with a number of scenarios

- a) Poor recruitment to one group eg 10%:30%:30%:30%
- b) Poor recruitment to two group eg 10%: 10%; 40%: 40%
- c) Equal recruitment to each group, although via balanced 3-way randomisation
- d) Equal recruitment to each via 4-way randomisation

For scenarios c) and d) we could continue with the 4-way randomisation into the main trial and have more than enough power to address questions 1 and 2 above. For scenario 1, we would drop the group only recruiting 10% of the pilot sample. For scenario 2, we would drop both 10% groups, provided the remaining two groups included one long acting progestogen and one non- long acting progestogen. If only long acting progestogen and DPMA remained viable options, we would discuss with the HTA how this fits with the commissioning brief.

At the commencement of the trial, we could not anticipate which arm may be dropped. If it were the no treatment group, question (2) and some comparisons within option (3) would be eliminated and COC would become the sole comparator for the long acting progestogens. This would have significant implications for the sample size calculation.

The decision would be based on acceptability of the treatment comparisons and not on the primary outcome data, so would not incur alpha spending to accommodate interim analyses. A report was prepared at the end of the pilot for review by the independent TSC-DMC committee. This report included information on recruitment, the randomisation options chosen and results of a qualitative assessment of barriers and facilitators to recruitment.

1.4.3 Results of the internal pilot phase – randomisation options

Seventy-seven patients were recruited to the pilot by the end of March 2014. The following randomisations options were chosen:

Patients preference	Number	Allocation			
		LNG-IUS	DMPA	COCP	None
LNG-IUS v COCP	11	5	X	6	X
LNG-IUS v None	11	6	X	X	5
LNG-IUS v COCP v None	5	2	X	1	2
DMPA v COCP	14	X	6	8	X
DMPA v None	10	X	5	X	5
DMPA v COCP v None	12	X	4	4	4
LNG-IUS v DMPA v COCP	3	1	1	1	X
LNG-IUS v DMPA v None	6	1	3	X	2
All treatments	5	1	1	2	1
Total	77	16	20	22	19

The following was notable:

- A very small proportion - 5/77 (6%) – were happy to be randomised to all four treatment options.
- A small proportion - 14/77 (18%) - selected 3-way randomisations including both LARCs. 27/63 (43%) had a preference for LNG-IUS and 36/63 (57%) a preference for DMPA.
- A small proportion - 22/77 (29%) – selected 3-way randomisations including both COCP and no treatment. 28/55 (51%) had a preference for COCP and 27/55 (49%) a preference for no treatment.
- The majority - 46/77 (60%) - expressed a preference for both a particular LARC and a particular non-LARC.

1.4.4 Results of the internal pilot phase – qualitative report

Methods: a focus group discussion and individual interviews were conducted. The focus group discussion took place at Aberdeen Royal infirmary and was facilitated by two members of the research team. Initially six women agreed to take part, but only four attended. Three women were interviewed in person (from Birmingham) and seven were interviewed over the telephone (from Edinburgh and Aberdeen) (n=10). Telephone interviews offered a flexible means to include women from a wide geographical spread. Individual interviews allowed respondents to feel more relaxed and able to address issues concerning their endometriosis treatment experiences that may have been too sensitive to discuss in a focus group setting(26). An interview schedule was adapted from the focus group discussion guide, which elicited women's past experiences with the treatments included in the trial and examined their willingness to accept each treatment and whether their inclusion in any of them would constitute a barrier to continuation within the study.

All of the women had been randomised in the RCT and had given additional consent for the qualitative study. The ages of the women recruited to the focus group and interviews ranged from 19 to 36 years, and experience of symptoms varied from 2.5 to 16 years. There was a range of symptomology, treatment histories, and allocated treatment groups within the trial.

The focus group and interviews were recorded with consent and transcribed verbatim. Content analysis(27) was employed, with the research team (a qualitative lead and two qualitative assistants) each independently reading the transcripts and then agreeing upon common patterns and themes from women's experiences and perspectives on treatment acceptability. Dissident views and areas of diversity were also considered

Results - views on randomisation and decision-making regarding treatments: Women found the strategy of flexible randomisation acceptable as they had an element of choice. Half of the participants (n=7) reported that without the option to opt-out of a particular treatment group (or groups), they would have declined trial participation. Two women viewed randomisation positively as it relieved them from the burden of choosing a treatment option without adequate knowledge of the options. As discussed below, findings from the focus group and interviews demonstrate that no one treatment group was more or less acceptable to women. Participants had individual preferences for which post-surgery treatments they would find acceptable based on their own experiences of previous treatments and those of significant others. In some instances, not having experience of a particular treatment was seen by women as a reason for its acceptance.

Results - views on individual treatment arms: Women had complex views on whether treatments used prior to surgery would be more effective post-surgery. For example, a few women (n=3) had negative past experiences with both the pill and injection. While they would not accept the injection post-surgery because of unpleasant side-effects, they were more tolerant of the pill on the grounds that it could be more effective post-surgery following the removal of endometriosis tissue. Some women (n=3) who had previously undergone surgery with (ineffective) post-operative hormone use in the past did not believe any particular hormonal treatment would be more effective after surgery.

- ***Pill (COCP):*** Women who did not want to go on the pill based their decision on the fact that it had been ineffective previously. Women who accepted the pill did so because of ease of use, ability to discontinue it if necessary and previous success. Participants who expressed ambivalence about the pill did so because despite limited success with it in the past, they believed that a new preparation or post-surgical use might make it more effective.
- ***Coil (LNG-IUS or copper IUD)***: Women who viewed the coil as an unacceptable treatment option did so because of past negative experiences of their own (including discomfort, pain, poor fittings and infections) or those of friends and family members. One woman was willing to try it in the context of this trial despite previous problems with it, because what was on offer was perceived to be a "new, smaller coil releasing a lower level of hormones". Women who found the coil acceptable had no previous experience of it and/or appreciated its convenience.
- ***Injection (DPMA)***: Participants who rejected the depot injection did so because it had been ineffective in the past, resulted in symptoms such as heavier/ more frequent periods and migraines, and/ or required repeated injections. Acceptance of the depot injection was encouraged by the fact that it was a new, previously untried option.
- ***No Treatment***: Women who found the no-treatment arm unacceptable were either concerned that their endometriosis symptoms would return more quickly post-surgery or required hormonal contraception. Those who found no-treatment acceptable reported feeling they were willing to let their body 'have a break' from hormonal treatments and to 'let [their] body settle' after surgery and to see the efficacy of the surgery in relation to their symptoms. One woman enrolled in the no-treatment arm expressed concern that she could require a hormonal contraception if her life circumstances changed.

Results – views on trial participation, worthiness, and length: Women chose to participate in the trial for reasons of altruism and self-interest. Participants expressed a hope to help other women with endometriosis and to prevent others from 'suffering' from the same physical, emotional, and health consequences they had experienced. A few women reported enrolling in the trial out of 'desperation' and a willingness to 'try anything' to manage their condition. Other women hoped that surgery and treatment would help manage their conditions so they could gain some control over their condition and to 'get their life back.' Additionally, women enrolled in the trial for other reasons, such as a desire to raise awareness of endometriosis among the public and health professionals and to speed up diagnosis and treatment. In relation to the trial's main purpose, to assess the efficacy of different hormonal treatments and non-treatment post-surgery, women found this to be a worthwhile research question and aim.

Overwhelmingly, women found the three-year length of their participation acceptable. Women thought that a long study reflects the chronic nature of the disease as well as its unpredictable symptoms that vary over different time periods in one's life. They viewed the relatively long time period as a positive both for themselves as individuals (to monitor the efficacy of their post-surgery treatment over the course of three years) and for the overall success of the trial. Women felt it took time for their bodies to 'get used to' hormonal treatments (or the absence of hormonal treatment) and for negative side-effects to subside. Women who had undergone previous laparoscopic surgery felt surgery alone reduced pain for a period of two to three years. Thus, the trial length was seen as advantageous as it allows both for the efficacy of post-surgery treatments to be considered in light of this 'adjustment period' and for the decline in surgery effectiveness of pain management over time. Only one woman expressed a concern about the trial length, stating that some participants might wish to become pregnant and withdraw from the trial.

Conclusion: These data, based on a sample of women who had agreed to be randomised, suggest that women found flexible randomisation acceptable. Half of participants reported that they would decline the trial if they had not been allowed this element of choice over treatment options. No single post-surgical treatment option (including the no-treatment group) was found either more or less acceptable by participants.

1.4.5 Emerging evidence

Since the original application for funding was submitted two systematic reviews of variable quality have examined the use of the postoperative combined oral contraceptive (COCP) use on recurrence of endometriosis. Wu et al, (2013)(28) identified 15 randomised trials including 850 patients. The combined odds of recurrence [OR = 0.31, 95% CI (0.22, 0.45), p < 0.00001] was noted to be lower in the COC group compared with surgery alone. In a second systematic review Vercellini et al, (2013)(29) evaluated the use of prolonged (at least 2 years) postoperative COC use on endometrioma recurrence in a total of 965 women (726 in cohort studies and 239 in one randomized controlled trial). Recurrence was identified in (8%) COC users versus (34%) women who underwent no treatment (pooled odds ratio 0.12; 95% confidence interval 0.05-0.29). A dose dependent effect was identified with a pooled odds ratio of, 0.21 (95% confidence interval 0.11-0.40) and 0.39 (95% confidence interval 0.23-0.66) respectively for "always" versus "ever" users, and "ever" with "never" users.

1.4.6 Proposal for full trial design

We ruled out the following randomisation designs from the larger study due to low numbers selecting these randomisation options:

i) 4-way randomisation
ii) Any 3-way randomisation with both LARCs as options (i.e. LNG-IUS v DMPA v no treatment)
iii) Any 3-way randomisation with both non-LARCs as options (i.e. DMPA v COC v no treatment)
iv) A 2-way randomisation involving solely the most commonly selected single randomisation option (DMPA v COCP) as this has only attracted only 14 participants (19%).

Given the strong preferences in the internal pilot and the feedback from the qualitative work our chosen design is pragmatic and incorporates some element of patient/clinician choice.

The main comparison will be LARC v COCP, with sub-comparisons of LNG-IUS v COCP and DMPA v COCP (i.e. where the intention is to treat with LNG-IUS or DMPA if randomised to LARC). COCP has been chosen as the comparator given the recent published reviews that suggest COCP is more effective than no treatment.

Figure 2: Proposed trial design following internal pilot

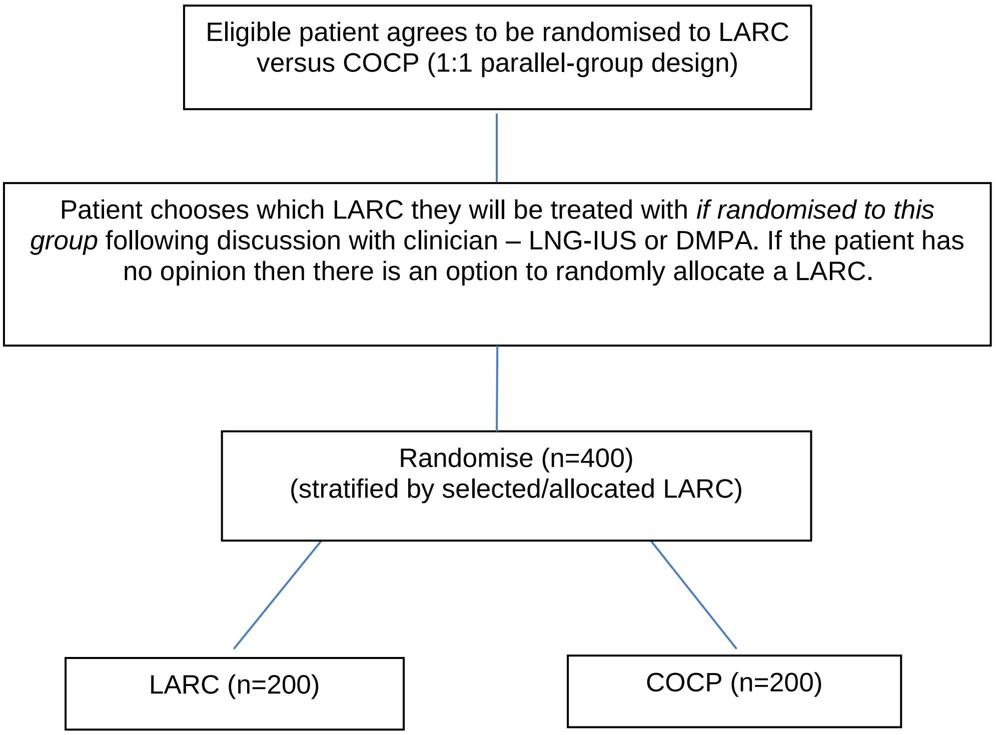

Primary comparison: LARC v COCP (n=400)

Sub-comparison 1: LARC v COCP in those who chose LNG-IUS (n=approx. 200)

Sub-comparison 2: LARC v COCP in those who chose DMPA (n=approx. 200)

Further details on the calculations for the sample size are given in section 8.1

1.5. Aims and objectives of PRE-EMPT

1.5.1 Study aim:

To evaluate the clinical effectiveness and cost effectiveness of long acting reversible contraceptives (LARCs: LNG-IUS or DMPA) compared with the combined oral contraceptive pill (COCP) in preventing the recurrence of endometriosis.

1.5.2 Primary objective:

- To compare, in women undergoing conservative surgery for pain due to endometriosis, the effectiveness of LARCs compared to COCP in preventing the recurrence of symptoms and improving quality of life.

1.5.3 Secondary objectives:

Clinical:

- To compare LARCs versus COCP as per the primary objective in those that selected LNG-IUS as their method of delivery.
- To compare LARCs versus COCP as per the primary objective in those that selected DMPA as their method of delivery.
- To compare LARCs versus COCP in terms of pain relief, serious side-effects (section 6.1) and repeat surgery.

Economic:

- To compare the relative cost effectiveness of alternative hormonal interventions, DMPA, LNG-IUS and COCP for the prevention of recurrent endometriosis. The main comparator will be COCP. The evaluation will have two principal components:

a. To collate the cost and effectiveness evidence available from existing research, systematic reviews and routine health administrative sources to provide data for a pre-trial decision analysis model based on the design of the proposed trial.
b. To use prospectively collected resource use data - on resource use associated with the alternative treatment pathways, outcomes in terms of quality of life (EQ-5D-5L and ICECAP-A) and reported symptoms such as pain and cost data collected alongside the trial where necessary, to evaluate the cost and cost effectiveness of the alternative strategies in a model based economic evaluation based on the trial.

2. TRIAL DESIGN

2.1. Design

A randomised, pragmatic multicentre trial with integrated economic evaluation.

3. ELIGIBILITY

3.1. Setting

Patients will be recruited from the gynaecological, out-patient clinics of participating centres, fitting around their current service provision. Long term medical treatment i.e. repeat COCP prescriptions and DMPA injections, will be delivered by the participants GP or sexual health clinics, as per current practice. Recruitment will be supported by dedicated research nurses, who will work with local gynaecology leads and liaise with a research nurse coordinator.

3.2. Source and screening of potential participants

The target population will be women of reproductive age with laparoscopically diagnosed endometriosis, who have undergone conservative surgery for alleviation of pelvic pain, who have no contraindications to any of the proposed hormonal treatments (see exclusion criteria section 3.3.2 below). The PRE-EMPT trial will be discussed with women when they are scheduled for laparoscopic surgery.

All women over the age of 16 and below 46 years will be considered, where long term medical treatment following ablation/ excision of endometriosis might be reasonably considered. Young women under the age of 16 will not be eligible because of the need to seek parental consent. Diagnosis in minors is rare, whilst symptoms tend to spontaneously resolve as menopause approaches.

In women presenting with pelvic pain associated with endometriosis, anatomical location, severity of the disease and degree of involvement of neighbouring organs can all show a remarkable degree of variation. Women who are to have radical surgical treatment such as hysterectomy and or removal of both ovaries will not be approached for participation.

Final confirmation of eligibility will be established at laparoscopy. There will be no restriction in terms of disease severity or staging: all women who have undergone conservative surgery, where the aim is to excise or ablate areas of endometriosis and dissect pelvic adhesions, will be eligible. Women undergoing complex surgery or hysterectomy will be excluded. The nature and extent of surgery in this context is dictated by the operative findings and it is therefore very difficult to be prescriptive about the exact nature of surgery.

Histological confirmation of endometrial lesions will not be required prior to randomisation, as this is not always undertaken and ablation/ excision proceeds on the basis of visual inspection. It is well documented that the revised American Fertility Society score, the most widely used rating scale, is not significantly correlated with the degree of pain experienced(30). The prevailing criteria will be that long term medical treatment might reasonably be considered and no immediate, further surgery is considered necessary e.g. bowel resection.

3.3. Inclusion and Exclusion Criteria

3.3.1 Inclusion criteria

- Women aged 16-45
- No immediate plans to conceive
- Are scheduled to have laparoscopic conservative surgery, or a diagnostic laparoscopy with concurrent surgery if endometriosis is found, for pelvic pain associated with endometriosis.
- Willing to be randomised to one long acting progestogen (LNG-IUS or DMPA) and COCP

The following women are all eligible for PRE-EMPT if they have recurrent pain and are to have conservative surgery for endometriosis:

- Have had one or more previous diagnostic laparoscopies
- Have had previous laparoscopic conservative surgery for endometriosis, providing this did not involve rectovaginal dissection or bowel resection.
- Have used post-operative medical treatment, including the treatment options included in PRE-EMPT. The choice of randomisation options may reflect previous treatments.
- Previous use of treatment options included in PRE-EMPT as contraceptives. It is acceptable to randomise women currently taking the COCP to continue with this or switch to LNG-IUS or DMPA.
- Use of pre-operative GnRHa, providing this is stopped at least 4 weeks' prior to laparoscopy.

3.3.2 Exclusion criteria

- No endometriosis identified at diagnostic laparoscopy
- Infertility
- Any plans for further elective endometriosis surgery (for deep infiltrating disease or any other cause).
- Contraindications to the use of hormonal treatment with oestrogen or progestogens
- Suspicion of malignancy

3.3.3 Eligibility and randomisation incorporating LARC choice.

Given our internal pilot study results, LARC treatment preferences are anticipated. Women may also have taken a LARC treatment previously and wish to avoid that specific treatment again. Women will be asked to specify which LARC they would like to receive if randomised to this treatment arm. If no opinion is offered there will be an option to randomly allocate a LARC.

3.4. Identification, consent and randomisation of PRE-EMPT participants

Women with symptoms suggestive of endometriosis are referred to general or specialist gynaecological clinics, where their history is taken, a clinical assessment performed and sometimes an ultrasound or MRI scan. In some units, a diagnostic laparoscopy will be performed to establish the presence and extent of endometriosis before definitive surgery. Other gynaecologists use a "see and treat" approach to laparoscopically diagnosed endometriosis.

3.4.1 Approaching potential participants for consent

All women being scheduled for conservative surgery will be approached, **before the procedure** with information regarding post-operative options for prevention of recurrence and the PRE-EMPT trial. The research nurses may contact potentially eligible participants by telephone to discuss the study, if it looks like they may meet the eligibility criteria. All women approached should be recorded on the paper screening log, (available in the site file) or electronically.

It will be the responsibility of the Investigator to obtain written informed consent for each participant prior to performing any trial related procedure. Research nurses/midwifes and non-clinical research staff who are appropriately trained and to whom the duty has been delegated by the Principal Investigator (PI) will perform duties to include aspects of local organisation including identifying, consenting, and randomising the participants. This will be evidenced on the site delegation log

A Participant Information Sheet (PIS) will be provided to facilitate this process. Investigators (or delegated person) will ensure that they adequately explain the aim, trial treatment, anticipated benefits and potential hazards of taking part in the trial to the participant. They will also stress that participation is voluntary and that the participant is free to refuse to take part and may withdraw from the trial at any time. The participant will be given sufficient time to read the PIS and to discuss their participation with others outside of the site research team. The participant will be given the opportunity to ask questions. If the participant expresses an interest in participating in the trial they will be asked to sign and date the latest version of the consent form. The participant must give explicit consent for the regulatory authorities, members of the research team and or representatives of the sponsor to be given direct access to the participant's medical records".

The Investigator (or delegated person) will then sign and date the Informed Consent Form (ICF). A copy of the ICF will be given to the participant, a copy will be filed in the medical notes, and the original placed in the Investigator Site File (ISF). Once the participant is entered into the trial, the participant's trial number will be entered on the ICF maintained in the ISF. In addition, if the participant has given explicit consent, a copy of the signed ICF will be sent to the Birmingham Clinical Trials Unit (BCTU) trials team for review".

Details of the informed consent discussions will be recorded in the participant's medical notes. This will include date of discussion, the name of the trial, summary of discussion, version number of the PIS given to participant and version number of ICF signed and date consent received. Where consent is obtained on the same day that the trial related assessments are due to start, a note should be made in the medical notes as to what time the consent was obtained and what time the procedures started.

At each follow up time point, the participant's willingness to continue in the trial will be ascertained and documented in the medical notes. Throughout the trial the participant will have the opportunity to ask questions about the trial. Any new information that may be relevant to the participant's continued participation will be provided. Where new information becomes available which may affect the participants' decision to continue, participants will be given time to consider and if happy to continue will be re-consented. Re-consent will be

documented in the medical notes. The participant's right to withdraw from the trial will remain.

Electronic copies of the PIS and ICF will be available from the Trials Office and for UK trials will be printed or photocopied onto the headed paper of the local institution.

Where LNG-IUS is an available randomisation option, and the investigator conducting the surgery intends to fit the system during the laparoscopic procedure if the participant is allocated to the LNG-IUS group, the women should be made aware of this possibility and asked for consent pre-operatively.

3.4.2 Ineligible and declining patients

Details of all patients approached about the trial will be recorded on the Participant Screening/Enrolment Log

3.4.3 Routes to randomisation

There will be two routes to randomisation:

- Randomisation post-operatively, prior to discharge.
- Randomisation intra-operatively, to enable those receiving LNG-IUS to have it inserted whilst under general anaesthetic.

The route will be determined by the investigator, according to their intention to fit the LNG-IUS, if allocated LARC, at the end of the conservative surgery.

Where the extent of endometriosis found at laparoscopy means that further surgical procedures are required e.g. bowel resection, the women is no longer eligible for PRE-EMPT. This should be sensitively explained to the women, and recorded on the screening log. All communication surrounding the ineligibility will be noted in the woman's hospital records and trial database, and no further data will be collected for that woman. The consent forms and paper data collection forms will be stored securely in the Investigator Site File (ISF), separately from the eligible randomised patients. These forms will be available to various regulatory bodies for inspection upon request.

3.4.4 Organisation of recruitment

Based on our experience of conducting similar trials (e.g. OPT Study) we believe that the following strategy is likely to be successful in achieving maximum recruitment.

We propose to engage Comprehensive Research Networks to provide support to the trial. As the trial will automatically be included in the NIHR CRN portfolio, speciality group or theme leads for each CLRN will be approached to provide dedicated research nurse support for the trial.

The PRE-EMPT nurse coordinator for the region will visit each centre to support initiation of the trial and maintain regular contact with the CLRN nurses. We will negotiate comparable support for Trusts in Wales, Scotland and Northern IrelandWe will provide simple written trial information to eligible women, summarised on the first page with more detailed information for those interested, supported by face to face discussion with CLRN nurses and their gynaecologist.

We intend to identify all those who are eligible, have received information, consented or declined participation through coloured stickers on the hospital notes. We plan to ensure regular, close communication with gynaecological and nursing staff in the outpatient clinic and post-operative wards. Regular feedback on progress in study recruitment, including individual hospital teams' performance and progress against targets, will be provided.

4. RANDOMISATION

4.1. Pre-registration

Randomisation will be in two stages to enable rapid intra-operative completion of the process where investigators intend to fit the LNG-IUS, if allocated, at the end of the conservative surgery. Randomisation notepads will be provided to investigators and may be used to collate the necessary demographic and historical information prior to randomisation

Once pre-operative eligibility criteria are confirmed and consent for the trial is obtained, the participant can be pre-registered for PRE-EMPT. Part A of the Randomisation Notepad can be completed, either online, or by telephone to the randomisation service. This can be undertaken once consent has been obtained but does not require the participant to be present.

4.1.1 Randomisation

Randomisation will occur either intra-operatively, or immediately post-operatively, according to the randomisation options and the intention of the investigator. The stage of endometriosis, the need for additional surgery and the extent of the surgical excision or ablation of the endometriosis are the remaining eligibility criteria and stratification variables that can only established at laparoscopy.

Participants can be randomised into **PRE-EMPT** via a secure 24/7 internet-based randomisation service (https://www.trials.bham.ac.uk/pre-empt) or by telephone (number 0800 953 0274).
Telephone randomisation is available Monday - Friday, 09:00-17:00. Online randomisation is available 24 hours a day, 7 days a week, apart from short periods of scheduled maintenance and occasional network problems. For the secure internet randomisation, each site and each researcher will be provided with a unique log-in username and password.

Randomisation Forms will be provided to investigators and should be completed and used to collate the necessary information *prior* to randomisation.

The inclusion and exclusion criteria included on the Randomisation Form must be answered before a Trial Number can be given.

Once a Trial Number has been allocated, a confirmatory e-mail will be sent to the local PI, the named RN and the pharmacist (for reference).

4.1.2 Back-up Randomisation

If the internet-based randomisation service is unavailable for an extended period of time, a back-up paper randomisation service will be available from BCTU. In this instance, investigators should ring the BCTU randomisation service (**0800 9530274**). The randomisation list will be produced using a random length block design.

If the patient has been pre-registered the details are brought up by searching for the participant's name and the final eligibility and stratification data are recorded. The treatment group allocation is concealed from the healthcare providers until this final information is collected, and then the participant is randomised.

A 'minimisation' procedure using a computer-based algorithm will be used to avoid chance imbalances in important stratification variables. The variables chosen are:

- Stage of endometriosis (using Classification of the American Society of Reproductive Medicine): I (minimal) II (mild) versus III (moderate)/ IV (severe)
- Extent of excision of endometriosis: complete versus incomplete, as judged by the surgeon at the time of conservative surgery
- Age in years:<35 versus >=35
- Selection of LNG-IUS or DMPA if randomised to LARC
- Whether selection of LARC was due to patient preference or not
- Centre, to balance for experience of the gynaecologist

If the LARC needs to be randomly allocated prior to LARC v COCP allocation this will be completed using a random blocked list (variable length) incorporated into the computer-based algorithm.

4.1.3 Informing the participant's GP

With the Woman's prior consent the GP will be notified that she is participating in the PRE-EMPT trial and the treatment group to which she has been allocated (a template "letter to GP" is supplied. This is in addition to any discharge letter detailing repeat prescription requirements etc.

5. TREATMENT ALLOCATIONS

5.1. Trial treatment available

The three hormonal trial treatments are all licensed as contraceptives, but whilst widely used for the prevention of recurrence of endometriosis, are not specifically licenced for this purpose.

5.1.1 Depot medroxyprogesterone acetate injection (DMPA; Depo-Provera™)

Depo-Provera™ is an aqueous suspension of 150mg of depot medroxyprogesterone acetate. It is a long acting reversible preparation that is administered by intramuscular injection every three months. It is approved for use as a contraceptive is used off-label for the management of endometriosis-related pain. The most common side effects are menstrual irregularities (bleeding or amenorrhea), prolonged delay in the resumption of ovulation, weight changes. There is a potential risk of bone demineralisation with long-term use.

DMPA is licenced as a contraceptive and acts by preventing ovulation.

5.1.2 Levonorgestrel releasing intrauterine system (LNG-IUS; Mirena™/Levosert)

The LNG-IUS is a contraceptive system that slowly releases a daily dose of 20 mcg levonorgestrel into the uterine cavity. Bayer Pharma AG market their LNG-IUS under the name of Mirena™ and Actavis UK Ltd under the name of Levosert It is a long acting reversible preparation that requires removal and reinsertion every five years, 3 years for

Levosert. LNG-IUS is approved for use as a contraceptive and for heavy menstrual bleeding, prevention of endometrial hyperplasia during oestrogen replacement therapy but is widely used in management of endometriosis-related pain. The most common side effect is unscheduled menstrual bleeding, although this often resolves within 6 months. Documented risks include uterine perforation and infection. The LNG-IUS can also be fitted in primary care.

LNG-IUS has an anti-proliferative effect on the endometrium and can prevent ovulation, which may be the mechanism by which it prevents recurrence of endometriosis.

5.1.3 Combined oral contraceptive pill

PRE-EMPT will require that women allocated the combined oral contraceptive (COCP) are prescribed a COCP containing 30mcg ethinylestradiol and 150 mcg levonorgestrel e.g. Microgynon-30 or Rigevidon. This preparation acts systemically to inhibit ovulation and inhibit endometrial growth. Microgynon-30 is the most commonly prescribed COCP, accounting for 41% of all COCP prescriptions, and is one of the cheapest ethinylestradiol preparations. For the management of endometriosis-related pain, it is unclear whether combined oral contraceptives should be taken conventionally, continuously or in tricycle regimen, or whether the dose of ethinylestradiol, or type of progestogen, are important. We will record whether Microgynon-30/Rigevidon is used as a 21-day regimen or continuously. The side effects and risks of combined oral contraceptives have been well investigated(31). Combined oral contraceptives are not recommended in smokers aged ≥35 and women with a BMI ≥35.

Like the other PRE-EMPT treatments, the proposed COCP preparations act via anti-proliferation and anti-ovulatory actions, in comparable ways to DMPA and LNG-IUS.

5.2. Prescription of PRE-EMPT trial treatments

The administration of the initial medical treatment will depend both on the treatment group allocation and local policy. The intention should be to initiate the allocated treatment as soon as possible, ideally before discharge, to minimise non-compliance and for the convenience of the participant.

PRE-EMPT is a pragmatic trial and all treatments will be prescribed from local NHS stock according to local NHS practice. This includes the resupply of treatment being conducted in local hospitals, community pharmacies and primary care using standard routine prescribing processes.

The treatment resupply in the trial is pragmatic and reflective of NHS practice, drug accountability and labelling will not be required allowing for resupply of treatment to be conducted in local hospitals and community pharmacies following routine prescriptions being issued.

5.2.1 DMPA injection

Ideally, the first injection should be given before discharge by the gynaecology investigator. If the hospital does not stock DMPA, the participant should be given a prescription letter for her GP or the local sexual health clinic and encouraged to attend to receive the first injection as soon as possible.

If DMPA is given within the five days of the onset of menstruation it will provide immediate contraceptive cover, otherwise barrier methods must be used for 14 days.

Subsequent injections should be scheduled for every 12 weeks, to be delivered by the gynaecologist, GP or sexual health clinic as appropriate. If the interval from the preceding injection is greater than 89 days (12 weeks and five days) for any reason, then pregnancy should be excluded before the next injection is given and the women should use barrier methods for fourteen days after this subsequent injection.

5.2.2 LNG-IUS

The fitting of the LNG-IUS should ideally be done by the gynaecologist during the conservative surgery, or before discharge. If this is not possible, it can be fitted later by a GP or at a sexual health clinic, ideally within a month.

If LNG-IUS is fitted within seven days of the onset of menstruation or withdrawal bleeding it will provide immediate contraceptive cover, otherwise barrier methods must be used for 14 days. A check-up after 6 weeks is recommended by the Royal College of Obstetricians and Gynaecologists but is not mandatory for trial follow up. The LNG-IUS can remain *in situ* up to for 5 years and should be removed by a competent practitioner, with immediate replacement if desired.

5.2.3 Combined oral contraceptive

A 30mcg ethinylestradiol and 150 mcg levonorgestrel COCP, e.g. Microgynon 30/ Rigevidon is recommended for PRE-EMPT. The use of the COCP cyclically or continuously should be discussed and the decision should be left to the woman. To provide contraceptive cover, the first treatment cycle should commence on the first 5 days of menstruation, otherwise barrier methods should be used for 14 days. The woman should be given information about subsequent cycles, missed pills and special circumstances requiring additional contraception. Should there be significant reasons contrary to the use of 30mcg ethinylestradiol and 150 mcg levonorgestrel COCP an alternative should be chosen accordingly. This will not affect trial participation and will be captured in the dataset accordingly. COCP can be prescribed by the GP or at a sexual health clinic as per local practice and as appropriate.

5.3. Participant withdrawal and changes of status within trial

Informed consent is defined as the process of learning the key facts about a clinical trial before deciding whether or not to participate. It is a continuous and dynamic process and participants should be asked about their ongoing willingness to continue participation.

A participant can be withdrawn from the trial treatment if, in the opinion of the investigator or general practitioner, it is medically necessary to do so. A participant may also voluntarily withdraw participation in this study at any time. If a woman decides, after randomisation, she does not wish to remain in the allocated treatment group or wishes to conceive, she may do so. We will aim to document the reason for treatment change.

Clear distinction will be made between non-continuation of the allocated trial treatment, or initiation of a new, non-allocation treatment whilst allowing further follow-up, and refusal to allow any follow-up. If a patient explicitly withdraws consent to have any further data recorded their decision will be respected and recorded on the electronic data capture system. The original randomising gynaecologist will be informed of the withdrawal and no further data will be collected for that patient.

Types of withdrawal as defined are:

- The participant would like to withdraw from trial treatment, but is willing to be followed up in accordance with the schedule of assessments and if applicable using any central UK NHS bodies for long term outcomes (i.e. the participant has agreed that data can be collected and used in the trial analysis)
- The participant would like to withdraw from trial treatment and does not wish to attend trial visits in accordance with the schedule of assessments but is willing to be followed up at standard clinic visits and if applicable using any central UK NHS bodies for long term outcomes (i.e. the participant has agreed that data can be collected at standard clinic visits and used in the trial analysis, including data collected as part of long term outcomes)
- The participant would like to withdraw from trial treatment and is not willing to be followed up in any way for the purposes of the trial and for no further data to be collected (i.e. only data collected prior to the withdrawal can be used in the trial analysis)

or

- The participant wishes to withdraw completely (i.e. from trial treatment and all follow up) and is not willing to have any of their data, including that already collected, to be used in any future trial analysis

The details of withdrawal (date, reason and type of withdrawal) should be clearly documented in the source data.

5.4. Compliance monitoring

In the DMPA and COCP groups, if a participant does not return for a scheduled visit or repeat prescription, where resupply is from the trial site, the PRE-EMPT Trial Office will not immediately be aware of this non-compliance. We will request that GPs inform the Trial Office, but often this can be overlooked.

Each follow-up questionnaire will include questions regarding current and previous treatment and women reporting information on any changes will be captured and entered onto the database. Compliance data will be collected from the participant reported questionnaires.

5.5. Excluded medications or interactions

Patients will be advised to inform their GP or any other clinician caring for them that they are participating in the PRE-EMPT trial, and may be taking long acting progestogens (LNG-IUS or DMPA). Participants will be given a small information card to carry with them, with PRE-EMPT trial contact information, to direct clinicians to information regarding potential drug interactions.

Withdrawal from trial treatment does not necessitate withdrawal from the PRE-EMPT study – see Section **Error! Reference source not found.** above.

5.6. Other management at discretion of local doctors

Apart from the trial treatments allocated at randomisation, all other aspects of patient management are entirely at the discretion of the local doctors. Patients are managed in whatever way appears best for them, with no other special treatments, no special investigations, and no extra follow-up visits.

6. SAFETY MONITORING PROCEDURES

The collection and reporting of Adverse Events (AEs) will be in accordance with the UK Policy Framework for Health and Social Care (2017) and the requirements of the Health Research Authority (HRA). Definitions of different types of AEs are listed in the table of abbreviations and definitions. The Investigator should document all AEs experienced by the trial participant in the source data and assess the seriousness and causality (relatedness) with reference to the Reference Safety Information (RSI).

All PRE-EMPT trial treatments are widely used for treatment of endometriosis, and for long term use as contraceptives, and whilst rare, there have been associated serious adverse reactions

6.1. General Definitions

**Adverse Event**	AE	Any untoward medical occurrence in a participant or clinical trial subject administered a medicinal product and which does not necessarily have a causal relationship with this treatment.
**Adverse Reaction**	AR	All untoward and unintended responses to an IMP related to any dose administered.
**Serious Adverse Event**	SAE	Any untoward medical occurrence or effect that:<br>• Results in death is life-threatening*<br>• Requires hospitalisation or prolongation of existing hospitalisation<br>• Results in persistent or significant disability or incapacity<br>• Is a congenital anomaly/birth defect<br>• Or is otherwise considered medically significant by the Investigator**
**Serious Adverse Reaction**	SAR	An Adverse Reaction which also meets the definition of a Serious Adverse Event

**Unexpected Adverse Reaction**	UAR	An AR, the nature or severity of which is not consistent with the applicable product information (e.g. Investigator Brochure for an unapproved IMP or (compendium of) Summary of Product Characteristics (SPC) for a licensed product).<br>When the outcome of an AR is not consistent with the applicable product information the AR should be considered unexpected.
**Suspected Unexpected Serious Adverse Reaction**	SUSAR	A SAR that is unexpected i.e. the nature, or severity of the event is not consistent with the applicable product information.<br>A SUSAR should meet the definition of an AR, UAR and SAR.

Medical judgement should be exercised in deciding whether an adverse event/reaction should be classified as serious in other situations. Important adverse events/reactions that are not immediately life-threatening or do not result in death or hospitalisation, but may jeopardise the subject or may require intervention to prevent one of the other outcomes listed in the definition above, should also be considered serious.

Events NOT considered to be SAEs are hospitalisations for:

- routine treatment or monitoring of endometriosis, not associated with any deterioration in condition, including out-patient or GP appointments for repeat DMPA injections, LNG-IUS fitting, checking or removal, MRI or ultrasound scans.
- laparoscopic or open surgery for further treatment of endometriosis, nor hysterectomy.
- admission for acute exacerbation of endometriosis associated pain
- treatment, which was elective or pre-planned, for a pre-existing condition that is unrelated to endometriosis, and did not worsen
- admission to a hospital or other institution for general care, not associated with endometriosis.
- treatment on an emergency, outpatient basis for an event not fulfilling any of the definitions of serious given above and not resulting in hospital admission

Expected Adverse Reactions

Particular risk factors are associated with expected ARs in all of the PRE-EMPT trial drugs. Carefully history and assessment is needed to establish whether the participant is eligible for each of the potential PRE-EMPT trial drugs.

A summary of the expected ARs (LNG-IUS Mirena & Levosert, Depo Provera, Microgynon and Rigevidon COCP) is given in the Reference Safety Information (RSI – the information against which SAEs will be categorised). Investigators will be provided with any updates to the RSI, which should be filed in the ISF by the local research team.

6.2. Reporting AEs

PRE-EMPT AE reporting is conducted primarily by the participant. This is captured in the routine follow up questionnaires received during follow up.
Participants will be instructed to contact the clinical research team at any time after randomisation to join the trial if they have an event that requires hospitalisation, or an event that results in persistent or significant disability or incapacity.
Any reference to admissions in the questionnaires will be queried for their potential to be AE or SAE with the local site.

When an AE/SAE occurs, it is the responsibility of the Investigator to review all documentation (e.g. hospital notes and diagnostic reports) related to the event. If the AE meets the criteria of serious, an SAE form must be completed by the Investigator.
The SAE form collects information on the event and this includes the type of event, onset date, the Investigator assessment of severity and causality, date of resolution and the outcome.

The treatments in PRE-EMPT are generally well tolerated. Full details of contraindications and side effects that have been reported following administration of the IMPs can be found in the reference safety information. These events will be recorded in the case report form (CRF), but not on the AE log, unless they meet seriousness criteria. Expected AEs which are 'uncommon', 'rare' or where the frequency is 'unknown', will be recorded on the AE log and reported as an SAE if seriousness criteria is met.

Participants will be asked about hospital admissions, GP visits and any pregnancies on their postal follow-up assessments. Questions regarding mood and general health are captured in the booklet questionnaire. Participants will also be asked if they have changed treatments since randomisation. If there is any doubt as to whether a clinical observation is an AE, the event will be recorded.

6.3. Reporting SAEs

The trial follow up consists of participant reported questionnaires and no actual follow visits take place. Whilst the treatments in PRE-EMPT are long established, have tolerable side effect profiles and repeat supply is given through local NHS practice, there is still a requirement to monitor safety.

Participant questionnaires are reviewed on receipt in the trial office, for any occurrence or description of admissions to hospital occurring since the last follow up time point.

Where admissions or potential (serious) adverse events have taken place the trial office will contact the randomising site to conduct a cross check of the medical notes and to confirm the need to report a SAE or AE.

Once confirmed all SAEs must be recorded on the SAE Form (Serious Adverse Event) and faxed or emailed to the BCTU on 0121 415 9136 immediately and within 24 hours of the PRE-EMPT PI/research nurse or midwife confirming awareness of the event. The Principal Investigator (or medically qualified delegate) has to assess and assign seriousness, causality and severity to the SARs/SAEs before reporting.

In addition all SAEs and SARs must be recorded in the participants' medical records.

For each SAE, the following information will be collected:

- full details in medical terms with a diagnosis, if possible
- its duration (start and end dates; times, if applicable)
- action taken
- outcome
- causality, in the opinion of the investigator* (refer to RSI)

AEs defined as serious and which require reporting as an SAE should be reported on an SAE Form. Relatedness and severity of the SAE will be assessed by the Principal Investigator (or medically qualified delegate). The following categories will be used to define the relatedness (causality) of the SAE:

<table>
<tr><th>Category</th><th>Definition</th><th>Causality</th></tr>
<tr><td>Definitely</td><td>There is clear evidence to suggest a causal relationship, and other possible contributing factors can be ruled out</td><td rowspan="3">Related</td></tr>
<tr><td>Probably</td><td>There is evidence to suggest a causal relationship, and the influence of other factors is unlikely</td></tr>
<tr><td>Possibly</td><td>There is some evidence to suggest a causal relationship, however, the influence of other factors may have contributed to the event (e.g. the patient's clinical condition, other concomitant events or medication)</td></tr>
<tr><td>Not related</td><td>There is no evidence of any causal relationship</td><td>Unrelated</td></tr>
</table>

This will then be reported to the Sponsor within 24 hours of the CI or nominated individual being made aware of the incident. The CI or nominated individual will undertake a review of the causality assessment, and will perform the expectedness assessment. The CI will not overrule the causality, seriousness or severity assessment given by the local PI. If the CI disagrees with the local investigator's assessment, further clarification and discussion should take place to reach a consensus. If a consensus cannot be reached, both the opinion of the local investigator and the CI should be provided in the report to the MHRA and the NHS Research Ethics Committee (REC).

The local PI and others responsible for patient care should institute any supplementary investigations of SAEs based on their clinical judgement of the likely causative factors and

provide further follow-up information as soon as available. If a participant dies, any post-mortem findings must be provided to the BCTU. The BCTU will report all deaths to the Sponsor and DMEC for continuous safety review.

SAEs must be followed up at least until the final outcome is determined, even if it implies that the follow-up continues after the patient finishes the planned period of follow-up.

The BCTU will report all SAEs to the DMEC at routine DMEC meetings. The DMEC will view data presented by treatment group. BCTU will also report all SAEs to the main research ethics committee (REC) and the Medicines and Healthcare products Regulatory Authority (MHRA) annually, and to the Trial Steering Committee. Local Investigators are responsible for reporting SAEs to their host institution, according to local regulations, but they do not need to inform MHRA or main REC as this will be done by the BCTU/ Sponsor as detailed above.

6.4. Reporting SUSARs

SAEs categorised by the PI and/or CI as **both** suspected to be related to the trial drug **and** categorised as unexpected by the CI are SUSARs, and are subject to expedited reporting.

The BCTU will report all SUSARs to the Sponsor, MHRA and the main REC. If the SUSAR resulted in death or was life-threatening this will be done within 7 days of the initial report being received, or within 15 days for any other SUSAR.

If information is incomplete at the time of initial reporting, or the event is ongoing, the BCTU will request follow-up information, including information for categorisation of causality, from the local PI and will send the follow-up information to the MHRA and main REC within an additional 8 days for fatal or life-threatening SUSARs and as soon as possible for any other events.

6.5. Notification of deaths

All deaths will be reported to the BCTU on the SAE Form irrespective of whether the death is related to the trial treatment, or an unrelated event. If a participant dies, any post-mortem findings must be provided to the BCTU with the SAE form. The BCTU will report all deaths to the Sponsor and DMEC for continuous safety review.

6.6. Monitoring pregnancies for potential Serious Adverse Events

The Pre-Empt trial is using contraceptives for the treatment of endometriosis within a childbearing population, pregnancy and child birth is expected.

If a pregnancy notification is received, but the woman has stopped their trial treatment the pregnancy will not be followed up in an expedited manner. If a notification of pregnancy is received while the woman is currently on trial treatment, this will be followed up to confirm congenital anomaly or birth defect in line with the SAE definitions detailed above. Notifications of pregnancy are self-reported by the women, within the participant reported follow up questionnaires. The trial office makes checks on these data for pregnancies occurring within that follow up period, they are routinely followed up to confirm presence or absence of congenital abnormality or birth defects with the local Principal Investigator (or medically qualified delegate) and will be reported as serious adverse events.

6.7. Pharmacovigilance responsibilities

Local Principal Investigator (or medically qualified delegate).

- Medical judgement in assigning seriousness, severity and causality to SAEs.
- To fax SAE forms to BCTU within 24 hours of becoming aware, and to provide further follow-up information as soon as available.
- To report SAEs to local committees if required, in line with local arrangements.
- To sign an Investigator's Agreement accepting these responsibilities.

Chief Investigator (or nominated individual in CIs absence):

- To review causality and perform expectedness assessment on all SAEs
- To review the RSI at least annually and where there have been changes which would affect the expectedness assessment, ensure this is submitted as a substantial amendment

Birmingham Clinical Trials Unit:

Once the Investigator becomes aware that an SAE has occurred in a study participant, the information should be reported to BCTU via Fax on 0121 415 9136 or email pre-empt@trials.bham.ac.uk **immediately or within 24 hours**. If the Investigator does not have all information regarding an SAE, they should not wait for this additional information before notifying BCTU. The SAE report form can be updated when the additional information is received.

The SAE report will provide an assessment of causality and expectedness at the time of the initial report to BCTU according to sections 6.3, Reporting SAE's

The SAE form will be transmitted by email to pharmaco@abdn.ac.uk in a pdf format.

Where missing information has not been sent to BCTU after an initial report, BCTU will contact the investigator and request the missing information.

All reports faxed to BCTU and any follow up information will be retained by the co-ordinator in the Investigator Site File (ISF). Details received by BCTU will be passed on to BCTU in their capacity as Coordinating Centre.

Allocate each SAE form with a unique reference number and update and return the SAE form (containing the completed unique reference number) the site as proof of receipt within 1 working day

- In event of a SUSAR - report a minimal data set of all individual events categorised as a fatal or life threatening SUSAR to the (MHRA), main REC and the TMG within 7 days and follow up as outlined in section 6.4
- Notify the MHRA, main REC immediately if a significant safety issue is identified during the course of the trial.
- Report details of all SUSARs and any other safety issue which arises during the course of the trial to PIs.
- To prepare annual safety reports to the REC, TSC and MHRA. These will be submitted by the BCTU following approval from the sponsor.

- To prepare SAE safety reports for the DMC following a timetable agreed by the DMC prior to trial commencement, or as requested by the DMC
- To report all fatal SAEs to the DMC for continuous safety review

Co-Sponsors:

- To maintain oversight of safety and morbidity
- To review causality and expected nature of SAEs
- To review all events assessed as SUSARs in the opinion of the local PI and/or the CI. Local PI/CI assessment will not be overruled, but the Sponsors may wish to add comments prior to reporting to the MHRA
- To confirm continued sponsorship of the study

Trial Steering Committee (TSC):

- To provide independent supervision of the scientific and ethical conduct of the trial on behalf of the Trial Sponsor and funding bodies.
- To review data, patient compliance, completion rates, adverse events (during treatment and up to end of follow-up).
- To receive and consider any recommendations from the DMEC on protocol modifications.

Data Monitoring & Ethics Committee (DMEC):

- To review (initially at approximately 6-monthly intervals) overall safety and morbidity data to identify safety issues which may not be apparent on an individual case basis
- To recommend to the TSC whether the trial should continue unchanged, continue with protocol modifications, or stop.

7. FOLLOW-UP AND OUTCOME MEASURES

7.1. Primary outcome measure

The primary outcome is the recurrence of symptoms as evaluated by the pain domain of the EHP-30 questionnaire at 36 months post-randomisation. The EHP-30 is a disease specific questionnaire to measure the health status of women with endometriosis. It demonstrates good reliability, validity, acceptability and responsiveness(32), with low floor and ceiling effects for the core questions(33). EHP-30 contains 30 core items with 5 scales, and six modular parts of 23 questions which are dependent on the woman's circumstances e.g. impact on work, sexual activity and fertility. It will be assessed prior to randomisation and at 6, 12, 24 and 36 months.

7.2. Secondary outcome measures

7.2.1 Other aspects of endometriosis

The remaining four core domains of the EHP-30 (control, emotional aspects, social support and self-image) and the six modular domains (work, relationship with family, sexual intercourse, medical professions, treatment, infertility), where completed, will be compared between groups.

Pain scores will be collected using visual analogue scales. This involves use of a 10 cm line on a piece of paper representing a continuum of the participants' opinion of the degree of pain. It is explained to the participant that the one extreme of the line represents “no pain at all” while the other represents “as much pain as she can possibly imagine”. The woman rates the degree of pain by placing a mark on the line and 0-100 scale values are obtained by measuring the distance from zero to that mark. The recall period will be the last 4 weeks, to align with the EHP-30. The PRE-EMPT study will collect data on three sources of pain using VAS scores: dysmenorrhoea, dyspareunia, and other pelvic pain.

Fatigue, as measured by Fatigue Severity Score (FSS)(34), will also be assessed as it is an important consequence of endometriosis.

In response to comments from our patient/public (PPI) collaborators, we will ask women if they are using dietary supplements, following a gluten free or otherwise restrictive diet, or are using complementary therapies such as acupuncture. Although none of these interventions are evidence based, many women pursue them and place high value on their effectiveness.

7.2.2 Generic quality of life

Generic health-related quality of life (HRQOL) will be assessed using the EuroQol EQ-5D-5L instrument, and used to calculate utilities(35). PRE-EMPT will also collect data on women’s capabilities, as measure of wellbeing using the 5 question ICECAP-A instrument(36). It is advocated that the ICECAP measure provides a broader measure for economic evaluation. The measure incorporates five dimensions (each with four levels): attachment, security, enjoyment, role and control, and preference-based values for these dimensions (as required for economic outcome measures). It is based on Sen’s capability approach, and is considered an alternative to standard outcome valuation instruments such as the EQ-5D and SF-36 (or its adapted SF-6D form). Unlike these standard instruments, which single out health, the capability approach focuses on overall utility, which is likely to be relevant in the context of endometriosis, where instruments that focus on health outcomes alone may have limitations.

7.2.3 Compliance, withdrawal and further treatments

- Extent and success of initial conservative surgery.
- Further diagnostic and therapeutic surgery for endometriosis and use of analgesia (as a proxy for recurrence)
- Discontinuation rates of randomised treatment, with reasons for change, serious adverse events

7.2.4 Resource usage

- Cost per QALY and cost per case of symptoms avoided (where recurrence of pain is likely to be the driving symptom).

7.2.5 Patient experience

- Issues identified as important by the participants regarding their treatment and its impact on their lives, obtained from semi-structured interviews.

7.3. Format and timing of trial assessments

Women who agree to enter the study will complete a baseline participant booklet before randomisation. Booklets will consist of the disease specific and generic quality of life questionnaires, pain scores and resource use questions. Participants will then be followed

up for a period of three years (the primary outcome time point). Where consent has been provided text messages will be sent to each participant (where possible) to advise them their follow-up assessment is due and to remind them to complete and return as soon as possible. Over this period the outcomes will be collected according to Table 1 below. Data collection will be follow-up booklets sent to the woman's home address, to be completed and returned by freepost envelopes. A £10 gift voucher shall be included alongside the questionnaires to act as reimbursement for any inconvenience study participation may have caused them. For long term outstanding follow up questionnaires completed by post or phone a £25 voucher will be offered as a further compensation for any inconvenience study participation may have caused them (provided on return of completed questionnaire). Women will be asked to contact their local research teams to report any adverse events or pregnancies. Local research teams will be made aware of any pregnancies and adverse events through patients notes alerts as per local practices.

On receipt of the follow up questionnaires the trial team will review the completed data and raise any potential adverse events or pregnancy notifications to the local site teams who will then investigate the need to expedite reporting of and serious adverse events or pregnancies.

The trial team will maintain regular contact with local research teams to ensure they are kept updated on the progress of the trial and to encourage them to help with the return of data. This will include the circulation of return rate data via email mailshots and newsletters.

In the event of posted follow up questionnaires not being returned by participants for a period of over 2 months, local research teams will be asked to contact participants directly by telephone. Approximately once a month the trial team will provide local research teams with the trial ID numbers of participants who have overdue follow-up questionnaires. The local research team will ask participants to provide the required trial information over the phone. In these cases a shortened version of the questionnaire will be used that prioritises the data needed to fulfil the primary outcome. Once the telephone questionnaire has been completed it will be returned to the trials team and on receipt reviewed in the same way as the full follow up questionnaires. The full follow up questionnaire remains the preferred method of data collection.

Repeat laparoscopies, which will incur costs and can provoke the formation of intra-abdominal adhesions(37), or subsequent ultrasound investigations will not be mandated, but will be noted as resources used.

Table 1 Outcome assessment for PRE-EMPT

Timepoint<br><br>Outcome measure	Prior to randomisation	Prior to discharge following surgery	6 months	1 year	2 year	3year
**EHP-30**	X		X	X	X	X
**FSS**	X		X	X	X	X
**EQ-5D and ICECAP**	X		X	X	X	X
**Pregnancy**			**X**			

**Menstrual regularity**	X		X	X	X	X
**Serious adverse events**				As reported by clinician		
**Surgical procedure**		As reported by clinician				
**Resource usage (participant)**	X		X	X	X	**X**
**Repeat surgery**				As reported by clinician		
**Qualitative interviews**				X		X

*X as reported by participant

7.4. Data management and validation

Data for the trial is collected from hospitals initially for baseline data, but primary outcome data and majority of secondary outcome data is reported by participants within their questionnaire booklets, completed within the follow up schedule above (table 1.).
Data for the purpose of assessing the efficacy and safety within the PRE-EMPT trial will be collected from the participating sites by the clinical team responsible for the women`s care on a data collection (case report) forms (CRF). Data required for the primary and the majority of secondary outcomes are objective measures which are routinely collected for clinical purposes and will be transcribed from woman's medical records, with the exceptions of the information obtained at laparoscopy which will be directly recorded onto the relevant CRF and recorded in the patients notes. The respective data from the woman's medical notes and laparoscopy information constitute source data.
Paper forms will be used to confirm eligibility (prior to computer/telephone randomisation), to document informed consent and to collect data during laparoscopy. Information from the randomisation form, will be entered onto the database or via telephone by those with on-line access. It can be completed in paper form in the first instance but needs to be entered into the secure online PRE-EMPT database by the designated PRE-EMPT research nurse / midwife. The CRF`s then need to be photocopied and sent to the PRE-EMPT office to be entered onto the database then filed with the randomisation and consent form in the site file.
The participating sites will collect the woman's NHS and hospital number (where applicable) and both may be used in the process of collecting missing data. The Trial Office will only use the unique identifier for the participant for the purpose of data management.
The clinical personnel involved will be allocated personal usernames and passwords that will only allow access to forms for the trial participants who are being treated at their site. Data validation is built into the online database. Range, date and logic checks are performed at the point of data entry. Email reminders will be sent to the research nurses / midwives for missing data forms, missing data or data inconsistencies. This will be requested on a Data clarification form.

For each questionnaire there is a time limit from when the data are considered valid i.e. for the 6-month questionnaire data received up to 6 months after the 6-month questionnaire is due is considered valid. For the 12-month and 24-month form the tolerance of valid return is up to 12 months after the forms are due. We will not apply a tolerance validation to the 36 month form, allowing for later returned participant questionnaires (postal or telephone) to remain valid and contribute to the trial.

Site research nurses and PIs will maintain regular contact with the participants to ensure retention within the trial, capture any changes of address or participant relocation (that can be relayed to the trial team) and encourage return of the postal follow up questionnaires.

Two weeks before the follow up time point occurs the questionnaire (and voucher) is sent to the participant. Following this two further reminders are sent to the participants, two weeks apart each, with a final reminder at six weeks.

In addition to the postal reminders, where the two and three year follow up questionnaires are concerned; the local PI and research team will be contacted with shortened outcome questionnaire to collect from the participant over the phone.

The PI or appropriately delegated member of the research team will then complete the questionnaire recording the participant's answers, signing and dating the form and returning to the trial office for data entry.

If this approach is not successful the clinical and health care professionals within the TMG will attempt to contact the participants by telephone to complete the shortened outcome questionnaire following the process described above.

If pregnancy or adverse events are mentioned during this telephone questionnaire, a member of the research team will complete the appropriate forms accordingly.

7.5. Retention strategies

Ensuring that the participants are engaged and complete their follow up questionnaires is imperative to PRE-EMPT. Therefore the trial uses a voucher to offer to compensate participants for completing the questionnaires. The vouchers are sent out at the time of the initial postal questionnaire. For long term outstanding follow up questionnaires completed by post or phone a further £25 voucher will be provided on completion of the questionnaire.

In addition newsletters to participants, sent out six monthly, will be created by the trial office and will aim to keep the participants informed of trial developments and progress, and aim to galvanise their timely return of the follow questionnaires.

7.6. Long Term Storage of Data

Storage will be authorised by University of Aberdeen Sponsor following submission of the end of trial report.
Principal Investigators are responsible for the secure archiving of essential trial documents for their site, according to the local policy at that site. All essential documents will be archived for a minimum of 25 years after completion of trial.

Data validation is built into the online database, so that range, date and logic checks are performed at the point of data entry. Only once all required data is entered and validated will the participant's record be categorised as complete and valid – until then, email reminders will be sent to the research midwife for missing data or data inconsistencies.

7.7. Long-term follow-up

Although beyond the scope of the PRE-EMPT protocol at commencement of the trial, the intention will be to follow-up all participants at 5 and 10 year, should further funding become available.

7.8. Definition of the End of Trial

The end of the PRE-EMPT trial will be defined as twelve months after the final participant recruited reaches the 3 year follow-up time-point. Due to the long-term nature of some of the interventions, participants may remain on treatment beyond the end of the trial, and will be cared for by the general practitioner as they would outside of the trial. Any extension to the trial for long-term follow-up would be considered observational.

8. ACCRUAL AND ANALYSIS

8.1. Sample size

The sample size is designed to give 90% power to detect an 8 point difference on the EHP-30 pain domain in our main comparison and will also give good power (80%) to detect a 10 point difference in the two sub-comparisons. The estimate of standard deviation is 19 (from pooled baseline data in the internal pilot study) with 95% certainty that this is between 16 and 22. To err on the side of caution we have used 22 in the calculations below.

To detect an eight point difference in the main comparison with 90% power (p=0.05), assuming the SD is 22, will require 160 participants per group, 320 in total. Eight points is equivalent to 0.36 SD, which can be considered half-way between a small (0.2 SD) to moderate (0.5 SD) effect size(38).

Given our internal pilot data suggests a roughly even split between those with a preference for LNG-IUS and DMPA this would mean we will have approximately 160 women in each of our sub-comparisons. This would be enough participants to detect a 10 point difference with 80% power (p=0.05). In addition, we should be able to collect a small amount of data on the comparison between LNG-IUS v DMPA (in those were happy to be randomly allocated a LARC) but we are not anticipating enough patients in this comparison to be able to draw substantial conclusions.

To account for any loss to follow-up – which we are assuming to be 20% - we have inflated the sample size to 200 per group, 400 in total (and hence an estimated 200 in total in each sub-comparison).

8.2. Statistical Analysis

A separate Statistical Analysis Plan (SAP) will be developed to provide a detailed description of the planned statistical analyses and agreed with the Data Monitoring Committee before the first interim analysis.

The primary comparison will be of LARC (LNG-IUS or DMPA) compared with COCP. Separate analyses of LARC versus COCP in those who chose to be treated with LNG-IUS/DMPA will also be undertaken, but this will be considered of secondary importance. Participants will be considered in the groups to which they were allocated irrespective of compliance with treatment (intention-to-treat). Estimates of differences between groups will be reported with 95% confidence intervals and associated p-values.

8.2.1 Primary outcome analysis

For the primary outcome measure - pain domain from the EHP-30 at three years - a linear regression model will be used to analyse the responses, including a variable for each treatment group, baseline score and the minimisation variables (see section 4.1.2) as covariates. Responses at earlier time points will be considered in a similar fashion but as secondary analyses. Confidence intervals for the estimates between the groups will be calculated using standard errors taken from the linear model. Statistical significance will be assessed by F-test.

8.2.2 Secondary outcomes analysis

Data from other domains of the EHP-30 and the other quality of life measures (EQ-5D-5L, ICECAP-A, FSS, visual analogue pain scores) will be analysed in a similar fashion as to the primary outcome. Further exploratory analysis using multilevel repeated measures models will also be used to examine differences over all time-points (this will also be undertaken for the primary outcome). A group by time interaction parameter will be included model to examine if there is any converging or diverging effect over time. Other outcome measures will be analysed using standard methods (relative risks, log-rank tests).

8.2.3 Missing data

In the first instance analysis will be completed on received data, with every effort made to follow up participants even after protocol violation to minimise any potential bias. Sensitivity analysis of the primary outcome measure including imputed values for missing responses will be carried out to determine the robustness of the results obtained. Methods based on multiple imputation will be used.

8.2.4 Sensitivity analysis

Sensitivity analysis will also be performed excluding women who are no longer taking their allocated treatment. We will also explore if converging scores are attributable to cross-over by including a parameter for cross-over and examining its interaction with group and time in the multilevel model.

8.2.5 Subgroup analysis

Consultation with gynaecologists suggests that treatment effects may vary for greater severity of endometriosis and could be age dependent. Therefore, subgroup analyses will be limited to those variables listed in section 4.1.1. This will be carried out by examining subgroup by treatment interaction variables in the linear model.

8.2.6 Interim analysis

During the full study, an interim report including the analysis of major endpoints will be provided in strict confidence to a Data Monitoring and Ethics Committee (DMEC) at intervals of at least 12 months, or as to a timetable agreed by the DMEC prior to study commencement. Formal stopping rules will not be applied, the DMEC will be asked to use pragmatic criteria for any potential early stopping or modifying of the study (see section 10.2 for further details on trial data monitoring).

8.2.7 Final analysis

The final, primary analysis will occur once all patients have completed once all patients have reached three years follow-up.

9. ECONOMIC EVALUATION

9.1.1 Pre-trial model based economic evaluation

A decision analytic model based on the alternative treatment pathways outlined in the trial design will be constructed and populated from a review of the available evidence on resource use, associated costs, effectiveness of interventions and the HRQOL for the resulting health states. This collated evidence will be used to estimate a baseline decision model which will allow important elements of resource use, such as frequency and dose of medical treatment, or exact surgical intervention, related to this, costs and issues and gaps relating to either the interventions or quality of life can be identified. The important elements of resources use identified as main cost drivers are defined as those which are highlighted to have significant cost implications e.g. further surgical interventions or adverse events associated with any of the medical treatments. Some costs identified as important may be further investigated in a primary cost data collection exercise carried out alongside the trial. The approach to constructing the model at the beginning of the trial, during the pilot phase, will allow important issues of model structure to be anticipated.

An economic evaluation alongside a trial cannot account for all differences between each individual women, at each point in time and a model is used to group women into common health states and to represent repeated events where appropriate (medical treatment, surgical intervention, HRQOL etc.). However, the model typically will not illustrate every individual woman's consumption of particular resources within grouped health states and sometimes require generalisable assumptions to be made about the compliance with treatment, or the type of surgical intervention etc. The pre-trial model can test these assumptions to see their importance in changing the results. Significant differences can be further researched from within the trial (whilst it is in progress and still recruiting) to ensure there is appropriate supporting evidence for the full range of required assumptions.

9.1.2 Model based economic evaluation alongside the trial

If any of the interventions are found to be an effective approach in preventing the recurrence of endometriosis then it is likely that there will be important cost implications for the health care sector and potentially beyond. For example, any positive impact on woman's quality of life may mean she has fewer visits to her GP and health services, but may also means there are fewer interruptions to her daily routines, which may be within the home such as looking after children or outside the home in paid employment. Therefore all associated resource use and costs incurred by both the health service, women's private out of pocket costs and wider society need to be assessed in conjunction with measures of effectiveness.

The aim of the economic evaluation is to determine the additional costs and effectiveness of one hormonal treatment compared to another. Additionally, although the trial will not be collecting primary data on a 'no treatment option' – a 'no treatment' comparison can still be included in the model based analysis if sufficient data exist to make sensible assumption about the implications in terms of costs and resource use for women who receive no treatment. This could be included in a sensitivity analysis. But this will depend on whether existing data are sufficient make 'no treatment' a viable alternative in the analysis. The most appropriate type of analysis is a cost-effectiveness analysis based on the outcome of

cost per QALY and cost per case of symptom avoided (where recurrence of pain is likely to be the driving symptom). The utility values required to calculate QALYs will be obtained by administering the EuroQol EQ-5D-5L questionnaire at the time points described in section 7.3. Data on women's capabilities, using The ICECAP index of capability, will also be collected. The evaluation will consider costs incurred by the health service and society in the delivery of all three treatment pathways. Thus resource use information on costs or time off work incurred by women will also be collected in order that an evaluation from a wider societal perspective can also be undertaken.

9.1.3 Cost data collection

Data collection will be undertaken prospectively for all trial participants so that a stochastic cost analysis can be undertaken. The process of collecting resource use data will be undertaken separately from data collection on unit costs.

The main resource uses to be collected include the following:

- Treatment received at different time points along the treatment pathway including frequency and dose.
- Health service visits leading to changes in treatment
- Visits to the health service for any related condition
- Hospital admissions for scans and/or surgical interventions

Information on additional related primary or secondary care contacts will also be collected from women to ensure any resulting resource use from additional complications is recorded in the form of a questionnaire which will be administered at the same time points as the EQ-5D and ICECAP questionnaires. A validated patient resource use questionnaire will be used to collect patient data, on private travel for hospital appointment and time off work for example, such a questionnaire has already been developed and used in other trials (OPT www.opt.bham.ac.uk). Unit costs will be obtained and attached to resource items in order that a cost can be calculated for each trial patient. Published sources for these costs will include Unit Costs of Health and Social Care and NHS Reference costs.

9.1.4 Analyses

The pre-trial model structure will be refined to accommodate and reflect the known pathways that are followed by women in the trial but may include additional known pathways, for example 'no treatment' as part of an additional sensitivity analysis . The model will consider treatment over total disease duration and will include further treatments provided in the longer term. An individual sampling model (such as a Markov model) will be used since individual patients in the model can, for modelling purposes, be regarded as independent. The model-based analysis will draw upon follow-up data collated at the end of the 3 year study period and if deemed appropriate will make use of published data and assumptions to predict costs and benefits into the longer-term.

The data available will be patient-specific resource use and costs. Given the skewness inherent in most cost data and the concern of economic analyses with mean costs, we shall use a bootstrapping approach in order to calculate confidence intervals around the difference in mean costs. An incremental economic analysis will be undertaken. The base-case analysis will be framed in terms of cost-consequences, reporting data in a disaggregated manner on the incremental cost, the broad range of consequences including data on endometriosis symptoms, in particular pain, quality of life, etc. If this identifies a situation of dominance then further analysis will not be required. If no dominance is found,

cost effectiveness (i.e. cost per change in symptom score) and cost-utility analyses (i.e. cost per QALY gained) will be undertaken. The EQ-5D-5L will be used to derive utilities.

Using discounting, adjustments will be made to reflect this differential timing. The base-case analysis will follow Treasury recommendations for public sector projects: currently the recommendation is a rate of 3.5% for costs and benefits, although sensitivity analysis using different rates will be performed.

Results of all economic analyses will be presented using cost-effectiveness acceptability curves to reflect sampling variation and uncertainties in the appropriate threshold cost effectiveness value. Simple and probabilistic sensitivity analyses will be used to explore the robustness of these results to plausible variations in key assumptions and variations in the analytical methods used, and to consider the broader issue of the generalisability of the results.

10. DATA ACCESS AND QUALITY ASSURANCE

10.1. Confidentiality of personal data

Personal data and sensitive information required for the PRE-EMPT Trial will be collected directly from trial participants and hospital notes on data collection forms, coded with the participant's unique trial number and initials. Participants will be informed about the transfer of this information to the PRE-EMPT Trial Office at the BCTU and asked for their consent. The consent form will also be faxed to the PRE-EMPT Trial Office, as this is the sole document with identifiable details, again with consent from the participant. The data will be entered onto a secure computer database, directly via the internet using secure socket layer encryption technology or indirectly from paper forms by BCTU staff.

All personal information received in paper format for the trial will be held securely and treated as strictly confidential according to BCTU policies. All staff involved in the PRE-EMPT Trial (clinical, academic, BCTU) share the same duty of care to prevent unauthorised disclosure of personal information. No data that could be used to identify an individual will be published. Data will be stored on a secure server at BCTU under the provisions of the General Data Protection Regulation and/or applicable laws and regulations.

Personal data recorded on all documents will be regarded as strictly confidential and will be handled and stored in accordance with the General Data Protection Regulation 2016/679 and the Data Protection Act 2018.

10.2. Data Quality Assurance

10.2.1 Monitoring and Audit

Investigators and their host Trusts will be required to permit trial-related monitoring and audits to take place by a representative of the PRE-EMPT Trial team, and/or Sponsor representatives providing direct access to source data and documents as requested. Monitoring of **PRE-EMPT** will ensure compliance with GCP. A risk proportionate approach to the initiation, management and monitoring of **PRE-EMPT** will be adopted and outlined in the trial-specific risk assessment Trusts may also be subject to inspection by the Medicines and Healthcare Products Regulatory Agency and/ or by the Research and Development Manager of their own Trust and should do everything requested by the Chief Investigator in

order to prepare and contribute to any inspection or audit. Trial participants will be made aware of the possibility of external audit of data they provide in the participant information sheet.

10.2.2 Trial drug quality assurance

LNG-IUS, DMPA and COCP are not specifically licenced for endometriosis, but are very widely used contraceptives and licenced for other menstrual disorders. The method of dispensing the trial drugs is varied, with LNG-IUS being a one-off fitting, ideally at the hospital, whilst DMPA and COCP may be initiated in the hospital, GP practice or a local family planning clinic. Repeat prescriptions of the COCP will be via the GP, with prescriptions being dispensed at community pharmacies. None of the trial drugs are being modified or blinded in any way, and are prescribed as per their contraceptive indication. Therefore, the allocated interventions will be taken from normal, non-trial stock and the standard NHS labelling for dispensed medicines will apply. Participants will be provided with a card with relevant contact details and to identify their participation in PRE-EMPT. Product liability will rest with the holders of the manufacturing authorisations.

10.2.3 Statistical monitoring throughout the trial

The study will also adopt a centralised approach to monitoring data quality and compliance. A computer database will be constructed specifically for the trial data and will include range and logic checks to prevent erroneous data entry. Independent checking of data entry will be periodically undertaken on small sub-samples. The trial statistician will regularly check the balance of allocations by the stratification variables.

10.3. Independent Trial Steering Committee

The Trial Steering Committee (TSC) provides independent supervision for the trial, providing advice to the Chief and Co- Investigators and the Sponsor on all aspects of the trial and affording protection for patients by ensuring the trial is conducted according to Guidelines for Good Clinical Practice guidelines in Clinical Trials.

If the Chief and Co-Investigators are unable to resolve any concern satisfactorily, Principal Investigators, and all others associated with the study, may write through the Trial Office to the chairman of the TSC, drawing attention to any concerns they may have about the possibility of particular side-effects, or of particular categories of patient requiring special study, or about any other matters thought relevant.

The BCTU Trial office will forward TSC meeting minutes to the Sponsor and funding Body.

10.4. Data Monitoring and Ethics Committee

If one treatment really is substantially better or worse than any other with respect to the primary outcome, then this may become apparent before the target recruitment has been reached. Alternatively, new evidence might emerge from other sources that any one treatment is definitely more, or less, effective than any other. To protect against this, during the main period of recruitment to the study, interim analyses of the primary outcome and adverse events will be supplied, in strict confidence, to an independent Data Monitoring and Ethics Committee (DMEC) along with updates on results of other related studies, and any other analyses that the DMEC may request. The DMEC will advise the chair of the TSC if, in their view, any of the randomised comparisons in the trial have provided both (a) “proof

beyond reasonable doubt"* that for all, or for some, types of patient one particular treatment is definitely indicated or definitely contraindicated in terms of a net difference in the major endpoints, and (b) evidence that might reasonably be expected to influence the patient management of many clinicians who are already aware of the other main trial results. The TSC can then decide whether to close or modify any part of the trial. Unless this happens, however, the TMG, TSC, the investigators and all of the central administrative staff (except the statisticians who supply the confidential analyses) will remain unaware of the interim results.

The BCTU Trial office will forward open DMEC meeting minutes to the Sponsor and funding Body.

10.5. Long-term storage of data

After the end of the trial, the site files from each centre will be collected and incorporated into the trial master file held by the BCTU.

In line with the Medicines for Human Use (Clinical Trials) Regulations, once data collection is complete on all participants, all data will be stored for at least 15 years. This will allow adequate time for review and reappraisal, and in particular with the PRE-EMPT trial, form the basis for further follow-up research. Any queries or concerns about the data, conduct or conclusions of the trial can also be resolved in this time. Limited data on the participants and records of any adverse events may be kept for longer if recommended by an independent advisory board.

Trial data will be stored within the BCTU under controlled conditions for at least 3 years after closure. Long-term offsite data archiving facilities will be considered for storage after this time. The BCTU has standard processes for both hard copy and computer database legacy archiving.

10.5.1 Data Sharing

The PRE-EMPT Trial Management Committee will endeavour to make the complete dataset available to the scientific community to maximize the value of the data for research and for patient and public benefit. Such data must be shared in a timely and responsible manner, so it will not be available before the publication of the monograph required by the funding body. After this time, anonymised individual patient level data may be made available other researcher proposing statistically rigorous analyses that also adds recognisable value to the original dataset. Such research is often most fruitful when it is a collaboration between the new user and the original trial team, with the responsibilities and rights of all parties agreed at the outset. Data arising from PRE-EMPT will be properly curated throughout its life-cycle and released with the appropriate high-quality metadata. This is the responsibility of the PRE-EMPT Trial Office.

---

* Appropriate criteria of proof beyond reasonable doubt cannot be specified precisely, but a difference of at least $p<0.001$ (similar to a Haybittle-Peto stopping boundary) in an interim analysis of a major endpoint may be needed to justify halting, or modifying, the study prematurely. If this criterion were to be adopted, it would have the practical advantage that the exact number of interim analyses would be of little importance, so no fixed schedule is proposed.

11. ORGANISATION AND RESPONSIBILITIES

To ensure the smooth running of the trial and to minimise the overall procedural workload, it is proposed that each participating centre should designate individuals who would be chiefly responsible for local co-ordination of clinical and administrative aspects of the trial.

All investigators are responsible for ensuring that any research they undertake follows the agreed protocol, for helping care professionals to ensure that participants receive appropriate care while involved in research, for protecting the integrity and confidentiality of clinical and other records and data generated by the research, and for reporting any failures in these respects, adverse drug reactions and other events or suspected misconduct through the appropriate systems.

11.1. Centre eligibility

Patients will be recruited from the gynaecological, out-patient clinics of participating centres, fitting around their current service provision. Surgeons undertaking the conservative surgery prior to randomisation should have suitable experience and have still or video capture equipment to collect images of endometrial lesions during laparoscopy. Appropriate methods of investigation of endometriosis and standards for treatment and surgery, as defined by the Royal College of Obstetrics and Gynaecology Greentop Guideline No. 24 on the initial management of endometriosis, should be followed. Long term medical treatment may be delivered by the participants GP, as per current practice. Recruitment will be supported by dedicated research nurses, who will work with local gynaecology leads and liaise with regional research nurse co-ordinators.

11.2. Local co-ordinator at each centre

Each Centre should nominate a gynaecologist to act as the local Principal Investigator and bear responsibility for the conduct of research at their centre. Close collaboration between all clinical teams is particularly important in PRE-EMPT in order that patients for whom long-acting progestogens are options can be identified sufficiently early for entry. The responsibilities of the local PI will be to ensure that all medical and nursing staff involved in the care of women with endometriosis are well informed about the study and trained in trial procedures, including obtaining informed consent. The local PI should liaise with the Trial Coordinator on logistic and administrative matters connected with the trial.

11.3. Research nurses at each centre

Each participating centre should also designate at least one nurse as a PRE-EMPT research nurse. This person would be responsible for ensuring that all eligible patients are considered for the study, that women are provided with study information sheets, and have an opportunity to discuss the study if required. The nurse will be responsible for ensuring the baseline participant questionnaire is completed and for randomisation.

11.4. The PRE-EMPT Trial Office

The Trial Office at the University of Birmingham Clinical Trials Unit (BCTU) is responsible for providing all trial materials, including the trial folders containing printed materials and the update slides. These will be supplied to each collaborating centre, after relevant local research governance approval has been obtained. Additional supplies of any printed material can be obtained on request. The Trial Office also provides the central

randomisation service and is responsible for collection and checking of data (including reports of serious adverse events thought to be due to trial treatment), for reporting of serious and unexpected adverse events to the sponsor and/ or MHRA and for analyses. The Trial Office will help resolve any local problems that may be encountered in trial participation.

11.5. Research Governance

The conduct of the trial will be according to the Medicines for Human Use (Clinical Trials) Regulations 2004 and any subsequent amendments and the principles of the International Committee on Harmonisation Good Clinical Practice (GCP) Guidelines.

All centres will be required to sign an Investigator's Agreement, detailing their commitment to accrual, compliance, GCP, confidentiality and publication. Deviations from the agreement will be monitored and the TSC will decide whether any action needs to be taken, e.g. withdrawal of funding, suspension of centre.

The Trial Office will ensure researchers not employed by an NHS organisation hold an NHS honorary contract for that organisation.

11.6. Regulatory and Ethical Approval

11.6.1 Ethical and Trust Management Approval

The Trial has a favourable ethical opinion from North of Scotland Research Ethics Committee 1 (REC) approval, ethics application has been transferred to the East of Scotland Ethics Committee from 26th May 2014 determining that the trial design respects the rights, safety and wellbeing of the participants.

The Local Comprehensive Research Network in England, and equivalents in the devolved nations, will conduct governance checks and assess the facilities and resources needed to run the trial, in order to give host site permission. The Trial Office is able to help the local Principal Investigator in the process of the site specific assessment by completing much of site specific information section of the standard ethics and research governance application form as possible. The local Principal Investigator will be responsible for liaison with the Trust management with respect to locality issues and obtaining the necessary signatures at their Trust.

As soon as Trust approval has been obtained, the Trial Office will send a folder containing all trial materials to the local Principal Investigator. Potential trial participants can then start to be approached

11.6.2 Clinical Trial Authorisation

The Trial Office has obtained Clinical Trials Authorisation from the MHRA and has obtained a unique EudraCT number for the trial.

11.7. Funding and Cost implications

The research costs of the trial are funded by a grant from the NIHR Health Technology Assessment Programme awarded to the University of Aberdeen.

The trial has been designed to minimise extra 'NHS support' costs for participating hospitals, with no extra visits to hospital and no extra tests. Additional support costs associated with the trial, e.g. gaining consent etc., will be determined in negotiation with NHS Grampian, as the lead NHS organisation. These costs should be met by accessing the Trust's budget via the Local Comprehensive Research Network or equivalent in the devolved nations.

11.8. Indemnity

There are no special arrangements for compensation for non-negligent harm suffered by patients as a result of participating in the study. The study is not an industry-sponsored trial and so ABPI/ABHI guidelines on indemnity do not apply. The normal NHS indemnity liability arrangements for research detailed in HSG96(48) will operate in this case.

However, it should be stressed that in terms of negligent liability, NHS Trust hospitals have a duty of care to a patient being treated within their hospital, whether or not that patient is participating in a clinical trial. Apart from defective products, legal liability does not arise where there is non-negligent harm. NHS Trusts may not offer advance indemnities or take out commercial insurance for non-negligent harm.

11.9. Publication

A meeting will be held after the end of the study to allow discussion of the main results among the collaborators prior to publication. The success of the study depends entirely on the wholehearted collaboration of a large number of doctors, nurses and others. For this reason, chief credit for the main results will be given not to the committees or central organisers but to all those who have collaborated in the study. Centres will be permitted to publish data obtained from participants in the PRE-EMPT Trial that use trial outcome measures but do not relate to the trial randomised evaluation and hypothesis.

11.10. Ancillary studies

It is requested that any proposals for formal additional studies of the effects of the trial treatments on some patients (e.g. special investigations in selected hospitals) be referred to the Trial Management Committee for consideration. In general, it would be preferable for the trial to be kept as simple as possible, and add-on studies will need to be fully justified.

12. REFERENCES

1. Eltabbakh GH, Bower NA. Laparoscopic surgery in endometriosis. Minerva Ginecologica. 2008;60(4):323-30.
2. Nnoaham KE, Hummelshoj L, Webster P, D'Hooghe T, De Cicco Nardone F, De Cicco Nardone C, et al. Impact of endometriosis on quality of life and work productivity: A multicenter study across ten countries. Fertil Steril. 2011;96(2):366-73.
3. Eskenazi B, Warner ML. Epidemiology of endometriosis. Obstetrics and Gynecology Clinics of North America. 1997;24(2):235-58.
4. Simoens S, Dunselman G, Dirksen C, Hummelshoj L, Bokor A, Brandes I, et al. The burden of endometriosis: Costs and quality of life of women with endometriosis and treated in referral centres. Human Reproduction. 2012;27(5):1292-9.
5. Jones GL, Kennedy SH, Jenkinson C. Health-related quality of life measurement in women with common benign gynecologic conditions: A systematic review. American Journal of Obstetrics and Gynecology. 2002;187(2):501-11.
6. Garry R. The effectiveness of laparoscopic excision of endometriosis. Current Opinion in Obstetrics and Gynecology. 2004;16(4):299-303.
7. Jacobson TZ, Duffy-James MN, Barlow D, Koninckx PR, Garry R. Laparoscopic surgery for pelvic pain associated with endometriosis. Cochrane Database of Systematic Reviews. 2009;4(Art. No.: CD001300.).
8. Weir E, Mustard C, Cohen M, Kung R. Endometriosis: What is the risk of hospital admission, readmission, and major surgical intervention? Journal of Minimally Invasive Gynecology. 2005;12(6):486-93.
9. Valle RF, Sciarra JJ. Endometriosis: Treatment strategies. Ann NY Acad Sci. 2003;997:229-39.
10. Vercellini P, Crosignani PG, Fadini R, Radici E, Belloni C, Sismondi P. A gonadotrophin-releasing hormone agonist compared with expectant management after conservative surgery for symptomatic endometriosis. BrJ Obstet Gynaecol. 1999;106(7):672-7.
11. Morgante G, Ditto A, La Marca A, De Leo V. Low-dose danazol after combined surgical and medical therapy reduces the incidence of pelvic pain in women with moderate and severe endometriosis. Human Reproduction. 1999;14(9):2371-4.
12. Strowitzki T, Marr J, Gerlinger C, Faustmann T, Seitz C. Dienogest is as effective as leuprolide acetate in treating the painful symptoms of endometriosis: A 24-week, randomized, multicentre, open-label trial. Human Reproduction. 2010;25(3):633-41.
13. Guzick DS, Huang LS, Broadman BA, Nealon M, Hornstein MD. Randomized trial of leuprolide versus continuous oral contraceptives in the treatment of endometriosis-associated pelvic pain. Fertility and Sterility. 2011;95(5):1568-73.
14. Sesti F, Pietropolli A, Capozzolo T, Broccoli P, Pierangeli S, Bollea MR, et al. Hormonal suppression treatment or dietary therapy versus placebo in the control of painful symptoms after conservative surgery for endometriosis stage III-IV. A randomized comparative trial. Fertility & Sterility. 2007;88(6):1541-7.
15. Bayoglu Tekin Y, Dilbaz B, Altinbas SK, Dilbaz S. Postoperative medical treatment of chronic pelvic pain related to severe endometriosis: Levonorgestrel-releasing intrauterine system versus gonadotropin-releasing hormone analogue. Fertility and Sterility. 2011;95(2):492-6.
16. Petta CA, Ferriani RA, Abrao MS, Hassan D, Rosa e S, Podgaec S, et al. Randomized clinical trial of a levonorgestrel-releasing intrauterine system and a depot GnRH analogue for the treatment of chronic pelvic pain in women with endometriosis. Human Reproduction. 2005;20(7):1993-8.

17. Child TJ, Seang LT. Endometriosis aetiology, pathogenesis and treatment. Drugs. 2001;61(12):1735-50.
18. Daniels JP, Clark TJ, Bhattacharya S, editors. BSGE Survey of current post-operative hormonal treatment for Endometriosis. BSGE Annual Scientific Meeting; 2012.
19. Royal College of O, Gynaecologists. The Initial Management of Chronic Pelvic Pain. London: Royal College of Obstetricians and Gynaecologists, 2012.
20. Crosignani PGL. Subcutaneous depot medroxyprogesterone acetate versus leuprolide acetate in the treatment of endometriosis-associated pain. Human Reproduction. 2006;21(1):Jan.
21. Vercellini P, Frontino G, De GO, Aimi G, Zaina B, Crosignani PG. Comparison of a levonorgestrel-releasing intrauterine device versus expectant management after conservative surgery for symptomatic endometriosis: a pilot study. Fertility & Sterility. 2003;80(2):305-9.
22. Tanmahasamut P, Rattanachaiyanont M, Angsuwathana S, Techatraisak K, Indhavivadhana S, Leerasiri P. Postoperative levonorgestrel-releasing intrauterine system for pelvic endometriosis-related pain: a randomized controlled trial. Obstet Gynecol. 2012;119(3):519-26.
23. Furness S, Yap C, Farquhar C, Cheong YC. Pre and post-operative medical therapy for endometriosis surgery. Cochrane Database of Systematic Reviews. 2004;4(Art. No.: CD003678.).
24. Abou-Setta AM, Al-Inany HG, Farquhar CM. Levonorgestrel-releasing intrauterine device (LNG-IUD) for symptomatic endometriosis following surgery. Cochrane database of systematic reviews (Online). 2006(4).
25. Wong AYK, Tang LCH, Chin RKH. Levonorgestrel-releasing intrauterine system (Mirena ¶©) and Depot medroxyprogesterone acetate (Depoprovera) as long-term maintenance therapy for patients with moderate and severe endometriosis: A randomised controlled trial. Aus NZ J Obstet Gynaecol. 2010;50(3):273-9.
26. Novick G. Is there a bias against telephone interviews in qualitative research? Research in nursing & health. 2008;31(4):391-8.
27. Bryman A. Social research methods. Oxford; New York: Oxford University Press; 2008.
28. Wu L, Wu Q, Liu L. Oral contraceptive pills for endometriosis after conservative surgery: a systematic review and meta-analysis. Gynecological endocrinology : the official journal of the International Society of Gynecological Endocrinology. 2013;29(10):883-90.
29. Vercellini P, S DEM, Somigliana E, Buggio L, Frattaruolo MP, Fedele L. Long-term adjuvant therapy for the prevention of postoperative endometrioma recurrence: a systematic review and meta-analysis. Acta Obstet Gynecol Scand. 2013;92(1):8-16.
30. Vercellini P, Fedele L, Aimi G, Pietropaolo G, Consonni D, Crosignani PG. Association between endometriosis stage, lesion type, patient characteristics and severity of pelvic pain symptoms: a multivariate analysis of over 1000 patients. Hum Reprod. 2007;22(1):266-71.
31. Lidegaard O, Lokkegaard E, Svendsen AL, Agger C. Hormonal contraception and risk of venous thromboembolism: national follow-up study. BMJ: British Medical Journal. 2009;339.
32. Jones G, Jenkinson C, Kennedy S. Evaluating the responsiveness of the Endometriosis Health Profile Questionnaire: the EHP-30. QualLife Res. 2004;13(3):705-13.
33. Jones G, Jenkinson C, Taylor N, Mills A, Kennedy S. Measuring quality of life in women with endometriosis: tests of data quality, score reliability, response rate and scaling assumptions of the Endometriosis Health Profile Questionnaire. HumReprod. 2006;21(10):2686-93.
34. Krupp LB, LaRocca NG, Muir-Nash J, Steinberg AD. The fatigue severity scale. Application to patients with multiple sclerosis and systemic lupus erythematosus. Archives of Neurology. 1989;46(10):1121-3.

35. Williams A. Euroqol - A New Facility for the Measurement of Health-Related Quality-Of-Life. Health Policy. 1990;16(3):199-208.
36. Al-Janabi H, Flynn TN, Coast J. Development of a self-report measure of capability wellbeing for adults: the ICECAP-A. Quality of Life Research. 2012;21(1):167-76.
37. Somigliana E, Vigano P, Benaglia L, Busnelli A, Vercellini P, Fedele L. Adhesion prevention in endometriosis: a neglected critical challenge. Journal of Minimally Invasive Gynecology. 2012;19(4):415-21.
38. Cohen J. Statistical Power Analysis for the Behavioural Sciences. New York: Acedemic Press; 1977.

13. APPENDICES

APPENDIX 1: PRE-EMPT TRIAL SCHEMA

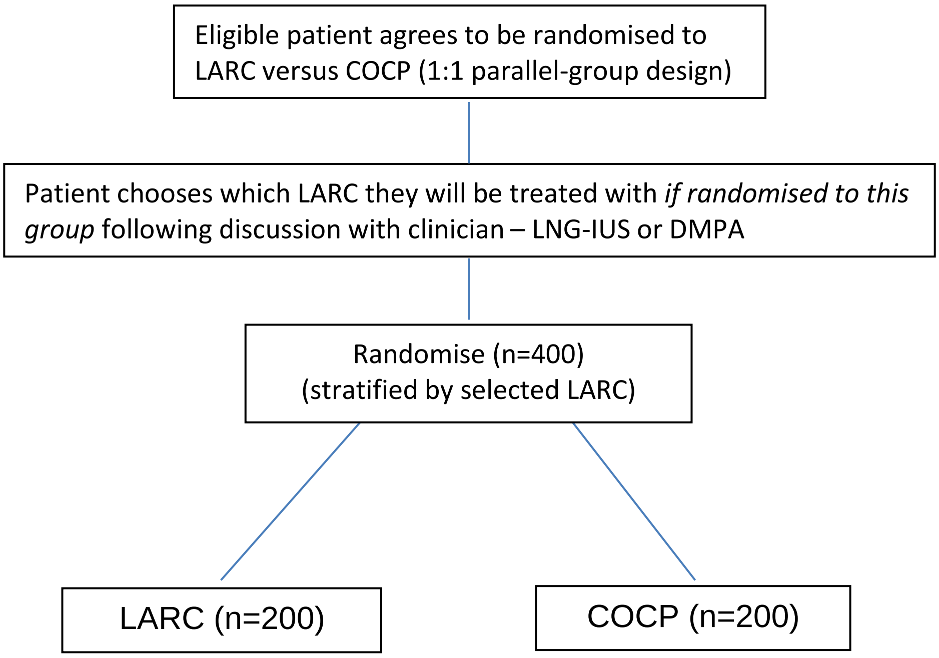

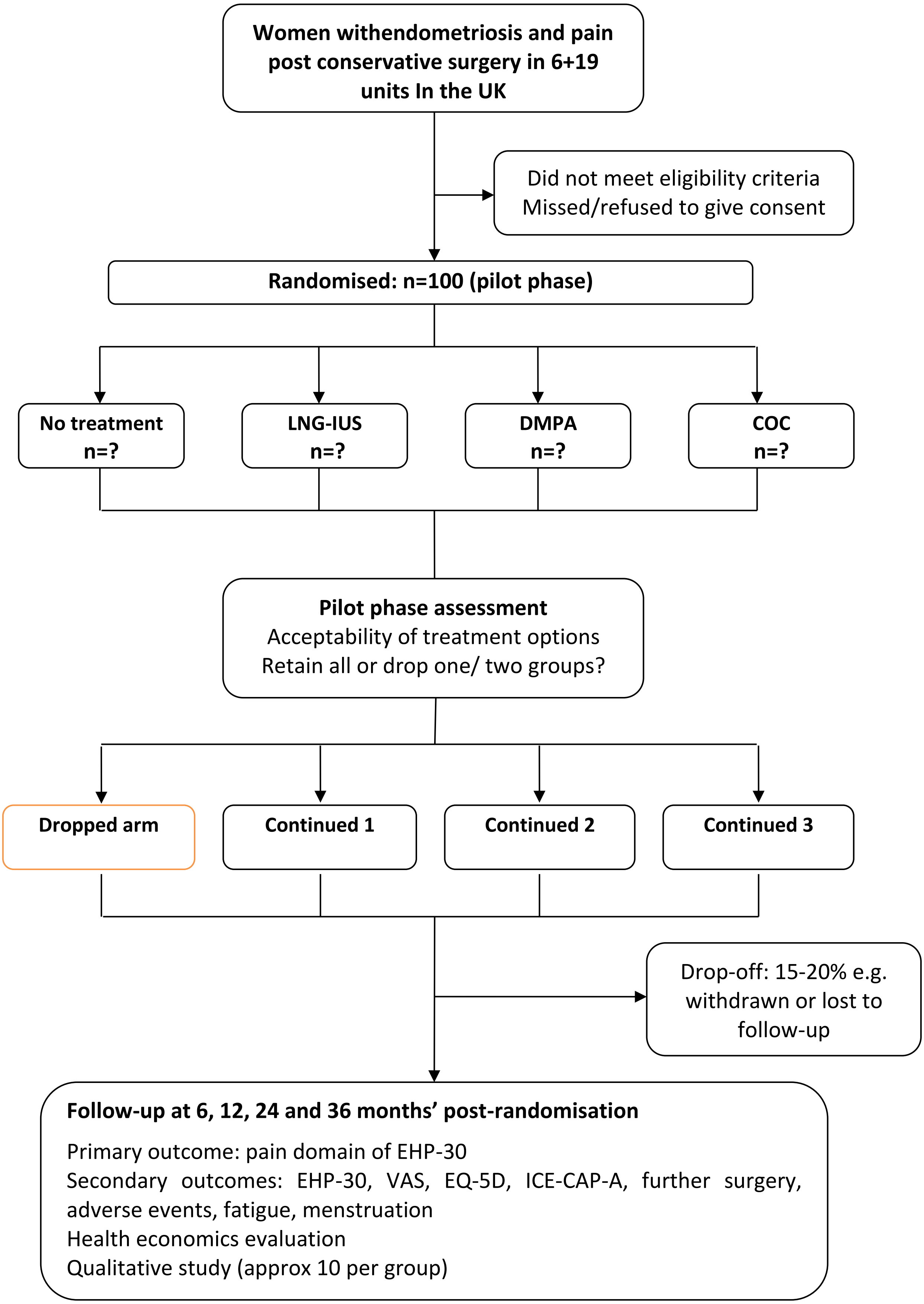
Women withendometriosis and pain post conservative surgery in 6+19 units In the UK
Did not meet eligibility criteria
Missed/refused to give consent
Randomised: n=100 (pilot phase)
No treatment n=?
LNG-IUS n=?
DMPA n=?
COC n=?
Pilot phase assessment
Acceptability of treatment options
Retain all or drop one/ two groups?
Dropped arm
Continued 1
Continued 2
Continued 3
Drop-off: 15-20% e.g. withdrawn or lost to follow-up
Follow-up at 6, 12, 24 and 36 months' post-randomisation
Primary outcome: pain domain of EHP-30
Secondary outcomes: EHP-30, VAS, EQ-5D, ICE-CAP-A, further surgery, adverse events, fatigue, menstruation
Health economics evaluation
Qualitative study (approx 10 per group)
