# Supplementary material for: Long acting progestogens versus combined oral contraceptive pill for preventing recurrence of endometriosis related pain: the PRE-EMPT pragmatic, parallel group, open label, randomised controlled trial
Source: BMJ. 2024 May 15;385:e079006. doi: 10.1136/bmj-2023-079006 (PMC11094611; doi:10.1136/bmj-2023-079006)
Supplement: Supplementary file 2 — Web appendix: Supplementary material 2—statistical analysis plan [file cook079006.ww2.pdf]

A randomised trial of Preventing Recurrence of Endometriosis by Means of long acting Progestogen Therapy: The PRE-EMPT Trial

Trial Registration: ISRCTN 97865475

Statistical Analysis Plan

SAP Version Number	Protocol Version Number
2.0	7.0

Name of Author:	Versha Cheed	Role:	Trial Statistician	Affiliation:	BCTU<br>University of Birmingham
Signature of Author:		Date:	14/11/2022		
Name of Chief Investigator:	Professor Kevin Cooper	Role:	Chief Investigator	Affiliation:	Aberdeen Royal Infirmary and University
Signature of Chief Investigator:		Date:	14/11/2022		
**This Statistical Analysis Plan has been reviewed and approved by:**					
Name of Approver:	Lee Middleton	Role:	Statistics Team Leader	Affiliation:	BCTU<br>University of Birmingham
Signature of Approver:	Lee Middleton	Date:	15/11/2022		

Statistical Analysis Plan (SAP) Amendments

SAP version number	SAP section number	Description of and reason for change	Timing of change with respect to interim analysis/ final analysis/ database lock	Blind Reviewer	
2.0	-	SAP updated into new version template. This includes the addition of the 'data manipulations' section (section 9.4) which details how the statistician will derive values prior to analysis.	Prior to final analysis/database lock.	Name:<br>Signature:<br>Date:	Samir Mehta<br>Samir Mehta (eSigned)<br>14/11/2022
2.0	4.6/9.5	Outcome previously defined as 'Further diagnostic and therapeutic surgery for endometriosis and use of analgesia (as a proxy for recurrence)' now defined as ' Further therapeutic surgery or second-line treatment for endometriosis as a proxy for recurrence or 'treatment failure', defined as having undergone hysterectomy, surgery for endometriosis, laparoscopy or taking GnRH treatment. Further analysis will also include returning to pre-randomisation state in terms of EHP-30 pain score, along with any of the aforementioned interventions'. Details of how this outcome will be analysed now provided.	Prior to final analysis/database lock.	Name:<br>Signature:<br>Date:	Samir Mehta<br>Samir Mehta (eSigned)<br>14/11/2022
2.0	5.1	Clarification that p-values will be produced for the primary outcome and Serious Adverse Events (SAEs) only due to issues around multiplicity.	Prior to final analysis/database lock.	Name:<br>Signature:<br>Date:	Samir Mehta<br>Samir Mehta (eSigned)<br>14/11/2022
2.0	5.3	Clarification of which participants randomised under different combinations of randomisation options in the pilot phase (under v1.0 of the protocol) will be included in the final analysis population.	Prior to final analysis/database lock.	Name:<br>Signature:<br>Date:	Samir Mehta<br>Samir Mehta (eSigned)<br>14/11/2022
2.0	5.3/9.9	Clarification that 'per protocol' analysis will not be performed; all analysis	Prior to final	Name:	Samir Mehta

		will be performed in the ITT population.	analysis/database lock.	Signature:<br>Date:	Samir Mehta (eSigned)<br>14/11/2022
2.0	5.4/7.2	Clarification of how adherence data will be reported.	Prior to final analysis/database lock.	Name:<br>Signature:<br>Date:	Samir Mehta<br>Samir Mehta (eSigned)<br>14/11/2022
2.0	5.5/9.9	Definition of timeliness of returned questionnaires (‘on time’/’late’) revised. Details on how the impact of late forms will be explored is now included in the sensitivity analysis section.	Prior to final analysis/database lock.	Name:<br>Signature:<br>Date:	Samir Mehta<br>Samir Mehta (eSigned)<br>14/11/2022
2.0	9.1	Covariates in adjustment to be treated in same form as minimisation algorithm (previously stated continuous-type variables to be treated as such but this creates a problem downstream in terms of subgroup analyses); attempts will be made to include centre as a random effect for all outcomes, binary included (as opposed to fixed effects). Clarification also of how the variables: *selection of LNG-IUS or DMPA if randomised to LARC* and *selection of LARC was due to patient preference or not* will be included in the model as this data was not collected on all participants.	Prior to final analysis/database lock.	Name:<br>Signature:<br>Date:	Samir Mehta<br>Samir Mehta (eSigned)<br>14/11/2022
2.0	9.1/9.4/9.5/9.9	Clarification that any longitudinal data will be analysed using mixed models incorporating all assessment times (as opposed to separate regression at each time point).	Prior to final analysis/database lock.	Name:<br>Signature:<br>Date:	Samir Mehta<br>Samir Mehta (eSigned)<br>14/11/2022
2.0	9.3/9.9	New approach for handling missing data is outlined which now includes a MNAR (Missing Not At Random) sensitivity analysis approach.	Prior to final analysis/database lock.	Name:<br>Signature:<br>Date:	Samir Mehta<br>Samir Mehta (eSigned)<br>14/11/2022
2.0	9.5	Any longitudinal binary or ordinal data now analysed using a GEE approach incorporating all assessment times (as opposed to separate regression at each time point); odds ratios will be produced as opposed to risk ratios.	Prior to final analysis/database lock.	Name:<br>Signature:	Samir Mehta<br>Samir Mehta (eSigned)

		Clarification of analysis populations for VAS score responses and answer to 'How regular is your cycle?' question.		Date:	14/11/2022
2.0	9.9	Sensitivity analysis on additional endometriosis surgery on EHP-30 pain scores (carried over score) now superseded by time-to-event 'treatment failure' analysis and removed.	Prior to final analysis/database lock.	Name:	Samir Mehta
				Signature:	Samir Mehta (eSigned)
				Date:	14/11/2022
2.0	10	Clarification on how data collected from participants randomised to combinations that did not involve COCP in the pilot phase (LNG-IUS v no treatment; DMPA v no treatment; LNG-IUS v DMPA v no treatment) will be reported.	Prior to final analysis/database lock.	Name:	Samir Mehta
				Signature:	Samir Mehta (eSigned)
				Date:	14/11/2022

Abbreviations & Definitions	
**Abbreviation / Acronym**	**Meaning**
BCTU	Birmingham Clinical Trials Unit
CONSORT	Consolidated Standards of Reporting Trials
DMC	Data Monitoring Committee
ISRCTN	International Standard Randomised Controlled Trial Number
ITT	Intention to Treat
SAE	Serious Adverse Event
SAP	Statistical Analysis Plan
SUSAR	Suspected Unexpected Serious Adverse Reaction
TSC	Trial Steering Committee
DMPA	Depot-medroxyprogesterone acetate injection
LNG-IUS	Levonorgestrel-releasing intra-uterine system (Mirena)
COCP	Combined oral contraceptive pill
LARC	Long-acting reversible contraceptive
EHP-30	Endometriosis Health Profile
EQ-5D-5L	Euroqol Questionnaire
ICE-CAP	Ice-Cap Capability measure for Adults
**Term**	**Definition**
International Standard Randomised Controlled Trial Number	A clinical trial registry
Protocol	Document that details the rationale, objectives, design, methodology and statistical considerations of the study
Randomisation	The process of assigning trial subjects to intervention or control groups using an element of chance to determine the assignments in order to reduce bias.
Statistical Analysis Plan	Pre-specified statistical methodology documented for the trial, either in the protocol or in a separate document.

TABLE OF CONTENTS

1. Introduction 8
2. Background and rationale 8
3. Trial objectives 8
4. Trial methods 9
4.1. Trial design 9
4.2. Trial interventions 9
4.3. Randomisation 10
4.4. Timing of outcome assessments 10
4.5. Primary outcome measure 11
4.6. Secondary outcome measures 11
4.7. Sample size 12
4.8. Framework 12
4.9. Interim analyses and stopping guidance 13
4.10. Internal Pilot Progression Rules 13
4.11. Timing of final analysis 14
4.12. Timing of other analyses 14
4.13. Trial comparisons 14
5. Statistical Principles 14
5.1. Confidence intervals and p-values 14
5.2. Adjustments for multiplicity 14
5.3. Analysis populations 14
5.4. Definition of adherence 15
5.5. Handing protocol deviations 16
5.6. Unblinding 17
6. Trial population 17
6.1. Recruitment 17
6.2. Baseline characteristics 17
7. Intervention(s) 17
7.1. Description of the intervention(s) 17
7.2. Adherence to allocated intervention 18
8. Protocol deviations 18
9. Analysis methods 18
9.1. Covariate adjustment 18
9.2. Distributional assumptions and outlying responses 19
9.3. Handling missing data 19
9.4. Analysis methods – primary outcome 19
9.5. Analysis methods – secondary outcomes 20
9.6. Analysis methods – exploratory outcomes and analyses 21
9.7. Safety data 21
9.8. Planned subgroup analyses 21
9.9. Sensitivity and supportive analyses 22
10. Analysis of sub-randomisations 23
11. Health economic analysis 23
12. Statistical software 23
13. References 23
Appendix A: Deviations from SAP 25
Appendix B: Trial schema 25
Appendix C: Schedule of assessments 26
Appendix D: Data manipulations 26

Appendix E1: CONSORT flow diagram 33
Appendix E2: Baseline characteristics 34
Appendix E3: Description of initial administration of intervention(s) 35
Appendix E4: Adherence to allocated intervention 36
Appendix E5: Compliance to allocated intervention 36
Appendix E6: Protocol deviations 37
Appendix E7: Primary outcome results 37
Appendix E8: Secondary outcomes results 39
Appendix E9: Safety 46
Appendix E10: data summaries from participants randomised to combinations that did not involve COCP 47

1. Introduction

This document is the Statistical Analysis Plan (SAP) for the PRE-EMPT trial, and should be read in conjunction with the current trial protocol. This SAP details the proposed analyses and presentation of the data for the main paper(s) reporting the results for the PRE-EMPT trial.

The results reported in these papers will follow the strategy set out here. Subsequent analyses of a more exploratory nature will not be bound by this strategy, though they are expected to follow the broad principles laid down here. The principles are not intended to curtail exploratory analysis (e.g. to decide cut-points for categorisation of continuous variables), nor to prohibit accepted practices (e.g. transformation of data prior to analysis), but they are intended to establish rules that will be followed, as closely as possible, when analysing and reporting data.

Any deviations from this SAP will be described and justified in the final report or publication of the trial (using a table as shown in Appendix A). The analysis will be carried out by an appropriately qualified statistician, who should ensure integrity of the data during their data cleaning processes.

2. Background and rationale

The background and rationale for the trial are outlined in detail in the protocol. In brief, PRE-EMPT is a randomised, pragmatic, multicentre trial evaluating the clinical and cost-effectiveness of long acting reversible contraceptives (LARCs) in preventing recurrence of endometriosis.

3. Trial objectives

The primary objective is to compare, in women undergoing conservative surgery for pain due to endometriosis, the effectiveness of LARCs compared to COCP in preventing the recurrence of symptoms and improving quality of life.

Secondary objectives are as follows:

- To compare LNG-IUS versus COCP and DMPA versus COCP by carrying out subgroup analysis on the pre-randomisation choice of LARC.
- To compare LARCs versus COCP in terms of the amount of pelvic pain experienced, fatigue, menstrual regularity, repeat surgery and serious side-effects.
- To compare the relative cost effectiveness of alternative hormonal interventions, DMPA, LNG-IUS and COCP for the prevention of recurrent endometriosis. The main comparator will be COCP (the analysis of which will be detailed elsewhere).

4. Trial methods

4.1. Trial design

PRE-EMPT is a multicentre, open-label, randomised, parallel group superiority trial. Patients with endometriosis, due to have conservative surgery, will be recruited from secondary and tertiary care hospitals in the UK (Appendix B).

Participants will be randomized in a 1:1 ratio to LARC or COCP. Selection of LARC (LNG-IUS or DMPA) will be made prior to randomisation to allow balanced subgroup comparisons. For cases where neither the patient nor the clinician has a preference about any of the two LARCs a separate randomization will be performed to allocate a LARC (using a random blocked list of variable length). Surgeons and participants will not remain blind to treatment allocation, due to the nature of the LARC interventions.

4.2. Trial interventions

- **LARC 1: Depot medroxyprogesterone acetate injection (DMPA; Depo-Provera™)**

  Depo-Provera™ is an aqueous suspension of 150mg of depot medroxyprogesterone acetate. It is a long acting reversible preparation that is administered by intramuscular injection every three months. It is approved for use as a contraceptive is used off-label for the management of endometriosis-related pain. The most common side effects are menstrual irregularities (bleeding or amenorrhea), prolonged delay in the resumption of ovulation, weight changes. There is a potential risk of bone demineralisation with long-term use. DMPA is licenced as a contraceptive and acts by preventing ovulation.

- **LARC 2: Levonorgestrel releasing intrauterine system (LNG-IUS; Mirena™/Levosert)**

  The LNG-IUS is a contraceptive system that slowly releases a daily dose of 20 mcg levonorgestrel into the uterine cavity. Bayer Pharma AG market their LNG-IUS under the name of Mirena™ and Actavis UK Ltd under the name of Levosert It is a long acting reversible preparation that requires removal and reinsertion every five years, 3 years for Levosert. LNG-IUS is approved for use as a contraceptive and for heavy menstrual bleeding, prevention of endometrial hyperplasia during oestrogen replacement therapy but is widely used in management of endometriosis-related pain. The most common side effect is unscheduled menstrual bleeding, although this often resolves within 6 months. Documented risks include uterine perforation and infection. The LNG-IUS can also be fitted in primary care. LNG-IUS has an anti-proliferative effect on the endometrium and can prevent ovulation, which may be the mechanism by which it

prevents recurrence of endometriosis.

- **Comparator: Combined oral contraceptive pill (COCP)**

PRE-EMPT will require that women allocated the combined oral contraceptive are prescribed a COCP containing 30mcg ethinylestradiol and 150 mcg levonorgestrel e.g. Microgynon-30 or Rigevidon. This preparation acts systemically to inhibit ovulation and inhibit endometrial growth. Microgynon-30 is the most commonly prescribed COCP, accounting for 41% of all COCP prescriptions, and is one of the cheapest ethinylestradiol preparations. For the management of endometriosis-related pain, it is unclear whether combined oral contraceptives should be taken conventionally, continuously or in tricycle regimen, or whether the dose of ethinylestradiol, or type of progestogen, are important. We will record whether Microgynon-30/Rigevidon is used as a 21-day regimen or continuously. The side effects and risks of combined oral contraceptives have been well investigated(31). Combined oral contraceptives are not recommended in smokers aged ≥35 and women with a BMI ≥35. Like the other PRE-EMPT treatments, the proposed COCP preparations act via anti-proliferation and anti-ovulatory actions, in comparable ways to DMPA and LNG-IUS

4.3. Randomisation

Randomisation will be performed centrally at the Birmingham Clinical Trials Unit using a minimisation algorithm incorporating the following factors:

- Stage of endometriosis (using Classification of the American Society of Reproductive Medicine): I (minimal)/II (mild) versus III (moderate)/ IV (severe)
- Extent of excision of endometriosis: complete versus incomplete, as judged by the surgeon at the time of conservative surgery
- Age in years : <35 versus >=35
- Selection of LNG-IUS or DMPA if randomised to LARC (or random allocation)
- Whether selection of LARC was due to patient preference or not
- Centre, to balance for experience of the gynaecologist

4.4. Timing of outcome assessments

Women who agree to enter the study will complete a baseline participant booklet before randomisation. Booklets will consist of the disease specific and generic quality of life questionnaires, pain scores and resource use questions (see primary and secondary outcomes below). Participants will then be followed up for a period of 36 months (the primary outcome time point). Over this period the booklets will also be collected at 6, 12 and 24 months. Responses at these times will be considered secondary outcomes. The schedule of trial procedures and outcome assessments are given in Appendix C.

4.5. Primary outcome measure

The primary outcome is the recurrence of symptoms as evaluated by the pain domain of the Endometriosis Health Profile-30 (EHP-30)[1] questionnaire at 36 months post-randomisation.

The EHP-30 is a validated, responsive health related quality of life measure for endometriosis. The pain scale (consisting of 11 questions) will be completed by the patient and an overall 0 (best outcome) - 100 pain score (worst score) will then be calculated.

4.6. Secondary outcome measures

The secondary outcomes are as follows:

- The pain domain of the EHP-30 at the other assessment points
- The remaining four core domains of the EHP-30 questionnaire.
    - Control and powerlessness (0=best outcome, 100=worst outcome).
    - Emotional well-being (0=best outcome, 100=worst outcome).
    - Social support (0=best outcome, 100=worst outcome).
    - Self-image (0=best outcome, 100=worst outcome).
- The six modular domains of the EHP-30 questionnaire.
    - Work (0=best outcome, 100=worst outcome).
    - Relationship with family (0=best outcome, 100=worst outcome).
    - Sexual relationship (0=best outcome, 100=worst outcome).
    - Feelings about medical profession (0=best outcome, 100=worst outcome).
    - Feelings about treatment (0=best outcome, 100=worst outcome).
    - Feelings about infertility (0=best outcome, 100=worst outcome).
- Pelvic pain measured by visual analogue scale (VAS) (three scales: pelvic pain during periods [0=best outcome, 10=worst outcome], pelvic pain during intercourse [0=best outcome, 10=worst outcome], pelvic pain at any times [0=best outcome, 10=worst outcome]).
- Responses to the question 'compared to one month ago, would you say your pelvic pain has "Got much better", "Got a little better", "Not changed much", "Got worse".
- Fatigue, as measured by Fatigue Severity Score[2] [7=best outcome, 63=worst outcome], which is the sum of the responses of the nine statements contained in the questionnaire.
- Menstrual regularity. Patients will be asked whether they are still having periods and, if so, to rate how regular their cycle is in one of the four categories; "Regular", "Fairly regular", "Irregular", and "I have bleeding on and off all the time".
- Generic Quality of Life[3] (EQ-5D)
    - EQ-5D index score (patient completed; -0.59=worst outcome, 1.0=best outcome).
    - EQ-5D health thermometer (patient completed; 0=worst outcome, 100=best

outcome).

- Capabilities, as a measure of wellbeing (ICE-CAP: patient completed; 0=worst outcome, 1.0=best outcome)[4]. A score will be calculated from the five ICE-CAP attributes (attachment, stability, achievement, enjoyment, autonomy).
- Further therapeutic surgery or second-line treatment for endometriosis as a proxy for recurrence or 'treatment failure', defined as having undergone hysterectomy, 'surgery for endometriosis', laparoscopy or taking GnRH treatment. Further analysis will also include returning to pre-randomisation state in terms of EHP-30 pain score, along with any of the aforementioned interventions.
- Discontinuation rates of randomised treatment (time to first treatment change – see section 5.4 for further details), with reasons for change.
- Serious adverse events.

See Appendix D (data manipulations) for how the secondary outcomes will be derived.

4.7. Sample size

The sample size is designed to give 90% power to detect an 8 point difference on the EHP-30 pain domain in our main comparison (LARC v COCP). The estimate of standard deviation is 19 (from pooled baseline data in the internal pilot study) with 95% certainty that this is between 16 and 22. To err on the side of caution we have used 22 in our calculation. To detect an 8 point difference in with 90% power (p=0.05), assuming the SD is 22, will require 160 participants per group, 320 in total. To account for any loss to follow-up – which we are assuming to be 20% - we have inflated the sample size to 200 per group, 400 in total.

Given our internal pilot data suggests a roughly even split between those that will select LNG-IUS or DMPA as their methods of LARC delivery, this will mean we will have approximately 160 women in each subgroup comparison. It is estimated there will be enough participants to detect a 10 point difference with 80% power (p=0.05) in each subgroup (however less power to detect interaction – see section 9.8 for further details on how subgroup analyses will be performed).

4.8. Framework

The objective of the trial is to test the superiority of one intervention to another.

The null hypothesis is that there is no difference in the recurrence of symptoms as evaluated by the pain domain of the Endometriosis Health Profile-30 (EHP-30)[1] questionnaire at 36 months post-randomisation between the intervention groups. The alternative hypothesis is that there is a difference between the groups.

4.9. Interim analyses and stopping guidance

A separate Data Monitoring Committee (DMC) reporting template will be drafted and agreed by the DMC including an agreement on which outcomes will be reported at interim analyses. The statistical methods stated in this SAP will be followed for the outcomes included in the DMC report, where possible.

If LARCs are overwhelmingly better or worse than COCP with respect to the primary outcome or other major end-point, then this may become apparent before the target recruitment has been reached. Alternatively, new evidence might emerge from other sources that UPA is definitely more, or less, effective than LNG-IUS. To protect against this, during the main period of recruitment to the study, interim analyses of the primary outcome and adverse events will be supplied, in strict confidence, to the independent DMC, along with updates on results of other related studies, and any other analyses that the DMC may request.

The DMC will advise the chair of the TSC if, in their view, any of the comparisons in the trial have provided both (a) "proof beyond reasonable doubt" that for all, or for some, types of patient one particular treatment is definitely indicated or definitely contraindicated in terms of a net difference in the major endpoints, and (b) evidence that might reasonably be expected to influence the patient management of many clinicians who are already aware of the other main trial results. The TSC can then decide whether to close or modify any part of the trial. Unless this happens, however, the TMG, TSC, the investigators and all of the central administrative staff (except the statisticians who supply the confidential analyses) will remain unaware of the interim results.

Appropriate criteria of proof beyond reasonable doubt cannot be specified precisely, but a difference of at least $p<0.001$ (similar to a Haybittle-Peto[5] stopping boundary) in an interim analysis of a major endpoint may be required to justify halting, or modifying, the study prematurely. If this criterion were to be adopted, it would have the practical advantage that the exact number of interim analyses would be of little importance, so no fixed schedule is proposed.

A separate DMC reporting template will be drafted and agreed by the DMC including an agreement on which outcomes will be reported at interim stage. The statistical methods stated in this SAP will be followed for the agreed outcomes.

4.10. Internal Pilot Progression Rules

Before the start of the PRE-EMPT Trial, we anticipated there will be significant preferences for or against certain treatment groups, from both clinician and women, and the internal pilot study was designed with a flexible randomisation option to accommodate these. The results of this are presented elsewhere[6] and this version of the SAP has taken the re-engineered study

into account.

4.11. Timing of final analysis

The final analysis for the trial will occur after the final participant recruited reaches the 3 year follow-up time point and the corresponding outcome data has been entered onto the trial database and validated as being ready for analysis. This is provided that the trial has not been stopped early for any reason (e.g. DMC advice or funding body request).

4.12. Timing of other analyses

Not applicable; participants will not be followed up post three years; no further analysis is planned.

4.13. Trial comparisons

The main comparison is long-acting reversible contraception (LARC) versus combined oral contraceptive pill (COCP); all references in this document to 'group' refer to one of these two groups.

Sub-comparisons include LARC v COCP in those that selected to be treated with LNG-IUS (if randomised to LARC) and LARC v COCP in those that selected to be treated with DMPA (if randomised to LARC). These will be considered secondary comparisons.

5. Statistical Principles

5.1. Confidence intervals and p-values

A p-value will be reported from a two-sided test at the 5% significance level for the primary outcome and Serious Adverse Events only (safety outcomes can be subject to statistical testing without adjustment for multiple testing as adjustment for multiplicity is counterproductive for considerations of safety[7]). All estimates of differences between groups for all outcomes will be presented with two-sided 95% confidence intervals, unless otherwise stated (see further point in section 5.2).

5.2. Adjustments for multiplicity

No correction for multiple testing will be made; cautious interpretation of secondary outcome confidence intervals will be necessary due to the possibility of multiplicity.

5.3. Analysis populations

All primary analyses (primary and secondary outcomes including safety outcomes) will be based on the intention-to-treat (ITT) principle. Participants will be analysed in the intervention

group to which they were randomised, and all participants shall be included regardless of whether they received the allocated intervention, provided they were randomised to a 'LARC versus COCP' comparison (see next paragraph). This is to avoid any potential bias in the analysis. The principle behind LARC administration is that they eliminate the need for daily administration, potentially improving adherence in comparison to other treatment options (like COCP, the comparator group). Hence, given the main advantage of LARCs is increased adherence, exploration of other analysis populations – such as 'per protocol' (adherent population) – is considered to have limited value and will not be conducted.

All participants recruited under versions 2.0-7.0 of the protocol (from 23/10/15) will be included in the final analysis population, as these participants were randomised directly to 'LARC' or 'COCP' (with pre-randomisation choice of LNG-IUS or DMPA if allocated LARC). For those randomised under v1.0 of the protocol (pilot phase 25/7/13), participants will be also be included in the final analysis population provided they were randomised to combinations of treatments that only included LARCs and COCP (LNG-IUS v COCP; DMPA v COCP, LNG-IUS v DMPA v COCP) or combinations of treatments that included LARCs, COCP and no treatment but were not ultimately randomised to no treatment (LNG-IUS v COCP v no treatment; DMPA v COCP v no treatment; all treatments). Those participants randomised to combinations that did not involve COCP (LNG-IUS v no treatment; DMPA v no treatment; LNG-IUS v DMPA v no treatment) will not be included in the final analysis population; details of how responses will be summarised for these participants is detailed in section 10. Further details of the pilot phase are provided elsewhere.[6]

5.4. Definition of adherence

First treatment changes and reasons for changing will be reported to aid the interpretation of the results, but will not contribute to any further analyses.

Participants will be considered to be on their assigned trial treatment (only) whilst they fulfil the following criteria: in those allocated LNG-IUS, these will be women who have had the device fitted following allocation and have not reported having had the device removed at the corresponding assessment time-point. This will be self-reported on the follow-up questionnaire [LNG-IUS='Yes']. In those allocated DMPA or COCP, this comprises those who have confirmed that they have started their initial treatment and are confirmed to be still be on treatment at the time of assessment [Depo-provera='Yes' or Contraceptive pill [any brand]='Yes'])

Otherwise, first treatment changes during the follow-up period will reported in the following categories by group:

- On assigned trial treatment (as per previous definition) plus any another trial treatment (LNG-IUS/COCP/DMPA)
- On assigned trial treatment (as per previous definition) plus taking any other non-trial

treatment (such as, but not limited to: POP/mini-pill/Zoladex/Kliofem/Decapeptyl/prostap/implanon/nexplanon)

- Not on assigned trial treatment (as per previous definition), taking any other trial treatment (LNG-IUS/COCP/DMPA)
- Not on assigned trial treatment (as per previous definition), taking any other non-trial treatment (such as, but not limited to: POP/mini-pill/Zoladex/Kliofem/Decapeptyl/prostap/implanon/nexplanon)
- Not taking any treatment

In the situation where a participant has changed to both another trial treatment and non-trial treatment these will be listed as a change to another trial treatment only to minimise complexity.

Use of GnRH analogues will be considered a proxy for recurrence or 'treatment failure', along with hysterectomy, 'surgery for endometriosis' and laparoscopy. This will be reported within the separate outcome described in section 9.5. If any of these events occur before the first treatment change then this will be indicated in the results and reports of further treatment changes will not be considered.

Data will be taken from the self-reported participant questionnaire booklet and, where appropriate, supplemented with information taken from hospital records. Reports of different treatment use on the questionnaire will be prioritised oved the 'treatment change' part of the questionnaire if information is inconsistent.

Changes on regime of COCP (continuous or cyclical) will not be classified as a treatment change. 'Combined pill' will be classified within the COCP family; POP and progesterone only 'mini-pill' will not.

5.5. Handing protocol deviations

A protocol deviation is defined as a failure to adhere to the protocol such as errors in applying the inclusion/exclusion criteria, the incorrect intervention being given, incorrect data being collected or measured, follow-up visits outside the visit window or missed follow-up visits. We will apply a strict definition of the ITT principle and will include all participants as per the ITT population described in section 5.3 in the analysis, in some form, regardless of deviation from the protocol.[8] This does not include those participants who have specifically withdrawn consent for the use of their data in the first instance; however these outcomes will be explored as per other missing responses.

Six month follow-up questionnaires will be considered to have been completed on time if they have been completed prior to the one year assessment time point (1 year post-randomisation). One year follow-up questionnaires will be considered on time provided they have been

completed prior to the two year assessment time point (2 years post-randomisation). Two year follow-up questionnaires will be considered on time provided they have been completed before three year assessment time point (three years post-randomisation). Three year follow-up questionnaires will be considered on time provided they have been completed before 4 years post-randomisation. If questionnaires are completed too late to be considered valid for that particular assessment time and subsequent questionnaires have not been returned, they will be considered valid for the subsequent time-point (e.g. a six month form completed at thirteen months but with twelve month form missing will be considered valid for the twelve month time-point). In the first instance, all data will be included in the primary analysis regardless of date of completion, but sensitivity analysis will be conducted on the primary outcomes excluding any late responses (see section 9.9).

5.6. Unblinding

Not applicable, PRE-EMPT is an open-label study.

6. Trial population

6.1. Recruitment

A flow diagram (as recommended by CONSORT[9]) will be produced to describe the participant flow through each stage of the trial. This will include information on the number (with reasons) of losses to follow-up (drop-outs and withdrawals) over the course of the trial. A template for reporting this is given in Appendix E1.

6.2. Baseline characteristics

The trial population will be tabulated as per Appendix E2. Categorical data will be summarised by number of participants, counts and percentages. Continuous data will be summarised by the number of participants, mean and standard deviation if deemed to be normally distributed or number of participants, median and interquartile range if data are skewed, and ranges if appropriate. Tests of statistical significance will not be undertaken, nor confidence intervals presented.[10]

7. Intervention(s)

7.1. Description of the intervention(s)

A template for reporting information on the initial administration of the intervention(s) is given in Appendix E3.

7.2. Adherence to allocated intervention

A template for reporting adherence is given in Appendices E4 and E5. Data will be presented as time-to-event data (first treatment change using categories detailed in Appendix 5) using a Kaplan Meier plot. Participants will be censored at the last point of known follow-up if they have not had an event. If date of treatment change is not available then this will be taken to be the half-way point between reported dates of questionnaire completion (e.g. half-way between the 1 and 2 year form completion dates).

8. Protocol deviations

Frequencies and percentages by group will be tabulated for the protocol deviations as per Appendix E6.

9. Analysis methods

Intervention groups will be compared using appropriate regression models dependent on the data-type and will adjust for all covariates as specified in section 9.1, where possible.

9.1. Covariate adjustment

In the first instance, comparative estimates of differences between groups will be adjusted for the parameters listed in section 4.3 in the same form as entered into the minimisation algorithm, i.e. categorical variables such as age will remain as such in the model, rather than converting them to the continuous form (as this creates a problem downstream in terms of presentation of the subgroup analyses).

The variable *selection of LNG-IUS or DMPA if randomised to LARC* will include three categories: LNG-IUS, DMPA, and, allocate randomly (for those entering in the pilot stage who were happy to be randomised to either LNG-IUS or DMPA this will be assumed to be allocate randomly).

The variable *selection of LARC was due to patient preference or not* will include three categories: patient preference, clinician advice, and, 'other' (the latter category for those where this question did not exist on an older version of the randomisation form used in the pilot phase or where they selected allocate randomly in the main phase of the study).

Where repeated assessments (longitudinal data) have been made, data from all assessment times will be included in the estimation models. Parameters for participant, treatment group, time and baseline response (as a continuous variable) will be included. Time will be assumed to be a categorical (fixed) variable. To allow for a varying treatment effect over time, a time by treatment interaction parameter will also be included in the model.

Centre will be included as a random intercept in the model, and all other factors as fixed effects (with the exception of time to event outcomes where centre will be regarded as a fixed effect).

If covariate adjustment is not possible (e.g. the model does not converge), randomising centre will be removed first. If this reduced model still fails to converge, unadjusted estimates will be produced and it will be made clear in the final report why this occurred (e.g. not possible due to low event rate/lack of model convergence).

9.2. Distributional assumptions and outlying responses

Distributional assumptions (e.g. normality of data and/or regression residuals for continuous outcomes) will be assessed visually prior to reporting the results of the analysis. Although in the first instance the proposed primary method of estimation in this analysis plan will be followed, if distributional assumptions are considered to be violated, the impact of this will be examined through sensitivity analysis; this may consist of transformation of responses prior to analysis (e.g. log transformation) in the first instance. If extreme values are apparent and considered to be affecting the integrity of the analysis, a sensitivity analysis consisting of removing the outlying response(s) and repeating the analysis will be performed. Output from these analyses, if performed, will be described and presented alongside the original analysis (or included, e.g. in appendices) with the excluded values clearly labelled. See section 9.9 for further details regarding sensitivity analyses.

9.3. Handling missing data

In the first instance, analysis will be completed on observed data only with every effort made to follow-up participants to minimise any potential for bias. For the primary outcome, a maximum likelihood approach will be utilised which has been shown to be analogous to multiple imputation (MI) in terms of how missing data is handled[12]; missing data will be assumed to be missing at random (MAR) given the parameters in the model. To test the assumption of missing responses not being MAR, and to make sure we are complying with the intention-to-treat principle,[13] sensitivity analyses assuming missing data to be 'Missing Not At Random (MNAR)' will be employed on the primary outcome measure. See section 9.9 for further details regarding sensitivity analyses.

9.4. Analysis methods – primary outcome

See Appendix D for information on how variables will be derived for the analysis. A template for reporting the primary outcome is given in Appendix E7.

A mixed linear regression model for repeated measures[14] will be used to calculate an adjusted difference between groups means (at the primary outcome time point of 36 months) along

with 95% confidence intervals. A compound symmetry (CS) covariance structure will be assumed. See section 9.1 for parameters to be included in the model. An F-test will be used to test statistical significance (p-value produced) of the estimated treatment group parameter generated from the maximum likelihood estimate.

9.5. Analysis methods – secondary outcomes

See Appendix D for information on how variables will be derived for the analysis. A template for reporting the secondary outcomes is given in Appendix E8.

Analysis will be performed as per the primary outcome for the following patient reported summary scores: EHP-30 pain score at 6, 12 and 24 months (calculated from within the same model as tthe primary outcome but with estimates made at these time points); any other EHP-30 domain; EQ-5D index score and thermometer; ICE-CAP score, FSS score, and VAS responses. For VAS scores, if the participant answers 'not applicable' to *no regular periods* or *not sexually active,* they will not be included in these analyses.

Responses to 'Are you still having periods (yes/no)?' will be analysed using a generalised estimating equation (GEE) model[15] with logit link that will take into account all assessment times (correlated longitudinal data) and adjusting for the parameters listed in section 9.1. An 'independent' covariance structure will be assumed. Odds ratios and 95% confidence intervals for the treatment group parameter will be produced.

For cycle regularity the responses will be dichotomised as i) regular (patients who reported "Regular, I know when to expect my period" and "Fairly regular, my periods starts within a few days of when I expect") and ii) irregular ("Irregular, I cannot predict when my period will start" and "I have bleeding on and off all the time"). This data will be analysed in the same fashion as the 'Are you still having periods (yes/no)? question and will only include participants who have stated they are still having periods.

Improvement of pelvic pain compare to a month ago ("Got much better", "Got a little better", "Not changed much", "Got worse") will be analysed using a generalised estimating equation (GEE) model with cumulative logit link (for ordered categorical data) that will take into account all assessment times (i.e. correlated longitudinal data) and adjusting for parameters listed in section 9.1. A general 'independent' covariance structure will be assumed. Cumulative odds ratios and 95% confidence intervals for the treatment group parameter will be produced.

Further therapeutic surgery or second-line treatment for endometriosis (defined as having undergone hysterectomy, 'surgery for endometriosis', laparoscopy or taking GnRH treatment) will be analysed as time-to-event data using a Cox regression model. Adjusted hazard ratios (and 95% confidence intervals) will be generated and a Kaplan Meier plot will be produced to assess the data visually. In this model centre will be regarded as a fixed effect. Participants will

be censored at the last point of known follow-up if they have not had an event. If date of event is not available then this will be taken to be the half-way point between reported dates of questionnaire completion (e.g. half-way between the 1 and 2 year form completion dates). Further analysis along the same lines will be completed, but this time the date of returning to pre-randomisation state in terms of EHP-30 pain score (i.e. at the first assessment time where the EHP-30 pain score minus pre-randomisation score is <=0) will also be classified as failure (again the half-way point between dates of form completion will be utilised here as exact date of returning to this state will be unknown). If the participant maintains a score>0 throughout then they will be treated as censored at the last follow-up time observed (provided they have not had one of the two types of operation as detailed above). Missing interim assessments will be assumed to be >0 if a later assessment is available. Those with no follow-up will be censored at time 0.

Other forms of surgery, e.g. removal of polyps, removal of fibroids, endometrial ablation will be summarised in tables but not formally analysed.

9.6. Analysis methods – exploratory outcomes and analyses

Any data that does not form a pre-specified outcome will be presented using simple summary statistics by intervention group (i.e. numbers and percentages for binary data and means (or medians) and standard deviations (or inter-quartile ranges) for continuous normal (or non-normal) data.

Reasons for changing treatment, as well as additional measures listed in section 4.6 will be summarized by group but not be formally analysed.

9.7. Safety data

The number and percentage of participants experiencing any adverse events, serious adverse events (SAEs) and suspected unexpected serious adverse reactions (SUSARs) will be presented by intervention group. Statistical significance will be determined by chi-squared test. A template for reporting this safety data is given in Appendix E9.

9.8. Planned subgroup analyses

Subgroup analyses will investigate whether there was any effect of LARC selection prior to randomisation. The following subgroup analyses will be carried out and limited to the primary outcome (at the 3-year time-point) only:

- Pre-randomisation selection of LNG-IUS or DMPA including all methods of allocation (this includes random allocation; note this will exclude any participants who were happy to be randomised to both LNG-IUS and DMPA in the pilot)

- Pre-randomisation selection of LNG-IUS or DMPA excluding random allocation
- Pre-randomisation selection of LNG-IUS or DMPA including only those participants where the LARC was specifically chosen by the patient

In addition, the following subgroup analyses will be carried out:

- Stage of endometriosis (using Classification of the American Society of Reproductive Medicine): I (minimal)/II (mild) versus III (moderate)/ IV (severe)
- Extent of excision of endometriosis: complete versus incomplete, as judged by the surgeon at the time of conservative surgery
- Age in years : <35 versus >=35

The effects of these subgroups will be examined by adding the subgroup by treatment group interaction parameters to the linear model described above. To allow for the possibility of differential changes over time within the different subgroups, time by subgroup and the three way interaction between treatment, time and subgroup will also be included in the model. Statistical significance of these interaction parameters will be determined by F-tests/Wald tests. Differences between treatment groups within subgroups and 95% confidence intervals will be generated; these will be generated by producing differences between groups through the model that includes the relevant interaction parameter.

9.9. Sensitivity and supportive analyses

Sensitivity and supportive analyses will be limited to the EHP-30 pain responses (at the 3-year time-point) and will consist of:

- Sensitivity analysis to investigate the assumption that missing data is MAR using a delta-based approach[16], which assumes missing data is MNAR. Missing data will be imputed using MI with chained equations in Stata 17 (or above). Stata's "MI" command will be used to carry out this analysis. 50 imputations will be generated; all variables that were included in the analysis of this outcome (see section 10.7) will be included to ensure compatibility of approach. The average increase of observed data at each time will be calculated; a value 'delta' which is equivalent to a proportion of this average increase will then be subtracted from the imputed value in all of the imputation sets. The delta values will be taken in turn as the following in separate investigations:
    - 20% of average increase in both groups at each time point
    - 20% of average increase in the LARC group at each time point; 10% in the COCP treatment group
    - 10% of average increase in the LARC group at each time point; 20% in the COCP treatment group

Finally, for each of these investigations, analysis will then be performed as per the original approach (see section 10.7) on the imputed and manipulated sets (and hence data will need to be imputed at all time points given the repeated measures model), with results combined using Stata's "mi estimate" command which uses Rubin's rule[17] to obtain a single set of results.

- Analysis removing any late responses as defined in section 5.5.

10. Analysis of sub-randomisations

Summary statistics will be provided for the primary outcome (only) for those participants randomised to combinations that did not involve COCP: LNG-IUS v no treatment; DMPA v no treatment; LNG-IUS v DMPA v no treatment. This data will be summarised in 'LARC' and 'no treatment' groups. No formal analysis will be performed. Details of how responses will be summarised for these participants is detailed in Appendix E10.

For those patients who were happy to have their LARC randomly allocated (LNG-IUS versus DMPA) prior to the LARC v COCP allocation we potentially have an unbiased random comparison. The output from any such comparison will be planned and reported separately.

11. Health economic analysis

As indicated in the protocol there will also be an economic analysis. The details of this analysis are documented separately.

12. Statistical software

Statistical analysis will be undertaken in the following statistical software packages: SAS software, version 9.4 (or higher) or STATA version 17 (or higher).

13. References

1. Measuring quality of life in women with endometriosis: tests for data quality, score reliability, response rate and scaling assumptions of the Endometriosis Health Profile Questionnaire. Hum Reprod. 2006; 21(10):2686-93.
2. Krupp LB, LaRocca NG, Muir-Nash J, Steinberg AD. The fatigue severity scale. Application to patients with multiple sclerosis and systemic lupus erythematosus. Archives of Neurology. 1989; 46 (10): 1121-3.
3. Herdman M, Gudex C, Lloyd A, Janssen M, Kind P, Parkin D, et al. Development and preliminary testing of new five-level version of EQ-5D (EQ-5D-5L). Qual Life Res. 2011; 20(10): 1727-36.
4. Al-Janabi H, Flynn TN, Coast J. Development of a self-report measure of capability wellbeing for adults: the ICECAP-A. Quality of Life Research. 2012; 21(1):167-76.
5. Peto R, Pike MC, Armitage P, Breslow NE, Cox DR, Howard SV et al. Design and analysis

of randomised clinical trials requiring prolonged observation of each patient. I. Introduction and design. Br J Cancer. 1976;34:585-612.13. White IR, Horton NJ, Carpenter J, Pocock SJ. Strategy for intention to treat analysis in randomised trials with missing outcome data. BMJ. 2011; 342:d40.

6. Middleton, L.J., Daniels, J.P., Weckesser, A. et al. Preventing recurrence of endometriosis by means of long-acting progestogen therapy (PRE-EMPT): report of an internal pilot, multi-arm, randomised controlled trial incorporating flexible entry design and adaption of design based on feasibility of recruitment. Trials 18, 121 (2017).
7. Huque MF, Dmitrienko A and D'Agostino R. Multiplicity issues in clinical trials with multiple objectives. Statistics in Biopharmaceutical Research. 2013 5:4,321-337.
8. Gupta SK. Intention-to-treat concept: A review. Perspect Clin Res. 2011;2(3):109-112.
9. Schulz KF, Altman DG, Moher D, for the CONSORT Group. CONSORT 2010 Statement: updated guidelines for reporting parallel group randomised trials. BMJ. 2010;340:c332.
10. Altman DG, Dore CJ. Randomisation and baseline comparisons in clinical trials. Lancet. 1990;335:149–53.
11. Zou G. A modified Poisson regression approach to prospective studies with binary data. Am J Epidemiol. 2004;159(7):702-6.
12. Jakobsen, J.C., Gluud, C., Wetterslev, J. et al. When and how should multiple imputation be used for handling missing data in randomised clinical trials – a practical guide with flowcharts. BMC Med Res Methodol 17, 162 (2017)
13. White IR, Horton NJ, Carpenter J, Pocock SJ. Strategy for intention to treat analysis in randomised trials with missing outcome data. BMJ. 2011;342:d40.
14. Brown H, Prescott R. Applied Mixed Models in Medicine, 2nd Edition. Wiley, 2006.
15. Liang KY, Zeger SL. Longitudinal Data Analysis using Generalized Linear Models. Biometrika. 73:13-22.
16. Cro et al. Sensitivity analysis for clinical trials with missing continuous outcome data using controlled multiple imputation: A practical guide. Statistics in Medicine. 2020; 1–28.
17. Rubin DB. Multiple imputation for nonresponse in surveys. Wiley, 1987.

Appendix A: Deviations from SAP

This report below follows the statistical analysis plan version *<x.0>* dated *<insert effective date of latest SAP>* apart from following:

Section of report not following SAP	Reason
<insert section >	<insert, e.g. exploratory analyses request by TMG>

Appendix B: Trial schema

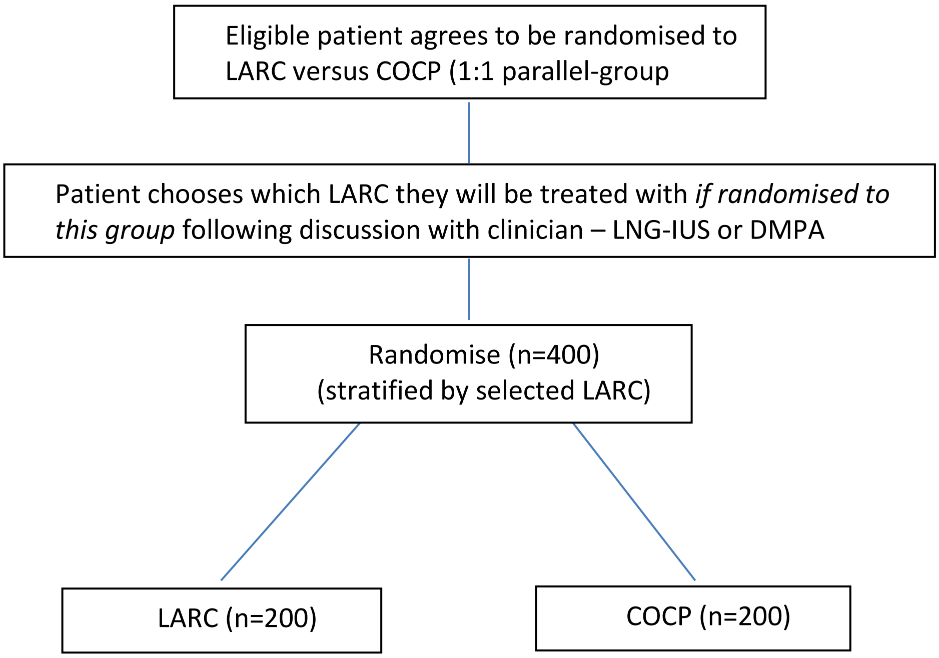

Appendix C: Schedule of assessments

Timepoint<br><br>Outcome measure	Prior to randomisation	Prior to discharge following surgery	6 months	1 year	2 year	3year
**EHP-30**	X		X	X	X	**X**
**FSS**	X		X	X	X	**X**
**EQ-5D and ICECAP**	X		X	X	X	**X**
**Pregnancy**			X	**As reported by participant**		
**Menstrual regularity**	X		X	X	X	**X**
**Serious adverse events**				**As reported by clinician/ participant**		
**Surgical procedure**		X				
**Resource usage (participant)**	X		X	X	X	**X**
**Repeat surgery**				**As reported by participant/clinician**		

Appendix D: Data manipulations

The Trial Statistician will derive all responses from the raw data recorded in the database.

Primary Outcome**:**

- **Recurrence of symptoms as evaluated by the pain (core) domain of the EHP-30[1] questionnaire at 36 Months post-randomisation**

CRF: Follow-up Questionnaire

Question:

- Pain domain: Questions 1-11

To obtain the summary score from the pain domain, the following equation will be used:

Pain domain score = $(Q1 + Q2 + Q3 + Q4 + Q5 + Q6 + Q7 + Q8 + Q10 + Q11 - 11)*100/44$;

Secondary Outcomes**:**

- **Core domain of the EHP-30 questionnaire: Control and powerlessness**

CRF: Follow-up Questionnaire

Question:

- Control domain: Questions 12-17

To obtain the summary score from the control domain, the following equation will be used:

Control domain score = (Q12 + Q13 + Q14 + Q15 + Q16 + Q17 - 6)*100/24;

- **Core domain of the EHP-30 questionnaire: Emotional well-being**

CRF: Follow-up Questionnaire
Question:

- Control domain: Questions 18-23

To obtain the summary score from the Emotional well-being domain, the following equation will be used:
Emotional well-being domain score = (Q18 + Q19 + Q20 + Q21 + Q22 + Q23 - 6)*100/24;

- **Core domain of the EHP-30 questionnaire: Social support**

CRF: Follow-up Questionnaire
Question:

- Control domain: Questions 24-27

To obtain the summary score from the Social support domain, the following equation will be used:

Social support domain score = (Q24 + Q25 + Q26 + Q27 - 4)*100/16;

- **Core domain of the EHP-30 questionnaire: Self-image**

CRF: Follow-up Questionnaire
Question:

- Control domain: Questions 28-30

To obtain the summary score from the Self-image domain, the following equation will be used:

Self-image domain score = (Q28 + Q29 + Q30 - 3)*100/12;

- **Core domain of the EHP-30 questionnaire: Self-image**

CRF: Follow-up Questionnaire
Question:

- Control domain: Questions 28-30

To obtain the summary score from the Self-image domain, the following equation will be used:

Self-image domain score = (Q28 + Q29 + Q30 - 3)*100/12;

All modular domains are prefaced with the question 'If this section is not relevant to you, please tick here', with an indication to move onto the next section. In the cases where the participant has ticked this box then they will be considered missing and not included in the analysis.

- **Modular domain of the EHP-30 questionnaire: Work**

CRF: Follow-up Questionnaire
Question:
- Work domain: Part 2: Modular Questionnaire: Section A: Questions 1-5

To obtain the summary score from the work domain, the following equation will be used:

Work domain score = (Q1 + Q2 + Q3 + Q4 + Q5 - 5)*100/20;

- **Modular domain of the EHP-30 questionnaire: Relationship with child/children**

CRF: Follow-up Questionnaire
Question:
- Relationship with child/children domain: Part 2: Modular Questionnaire: Section B: Questions 1-2

To obtain the summary score from the Relationship with child/children domain, the following equation will be used:

Relationship with child/children domain score = (Q1 + Q2 - 2)*100/8;

- **Modular domain of the EHP-30 questionnaire: Sexual Relationship**

CRF: Follow-up Questionnaire
Question:
- Sexual Relationship domain: Part 2: Modular Questionnaire: Section C: Questions 1-5

To obtain the summary score from the Sexual Relationship domain, the following equation will be used:

Sexual Relationship domain score = (Q1 + Q2 + Q3 + Q4 + Q5 - 5)*100/20;

This domain has options to not answer any of the five component questions ('If not relevant, please tick here'). If this is the case the Sexual Relationship domain score should be calculated from the remaining question responses, e.g. if Q5 was missing then the score would be generated from (Q1 + Q2 + Q3 + Q4 - 4)*100/16. If all five questions are answered "not relevant", the Sexual Relationship domain score will be considered missing and not included in the analysis.

- **Modular domain of the EHP-30 questionnaire: Feelings about medical profession**

CRF: Follow-up Questionnaire
Question:
- Feelings about medical profession domain: Part 2: Modular Questionnaire: Section D: Questions 1-4

To obtain the summary score from the Feelings about medical profession domain, the following equation will be used:

Feelings about medical profession domain score = (Q1 + Q2 + Q3 + Q4 - 4)*100/16;

- **Modular domain of the EHP-30 questionnaire: Feelings about treatment**

CRF: Follow-up Questionnaire
Question:
- Feelings about treatment domain: Part 2: Modular Questionnaire: Section E: Questions 1-3

To obtain the summary score from the Feelings about treatment domain, the following equation will be used:

Feelings about treatment domain score = (Q1 + Q2 + Q3 - 3)*100/12;

- **Modular domain of the EHP-30 questionnaire: Feelings about infertility**

CRF: Follow-up Questionnaire
Question:
- Feelings about infertility domain: Part 2: Modular Questionnaire: Section F: Questions 1-4

To obtain the summary score from the Feelings about infertility domain, the following equation will be used:

Feelings about infertility domain score = (Q1 + Q2 + Q3 + Q4 - 4)*100/16;

- **Pain score using Visual Analogue Scale (VAS)**

CRF: Follow-up Questionnaire
Question:

- (1) Pelvic pain during your periods: Score 0 (No pain at all) – 10 (Worst imaginable pain)
- (2) Pelvic pain during intercourse: Score 0 (No pain at all) – 10 (Worst imaginable pain)
- (3) Pelvic pain at any other times (other than during period or during intercourse) : Score 0 (No pain at all) – 10 (Worst imaginable pain)

- **Fatigue Severity Scale (FSS)**

CRF: Follow-up Questionnaire
Question:
- FSS Questionnaire

The items are scored on a 7 point scale with 1=strongly disagree and 7=strongly agree. The minimum score=9 and maximum score possible=63. Higher the score=greater fatigue severity

FSS Score = (Q1 + Q2 + Q3 + Q4 + Q5 + Q6 + Q7 + Q8 + Q9)/9;
(Mean of all the scores with minimum score being 1 and maximum score being 7. Mean (SD) FSS scores for healthy individuals; 2.3 (0.7). Cut-off score of 4 or more considered indicative of problematic fatigue.)

- **Generic Quality of Life[3] (EQ-5D-5L)**

CRF: Follow-up Questionnaire
Question:
- General Quality of Life

The EQ5D (5 level) will be scored using the Crosswalk index value calculator (found in the following folder: K:\BCTU\BCTU\Statistics\SOPs\SAS Code Depository\Euroqol EQ5D\EQ5D-5L\Mapping EQ-5D-5L to EQ-5D-3L)

The EuroQol Group coordinated a study that administered both the 3-level and 5-level versions of the EQ-5D, in order to develop a "crosswalk" between the EQ-5D-3L value sets and the new EQ-5D-5L descriptive system, resulting in crosswalk value sets for the EQ-5D-5L

A scientific publication by Van Hout et al. (2012) describing the mapping methodology behind the study in detail is published in Value In Health Journal (https://pubmed.ncbi.nlm.nih.gov/22867780/ - van Hout B, Janssen MF, Feng YS, Kohlmann T, Busschbach J, Golicki D, Lloyd A, Scalone L, Kind P, Pickard AS. Interim scoring for the EQ-5D-5L: mapping the EQ-5D-5L to EQ-5D-3L value sets. Value Health. 2012 Jul-Aug;15(5):708-15. doi: 10.1016/j.jval.2012.02.008. Epub 2012 May 24. PMID: 22867780).

- **ICECAP-A**[4]

CRF: Follow-up Questionnaire
Question:
- Overall Quality of Life

The ICECAP-A response scales are coded as follows:

- Question 1 – Feeling settled and secure:
    - I am able to feel settled and secure in all areas of my life = 0.222
    - I am able to feel settled and secure in many areas of my life = 0.191
    - I am able to feel settled and secure in a few areas of my life =0.101
    - I am unable to feel settled and secure in any areas of my life =-0.001
- Question 2 – Love, friendship and support:
    - I can have a lot of love, friendship and support = 0.228
    - I can have quite a lot of love, friendship and support = 0.189
    - I can have a little love, friendship and support = 0.096
    - I cannot have any love, friendship and support = -0.024
- Question 3 – Being independent:
    - I am able to be completely independent = 0.188
    - I am able to be independent in many things = 0.156
    - I am able to be independent in a few things = 0.084
    - I am unable to be at all independent = 0.006
- Question 4 – Achievement and progress:
    - I can achieve and progress in all aspects of my life = 0.181
    - I can achieve and progress in many aspects of my life = 0.159
    - I can achieve and progress in a few aspects of my life = 0.091
    - I cannot achieve and progress in any aspects of my life = 0.021
- Question 5 – Enjoyment and pleasure:
    - I can have a lot of enjoyment and pleasure = 0.181
    - I can have quite a lot of enjoyment and pleasure = 0.154

- I can have a little enjoyment and pleasure = 0.069
- I cannot have any enjoyment and pleasure = -0.003

The ICECAP-A score can be derived from summing all responses, as follows:

- ICECAP-A score = SUM(1-5)

The ICECAP-A score ranges from 0 to 1, where high scores are good. No missing data items are permitted in order to compute a score.

Appendix E1: CONSORT flow diagram

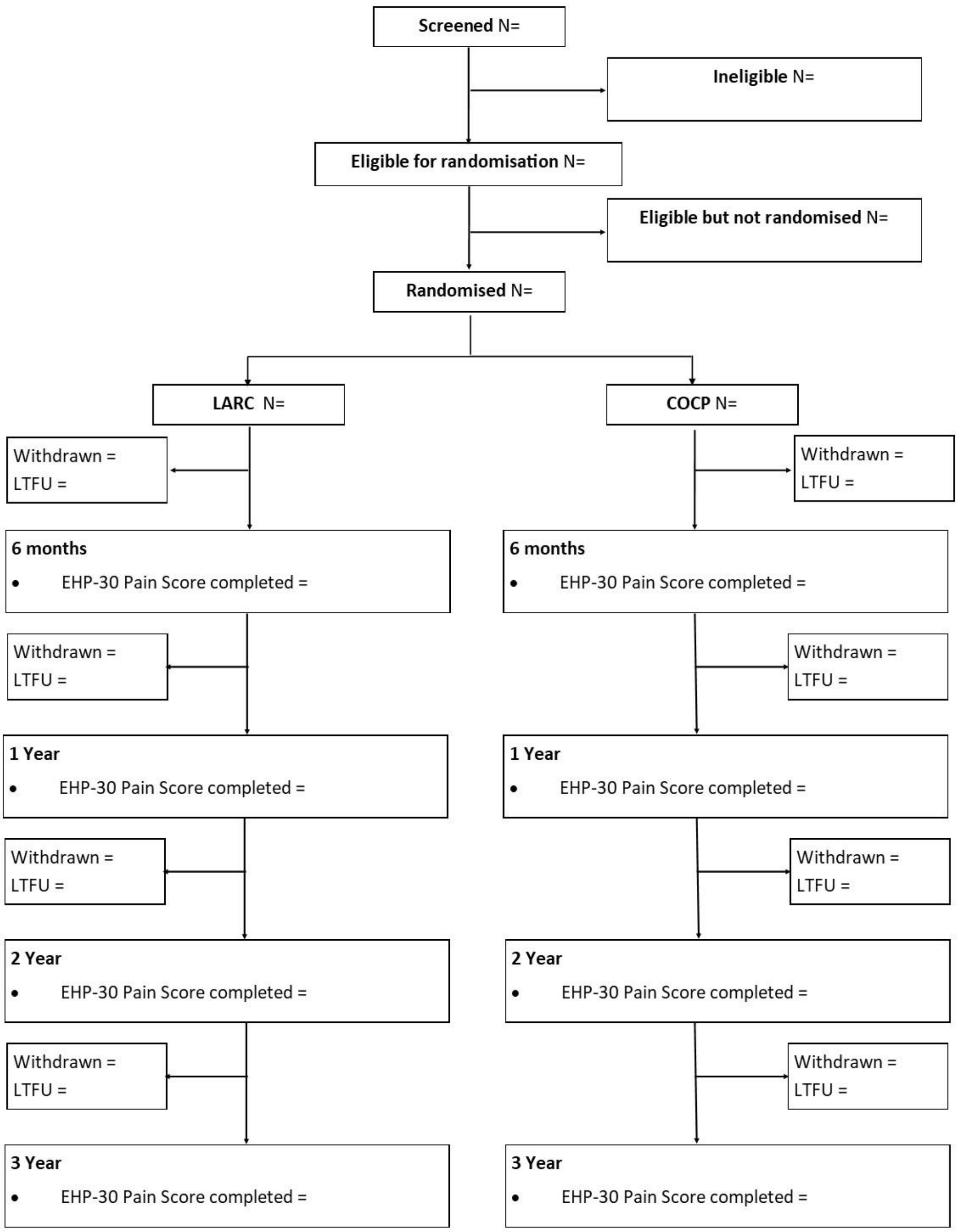

Appendix E2: Baseline characteristics

		LARC (N=)	COCP (N=)	Overall (N=)
**Age[1]**	<35 years	N (%)	N (%)	N (%)
	Mean (SD)			
**BMI**	Mean (SD)			
**Blood Pressure (mm/ Hg)**	Mean (SD)			
**Ever smoked**	Yes	N (%)	N (%)	N (%)
	No	N (%)	N (%)	N (%)
	Missing			
**Extent of excision as judged by surgeon[1]**	Complete			
	Incomplete			
**Centre[1]**	1…	N (%)	N (%)	N (%)
	2…	N (%)	N (%)	N (%)
	3…	N (%)	N (%)	N (%)
	4…	N (%)	N (%)	N (%)
**Stage of Endometriosis[1]**	I/II	N (%)	N (%)	N (%)
	III/IV	N (%)	N (%)	N (%)
**Self-declared Ethnicity**	White British	N (%)	N (%)	N (%)
	Mixed	N (%)	N (%)	N (%)
	Asian	N (%)	N (%)	N (%)
	Black	N (%)	N (%)	N (%)
	Other ethnic group	N (%)	N (%)	N (%)
	Not stated	N (%)	N (%)	N (%)
	Missing			
**LARC selection if randomised to LARC (pilot phase recruits n=xx)**	LNG-IUS	N (%)	N (%)	N (%)
	DMPA	N (%)	N (%)	N (%)
	Either	N (%)	N (%)	N (%)
**LARC selection if randomised to LARC (main phase recruits n=xx)[1]**	LNG-IUS	N (%)	N (%)	N (%)
	DMPA	N (%)	N (%)	N (%)
	Randomly allocated	N (%)	N (%)	N (%)
**How was LARC selection[2] made (main phase recruits n=xx)[1]**	Patient's preference	N (%)	N (%)	N (%)
	Clinician advice	N (%)	N (%)	N (%)
	Neither	N (%)	N (%)	N (%)
**Previous treatments[2]**	LNG-IUS	N (%)	N (%)	N (%)
	DMPA	N (%)	N (%)	N (%)
	COCP	N (%)	N (%)	N (%)
	Missing			
**Surgical complications**	Yes	N (%)	N (%)	N (%)
	Missing			

[1] minimisation variable; [2] pre-randomisation selection; [3]Figures may total more than number randomised as treatments are not mutually exclusive.

Appendix E3: Description of initial administration of intervention(s)

Details of how LARCs were initially administered

	**LARC allocated**	
	**LNG-IUS (n (%))**	**DMPA (n (%))**
**N (Total=)**		
During surgery		NA
Before discharge		
Referred to GP/ sexual health clinic		
Failure to fit/administer		
Declined		
Missing		

Details of how COCP was initially prescribed

	**COCP [n (%)]**
**N (Total=)**	
First cycle of tablets given/prescription dispensed in hospital	
Referred to GP/ sexual health clinic	
Declined	
Missing	

Appendix E4: Adherence to allocated intervention

K-M plot: Time to first treatment change (see table for further details including definitions)

Appendix E5: Compliance to allocated intervention

Reasons for non-compliance by group (separate table to be reported for each group)

	On assigned trial treatment plus any other trial treatment[1] (N=)	On assigned trial treatment plus taking any other non-trial treatment[2] (N=)	Not on assigned trial treatment, taking any other trial treatment[3] (N=)	Not on assigned trial treatment, taking any other non-trial treatment[4] (N=)	Not taking any treatment (N=)
Lack of effectiveness	N (%)	N (%)	N (%)	N (%)	N (%)
Did not control my bleeding	N (%)	N (%)	N (%)	N (%)	N (%)
Irregular bleeding	N (%)	N (%)	N (%)	N (%)	N (%)
Prolonged bleeding	N (%)	N (%)	N (%)	N (%)	N (%)
Coil expulsion	N (%)	N (%)	N (%)	N (%)	N (%)
Pelvic infection	N (%)	N (%)	N (%)	N (%)	N (%)
Disliked treatment	N (%)	N (%)	N (%)	N (%)	N (%)
Tummy upset	N (%)	N (%)	N (%)	N (%)	N (%)
Disliked taking tablets	N (%)	N (%)	N (%)	N (%)	N (%)
Vomiting/diarrhoea	N (%)	N (%)	N (%)	N (%)	N (%)
Skin allergy	N (%)	N (%)	N (%)	N (%)	N (%)
Depression/mood swings	N (%)	N (%)	N (%)	N (%)	N (%)
Weight gain	N (%)	N (%)	N (%)	N (%)	N (%)
Thread problems	N (%)	N (%)	N (%)	N (%)	N (%)
Headaches/migraine	N (%)	N (%)	N (%)	N (%)	N (%)
Dizziness	N (%)	N (%)	N (%)	N (%)	N (%)
Hypertension/increased blood pressure	N (%)	N (%)	N (%)	N (%)	N (%)
Pelvic pain	N (%)	N (%)	N (%)	N (%)	N (%)
Other[5]	N (%)	N (%)	N (%)	N (%)	N (%)

[1] LNG-IUS (n=)/COCP (n=)/DMPA (n=)
[2] e.g. POP (n=)/mini-pill (n=)/Zoladex (n=)/Kliofem (n=)/Decapeptyl (n=)/prostap (n=)/implanon (n=)/nexplanon (n=)
[3] LNG-IUS (n=)/COCP (n=)/DMPA (n=)
[4] e.g. POP (n=)/mini-pill (n=)/Zoladex (n=)/Kliofem (n=)/Decapeptyl (n=)/prostap (n=)/implanon (n=)/nexplanon (n=)
[5] listed

Appendix E6: Protocol deviations

Protocol deviations by group

	LARC (N=)	COCP (N=)
Ineligible patients randomised	N (%)	N (%)
Other protocol deviations	N (%)	N (%)
…	N (%)	N (%)

Appendix E7: Primary outcome results

Primary outcome results – EHP-30[1] pain scale (core domain)

	**LARC** Mean (SD), n	**COCP** Mean (SD), n	Adjusted Mean Difference (95% CI)[2]	p-value
Baseline				
6 Months				
1 Year				
2 Years				
**3 Years[3]**				

[1] EHP-30 pain domain; score ranges from 0 (not affected) to 100 (worst affected)

[2] Difference>0 favour LARC.

[3] Primary outcome time-point

Longitudinal plot for EHP-30 pain scores at all time-points by group

<Insert>

Primary outcome (EHP-30[1] pain scale) sensitivity and supportive analyses (3 years follow-up)

	**LARC** Mean (SD), n	**COCP** Mean (SD), n	Adjusted Mean Difference (95% CI)[2]	p-value
Sensitivity analysis 1				
Sensitivity analysis 2				
Sensitivity analysis …				

[1] EHP-30 pain domain; score ranges from 0 (not affected) to 100 (worst affected)

[2] Difference>0 favour LARC.

Primary outcome (EHP-30[1] pain scale) subgroup analysis (3 years follow-up)

	**LARC** Mean (SD), n	**COCP** Mean (SD), n	Adjusted Mean Difference (95% CI)[2]	Interaction p-value
**Pre-randomisation selection of LNG-IUS or DMPA including all methods of allocation**				
LNG-IUS				
DMPA				
**Pre-randomisation selection of LNG-IUS or DMPA excluding random allocation**				
LNG-IUS				
DMPA				
**Pre-randomisation selection of LNG-IUS or DMPA including only those participants where the LARC was specifically chosen by the patient**				
LNG-IUS				
DMPA				
**Stage of Endometriosis**				
I/ II				
III/ IV				
**Extent of excision**				
Complete				
Incomplete				
**Age>35 years**				
Yes				
No				

[1] EHP-30 pain domain; score ranges from 0 (not affected) to 100 (worst affected)

[2] Difference>0 favour LARC.

Appendix E8: Secondary outcomes results

	**LARC** Mean (SD), n	**COCP** Mean (SD), n	Adjusted Mean Difference (95% CI)
**Endometriosis Health profile questionnaire (EHP-30)[1]**			
Core Domain: Control and Powerlessness			
Baseline			
6 months			
1 year			
2 years			
3 years			
Core Domain: Social support			
Baseline			
6 months			
1 year			
2 years			
3 years			
Core Domain: Emotional well-being			
Baseline			
6 months			
1 year			
2 years			
3 years			
Core Domain: Self-image			
Baseline			
6 months			
1 year			
2 years			
3 years			
Modular Domain: Work life			
Baseline			
6 months			
1 year			
2 years			
3 years			

	Modular Domain: Relationship with children				
	Baseline				
	6 months				
	1 year				
	2 years				
	3 years				
	Modular Domain: Sexual relationship				
	Baseline				
	6 months				
	1 year				
	2 years				
	3 years				
	Modular Domain: Feelings about medical profession				
	Baseline				
	6 months				
	1 year				
	2 years				
	3 years				
	Modular Domain: Feelings about treatment				
	Baseline				
	6 months				
	1 year				
	2 years				
	3 years				
	Modular Domain: Feelings about infertility				
	Baseline				
	6 months				
	1 year				
	2 years				
	3 years				
	**Visual Analogue Scale (VAS)[2]**				
	Pain during periods				
	Baseline				
	6 months				
	1 year				
	2 years				
	3 years				
	Pain during intercourse				
	Baseline				

6 months			
1 year			
2 years			
3 years			
Pain at any other time			
Baseline			
6 months			
1 year			
2 years			
3 years			
**Fatigue Severity Scale[3]**			
Summary score			
Baseline			
6 months			
1 years			
2 years			
3 years			
**Euroqol**			
EQ-5D-5L[4]			
Baseline			
6 months			
1 year			
2 years			
3 years			
Health thermometer[5]			
Baseline			
6 months			
1 year			
2 years			
3 years			
**ICE-CAP[6]**			
Capabilities			
Baseline			
6 months			
1 year			
2 years			
3 years			

[1] Scores range from 0 (best outcome) to 100 (worse outcome); scores<0 favour LARC

[2] Scores range from 0 (best outcome) to 10 (worse outcome); scores<0 favour LARC

[3] 7=best outcome, 63=worst outcome; scores<0 favour LARC
[4] EQ-5D-5L quality of life scores range from -0.59 (worse outcome) to 1.00 (best outcome); scores>0 favour UPA;
[5] Scores range from 0 (worse outcome) to 100 (best outcome); scores>0 favour LARC
[6] ICECAP-A scores range from 0 (worse outcome) to 1.0 (best outcome)

Secondary outcomes – are you still having periods?

	LARC n (%)	COCP n (%)	Adjusted Odds Ratio[1] (95%CI)
**Baseline**			
Yes			
No			
**TOTAL**			
**6 Months**			
Yes			
No			
**TOTAL**			
**1 year**			
Yes			
No			
**TOTAL**			
**2 years**			
Yes			
No			
**TOTAL**			
**3 years**			
Yes			
No			
**TOTAL**			

[1] Odds ratio; estimates<1 favour LARC.

Secondary outcomes – cycle regularity questions for those still having periods

	LARC n (%)	COCP n (%)	Adjusted Odds Ratio[1] (95%CI)
**Baseline**			
Regular			
Fairly regular			
Irregular			
Bleeding on and off			
**TOTAL**			
**6 Months**			
Regular			
Fairly regular			
Irregular			
Bleeding on and off			

**TOTAL**			
**1 year**			
Regular			
Fairly regular			
Irregular			
Bleeding on and off			
**TOTAL**			
**2 years**			
Regular			
Fairly regular			
Irregular			
Bleeding on and off			
**TOTAL**			
**3 years**			
Regular			
Fairly regular			
Irregular			
Bleeding on and off			
**TOTAL**			

[1] Odds ratio for 'regular' bleeding shown (regular + fairly regular); estimates>1 favour LARC.

Secondary outcomes – changes in pelvic pain

	**LARC** n (%)	**COCP** n (%)	Adjusted Odds Ratio[1] (95%CI)
**Baseline**			
Got much better			
Got a little better			
Not changed much			
Got worse			
**TOTAL**			
**6 Months**			
Got much better			
Got a little better			
Not changed much			
Got worse			
**TOTAL**			
**1 year**			
Got much better			
Got a little better			
Not changed much			
Got worse			
**TOTAL**			
**2 years**			
Got much better			

Got a little better			
Not changed much			
Got worse			
**TOTAL**			
**3 years**			
Got much better			
Got a little better			
Not changed much			
Got worse			
**TOTAL**			

[1] Odds ratio from proportional odds model shown; estimates<1 favour LARC.

Secondary outcomes – Further therapeutic surgery or second-line treatment for endometriosis over 3 years

	**LARC** n (%)	**COCP** n (%)
**Treatment failures**		
Hysterectomy		
Surgery for endometriosis		
Laparoscopy		
GnRH treatment		
**TOTAL**		
**Other surgeries**		
Removal of polyps		
Removal of fibroids		
Endometrial ablation		
Other types of surgery[1]		
**TOTAL**		

[1] Details

K-M plot: Time to further thereuptic surgery or second line treatment

K-M plot: Time to further thereuptic surgery or second line treatment (including those that returned to their pre-randomisation EHP-30 scores)

Pregnancy outcomes

	LARC<br>n (%)	COCP<br>n (%)
No. live births		
Normal		
Abnormal		
Stillbirth		
No. abortions		
Therapeutic		
Planned		
Spontaneous		
Mode of Delivery		
Normal		
Forceps/Ventouse		
Caesarean		

Appendix E9: Safety

Serious Adverse Events – overall numbers

	LARC (N=)	COCP (N=)	P-value
Total Number of Women Experiencing an SAE	N (%)	N (%)	
Total Number of SAE's	N (%)	N (%)	

Serious Adverse Events – further details by group

Summary of SAE	Reason for Reporting	Causality	Action taken
LARC			
1			
2			
3			
4			
COCP			
1			
2			
3			
4			

Appendix E10: data summaries from participants randomised to combinations that did not involve COCP

EHP-30[1] pain scores

	**LARC**<br>Mean (SD), n	**No treatment**<br>Mean (SD), n
Baseline		
6 Months		
1 Year		
2 Years		
3 Years		

[1] EHP-30 pain domain; score ranges from 0 (not affected) to 100 (worst affected)
