# Supplementary material for: Long acting progestogens versus combined oral contraceptive pill for preventing recurrence of endometriosis related pain: the PRE-EMPT pragmatic, parallel group, open label, randomised controlled trial
Source: BMJ. 2024 May 15;385:e079006. doi: 10.1136/bmj-2023-079006 (PMC11094611; doi:10.1136/bmj-2023-079006)
Supplement: Supplementary file 3 — Web appendix: Supplementary material 3—acknowledgments [file cook079006.ww3.pdf]

Supplementary Material 3 Acknowledgments

Members of the PRE-EMPT Collaborative Group

Chief Investigator – Kevin G Cooper, Aberdeen Royal Infirmary

Co- Investigators

Siladitya Bhattacharya, University of Aberdeen

Justin Clark, University of Birmingham

Jane Daniels, University of Nottingham

Lee Middleton, University of Birmingham

Versha Cheed, University of Birmingham

Tracy Roberts, University of Birmingham

Andrew Horne, University of Edinburgh

Janesh Gupta, University of Birmingham

Christian Becker, University of Oxford

Georgina Jones, Leeds Beckett University

Elaine Denny, Birmingham City University

Hilary Critchley, University of Edinburgh

Catherine Whittall, Robert Jones & Agnes Hunt Orthopaedic and District Hospital NHS Trust

Andrew Prentice, University of Cambridge

Ertan Saridogan, University College London Hospitals NHS Foundation Trust

Trial Management Group

Kevin G Cooper, Aberdeen Royal Infirmary

Siladitya Bhattacharya, University of Aberdeen

Jane Daniels, University of Nottingham

Jamie Godsall, University of Birmingham

Clive Stubbs, University of Birmingham

Danielle Pirrie, Aberdeen Royal Infirmary

Lee Middleton, University of Birmingham

Versha Cheed, University of Birmingham

Laura Ocansey, University of Birmingham

Lisa Leighton, University of Birmingham

Laura Gennard, University of Birmingham

Kirandeep Sunner, University of Birmingham

Mark Monahan, University of Birmingham

Konstantinos Tryposkiadis, University of Birmingham

Max Feltham, University of Birmingham

Rebecca Amos-Hirst, University of Birmingham

Leanne Fulcher, University of Birmingham

Additional Birmingham Clinical Trials Unit Staff

Annika Feilbach, BCTU Programmer

Adrian Wilcockson, BCTU Programmer

Recruiting site Principal Investigators and support staff

Aberdeen Royal Infirmary: Kevin Cooper (PI), Danielle Pirie, Minimol Paulose

Addenbrookes Hospital: Andrew Prentice (PI), Amy Sutton

Arrowe Park Hospital: Thomas Aust (PI), Julie Grindey

Bedford Hospital: Montasser Mahran (PI), Marina Laverdino

Birmingham Women's Hospital: Janesh Gupta (PI) Shanteela McCooty, Fiona Beale, Virginia Iqbal

Chesterfield Royal Hospital: Jennifer Parratt (PI) Louise Underwood, Mary Kelly Baxter

City Hospitals Sunderland: Nicholas Matthews (PI), Jane Scollen, Lesley Hewitt

Crosshouse Hospital: Santanu Acharya (PI), Cheryl Gibson, Debbie Callaghan

Doncaster Royal Infirmary: Manju Singh (PI), Jane Dumville

Forth Valley Royal Hospital: Shahzya Huda (PI), Anne Todd, Joanne Donnachie, Shoshana Morecroft

John Radcliffe Hospital: Christian Becker (PI), Fenella Roseman, Sarah Collins

Kings Mill Hospital: Jyothi Rajeswary (PI), Caro Moulds, Katie Slack, Rebecca Boulton

Leicester Royal Infirmary: Tarek Gelbaya (PI), Rupa Modi

Liverpool Women's Hospital: George Botros (PI), Elizabeth Kane, Gillian Smith, Kathie Cooke, Pamela Corlett

Milton Keynes General Hospital: Premila Thampi (PI), Cheryl Padilla, Edel Clare

Peterborough City Hospital: Bruce Ramsay (PI), Coralie Huson, Jodi Carpenter

Queens Medical Centre: Martin Powell (PI), Lucinda Wilson, Sophie Crowder

Royal Albert Edward Infirmary: Philip Harris (PI), Claire Fairhurst, Tracy Taylor

Royal Infirmary of Edinburgh: Andrew Horne (PI), Ann Doust, Helen Dewart

Royal Preston Hospital: Brice Rodriquez (PI), Anne Gardner

Royal Victoria Infirmary: Tony Chalhoub (PI), Alison Kimber

Southend Hospital: Sanjaya Kalkur (PI), Joanne Galliford, Prisca Gondo, Eunis Mshengu

St Marys Hospital: Kingshuk Majumder (PI), Christina Pritchard, Clare Waters, Lisa Cornwall, Louise Winter

St Richards Hospital: Bronwyn Middleton (PI), Emma Meadows, Sally Moore

Stepping Hill Hospital: Suku George (PI), Jayne Budd

Stoke Mandeville Hospital: Christopher Wayne (PI), Julie Tebbutt

The James Cook University Hospital: Pinky Khatri (PI), Mary Hodgers, Marrina Harrison, Helen Harwood, Hazel Alexander

University College Hospital: Dimitrios Mavrelos (PI), Sarah Ekladios

University Hospital of North Durham: Seema Sen (PI), Jean Dent, Vicki Atkinson

University Hospital of North Tees: Dolonchampa Basu (PI), Sharon Gowans, Wendy Cheadle

West Cumberland Hospital: Ajith Wijesiriwardana (PI), Rachel McCarthy, Toni Wilson, Una Poultney

Yeovil District Hospital: Ahmar Shah (PI), Dianne Wood, Kerry Rennie

York Hospital: Fawzia Sanaullah (PI), Deborah Phillips, Holly Alcock, Holly Hancock, Samantha Roche
