# Supplementary material for: Long acting progestogens versus combined oral contraceptive pill for preventing recurrence of endometriosis related pain: the PRE-EMPT pragmatic, parallel group, open label, randomised controlled trial
Source: BMJ. 2024 May 15;385:e079006. doi: 10.1136/bmj-2023-079006 (PMC11094611; doi:10.1136/bmj-2023-079006)

Supplementary Tables and Figures

Supplementary Table 1 Reasons for ineligibility

Reasons	N
**Eligible but declined consent**	**288**
**Declined due to treatment preference**	**326**
Preference for LNG-IUS	130
Preference for COCP	55
Had LNG-IUS and DMPA before, does not wish it again	38
Does not want any medical treatment	36
Preference for DMPA	25
Not willing to have any LAP	22
Does not like any of the treatment options	20
**Ineligible**	**1839**
Plans to conceive in the immediate future	355
Contraindications to the use of hormonal treatment with oestrogen or progestogens	221
No endometriosis identified at diagnostic laparoscopy	180
Deep infiltrating endometriosis requiring additional surgery	145
Age outside of range 16 – 46 years	121
Undergoing infertility treatment	111
History of drug sensitivity to COCP	89
Drug sensitivity to contraceptive pill	68
Patient did not attend	45
Other gynaecological treatment offered	28
Not able to understand written and spoken English.	12
Contradiction to DMPA	3
Any other reason	461
**TOTAL**	**2453**

Supplementary Table 2 Reasons for non-adherence (LAP group)

	Not on assigned treatment			On assigned treatment			
	Taking any other trial treatment (N=44)[1]	Taking any other non-trial treatment (N=5)[2]	Taking any other trial and non-trial treatment (N=10)[3]	Plus any other trial treatment (N=12)[4]	Plus any other non-trial treatment (N=4)[5]	Plus any other trial and non-trial treatment (N=1)[6]	Not taking any treatment (N=71)
Lack of effectiveness	7	1	4	3	0	1	17
Did not control my bleeding	4	1	3	2	0	0	13
Irregular bleeding	5	1	2	3	0	0	8
Prolonged bleeding	5	1	3	2	0	0	7
Coil expulsion	1	0	0	0	0	0	1
Pelvic infection	0	0	0	0	0	0	0
Disliked treatment	7	2	3	1	1	0	10
Tummy upset	3	1	3	1	0	0	3
Disliked taking tablets	0	1	0	0	0	0	0
Vomiting/ diarrhoea	2	1	0	0	0	0	1
Skin allergy	3	1	1	1	0	0	6
Depression/ mood swings	10	1	5	2	1	0	18
Weight gain	11	3	2	2	1	0	14
Thread problems	0	0	0	0	0	0	2
Headaches/	6	1	1	2	1	0	10

migraine							
Dizziness	2	1	0	2	0	0	7
Hypertension/ increased blood pressure	0	0	0	0	0	0	1
Pelvic pain	8	1	4	3	0	0	17
Trying to conceive	2	0	1	0	0	0	9
Pregnant	1	0	0	0	0	0	1
Other reasons	13	3	2	2	0	1	16

Numbers in brackets represent the total number of participant changes, multiple reasons can apply per participant. Treatment switches within the LAP group (i.e., between DMPA and LNG-IUS or vice-versa) are not considered to be cross-over in the first instance but are listed here as 'other trial treatments' for completeness.

[1] DMPA (n=31); LNG-IUS (n=11); COCP (n=2)

[2] Progesterone-only pill [POP] (n=1); other treatment or unclear (n=4)

[3] POP and COCP (n=10)

[4] LNG-IUS and DMPA (n=4); LNG-IUS and COCP (n=4); DMPA and COCP (n=2); LNG-IUS, DMPA and COCP (n=2);

[5] DMPA and other treatment or unclear (n=2); LNG-IUS and other treatment or unclear (n=2)

[6] LNG-IUS and other treatment or unclear (n=1)

Supplementary Table 3 Reasons for non-adherence (COCP group)

	Not on assigned treatment			On assigned treatment			
	Taking any other trial treatment (N=37)[1]	Taking any other non-trial treatment (N=8)[2]	Taking any other trial and non-trial treatment (N=1)[3]	Plus any other trial treatment (N=4)[4]	Plus taking any other non-trial treatment (N=8)[5]	Plus any other trial and non-trial treatment (N=0)	Not taking any treatment (N=75)
Lack of effectiveness	18	0	1	0	4	0	21
Did not control my bleeding	12	0	1	1	1	0	10
Irregular bleeding	13	0	1	1	1	0	7
Prolonged bleeding	8	0	1	1	2	0	11
Coil expulsion	2	0	0	0	0	0	0
Pelvic infection	2	0	0	0	0	0	2
Disliked treatment	2	0	0	0	1	0	10
Tummy upset	3	0	0	0	0	0	10
Disliked taking tablets	0	0	0	0	0	0	5
Vomiting/ diarrhoea	4	0	0	0	0	0	3
Skin allergy	2	0	0	0	1	0	1
Depression/mood swings	10	1	1	0	3	0	22
Weight gain	5	0	1	0	1	0	7
Thread problems	0	0	0	0	0	0	0

Headaches/ migraine	7	0	1	0	1	0	14
Dizziness	3	0	0	0	1	0	5
Hypertension/increased blood pressure	0	0	0	0	0	0	1
Pelvic pain	12	0	0	0	1	0	11
Trying to conceive	1	1	0	0	0	0	7
Pregnant	2	0	0	0	0	0	2
Other reasons	7	2	0	1	1	0	16

Numbers in brackets represent the total number of participant changes, multiple reasons can apply per participant.

[1] DMPA (n=19); LNG-IUS (n=18)

[2] POP (n=1); other implant progestogen (n=2); other treatment or unclear (n=5)

[3] LNG-IUS and POP (n=1)

[4] COCP and LNG-IUS (n=2); COCP and DMPA (n=2)

[5] COCP and POP (n=2); COCP and other treatment or unclear (n=6)

Supplementary Table 4 Results of EHP-30 pain scores at other time points[1]

	**LAP** Mean (SD), n	**COCP** Mean (SD), n	Adjusted Mean Difference (95% CI)[2]
Baseline	56.6 (17.3), 197	55.8 (19.9), 192	
6 Months	35.0 (25.6), 162	38.0 (26.4), 150	-1.9 (-7.0 to 3.2)
1 Year	35.1 (26.4), 150	37.5 (25.4), 153	-2.3 (-7.5 to 2.9)
2 Years	32.1 (26.2), 157	33.6 (26.5), 140	-0.4 (-5.6 to 4.9)

[1]EHP-30 pain domain; score ranges from 0 (not affected) to 100 (worst affected);

[2]Difference<0 favour LAP;

Supplementary Table 5 Primary outcome (EHP-30 pain scale) subgroup analysis (3 years follow-up)[1]

	LAP Mean (SD), n	COCP Mean (SD), n	Adjusted Mean Difference (95% CI)[2]	Interaction p-value
Pre-randomisation selection of LNG-IUS or DMPA including all methods of allocation				
LNG-IUS	32.1 (24.8), 71	37.2 (30.1), 67	-1.9 (-9.7 to 5.9)	0.95
DMPA	33.2 (24.9), 92	29.5 (25.4), 93	0.1 (-6.8 to 6.9)	
Pre-randomisation selection of LNG-IUS or DMPA excluding random allocation				
LNG-IUS	32.5 (25.0), 64	38.0 (30.4), 56	-1.7 (-9.9 to 6.5)	0.96
DMPA	34.3 (24.6), 83	29.5 (25.1), 87	1.3 (-5.6 to 8.3)	
Pre-randomisation selection of LNG-IUS or DMPA including only those participants where the LAP was specifically chosen by the patient				
LNG-IUS	31.3 (25.1), 46	35.9 (32.6), 37	-3.4 (-13.2 to 6.4)	0.84
DMPA	34.2 (22.1), 60	31.8 (25.0), 67	-1.5 (-9.6 to 6.5)	
Stage of Endometriosis				
I/ II	32.2 (24.6), 134	32.0 (26.5), 129	-1.6 (-7.3 to 4.0)	0.67
III/ IV	35.5 (26.4), 39	35.9 (31.4), 35	2.2 (-8.6 to 13.0)	
Extent of excision				
Complete	33.2 (24.5), 160	32.5 (27.6), 150	0.3 (-13.2 to 13.7)	0.94
Incomplete	29.5 (30.8), 13	36.9 (27.3), 14	-3.3 (-21.3 to 14.7)	
Age>=35 years				
Yes	25.1 (18.9), 36	24.6 (27.8), 35	3.6 (-7.1 to 14.4)	0.14
No	35.0 (26.0), 137	35.1 (27.2), 129	-2.0 (-7.6 to 3.6)	

[1]EHP-30 pain domain; score ranges from 0 (not affected) to 100 (worst affected); [2]Difference<0 favour LAP

Supplementary Table 6 Sensitivity analyses for primary outcome (EHP-30 pain scale at 3 years follow-up) investigating missing data assumptions

	Adjusted Mean Difference (95% CI)[2]
Multiple Imputation: Missing at random with delta value of 20% of average increase in both groups at each time point[1]	-1.0 (-6.0 to 4.0)
Multiple Imputation: Missing at random with delta value of 20% of average increase in the LAP group at each time point; 10% in the COCP treatment group[1]	-1.1 (-6.0 to 3.9)
Multiple Imputation: Missing at random with delta value of 10% of average increase in the LAP group at each time point; 20% in the COCP treatment group[1]	-0.9 (-5.9 to 4.1)
Analysis removing any late responses[1]	-2.2 (-7.4 to 3.0)

[1]EHP-30 pain domain; score ranges from 0 (not affected) to 100 (worst affected); [2]Difference<0 favour LAP

Supplementary Table 7 Results of secondary outcomes at other time points – other domains of the EHP-30[1]

	LAP<br>Mean (SD), n	COCP<br>Mean (SD), n	Adjusted Mean Difference (95% CI)
Core Domain: Control and Powerlessness			
Baseline	69.1 (19.7), 198	66.6 (23.4), 193	
6 months	46.1 (30.2), 160	49.3 (31.7), 148	-3.0 (-9.4 to 3.4)
1 year	47.9 (32.6), 150	48.7 (31.5), 150	-1.2 (-7.6 to 5.3)
2 years	42.1 (31.6), 127	43.1 (32.8), 110	-2.6 (-9.7 to 4.5)
Core Domain: Social support			
Baseline	56.8 (23.5), 198	56.5 (26.5), 193	
6 months	47.9 (31.2), 161	50.1 (33.6), 152	-0.9 (-7.3 to 5.4)
1 year	48.9 (31.9), 152	48.8 (31.5), 152	1.5 (-4.9 to 7.9)
2 years	43.8 (33.2), 127	46.8 (34.0), 111	-3.6 (-10.7 to 3.5)
Core Domain: Emotional well-being			
Baseline	53.0 (20.3), 198	52.4 (23.2), 193	
6 months	42.3 (27.2), 160	39.1 (27.2), 150	2.5 (-2.9 to 8.0)
1 year	42.2 (27.2), 152	40.1 (26.7), 151	1.9 (-3.5 to 7.4)
2 years	36.6 (27.4), 127	36.7 (29.6), 111	-0.9 (-6.9 to 5.1)
Core Domain: Self-image			
Baseline	54.3 (28.4), 198	52.6 (29.0), 194	
6 months	47.4 (33.2), 161	48.2 (33.4), 152	-1.0 (-7.6 to 5.7)
1 year	47.6 (33.8), 152	45.7 (33.2), 152	1.9 (-4.8 to 8.6)
2 years	40.6 (32.9), 127	43.0 (37.0), 111	-4.1 (-11.5 to 3.3)
Modular Domain: Work life			
Baseline	51.2 (25.9), 165	50.2 (28.0), 168	
6 months	29.9 (29.9), 136	32.9 (30.8), 126	-1.0 (-7.6 to 5.7)
1 year	33.8 (30.2), 126	29.5 (29.7), 121	6.0 (-0.8 to 12.9)

2 years	28.1 (29.1), 108	25.3 (29.3), 86	0.5 (-7.0 to 8.1)
Modular Domain: Relationship with children			
Baseline	40.5 (29.9), 107	33.5 (26.6), 87	
6 months	27.3 (28.1), 71	23.3 (26.7), 51	0.7 (-7.9 to 9.4)
1 year	27.4 (28.8), 68	26.8 (29.3), 55	-4.1 (-12.5 to 4.3)
2 years	20.5 (25.1), 53	22.2 (27.2), 40	-9.4 (-19.3 to 0.6)
Modular Domain: Sexual relationship			
Baseline	68.4 (26.0), 173	69.6 (24.3), 169	
6 months	56.9 (30.6), 138	53.9 (31.8), 130	2.5 (-4.4 to 9.5)
1 year	55.5 (33.8), 116	58.3 (31.3), 122	0.8 (-6.5 to 8.0)
2 years	52.6 (32.6), 104	54.1 (32.2), 92	1.9 (-6.0 to 9.8)
Modular Domain: Feelings about medical profession			
Baseline	36.0 (29.0), 169	31.2 (27.9), 162	
6 months	37.7 (30.4), 109	42.6 (30.5), 101	-5.7 (-14.4 to 3.0)
1 year	38.5 (33.0), 88	38.2 (31.7), 102	0.3 (-8.8 to 9.4)
2 years	37.6 (32.8), 83	37.0 (33.4), 67	2.4 (-7.7 to 12.5)
Modular Domain: Feelings about treatment			
Baseline	48.3 (26.1), 121	46.4 (27.5), 115	
6 months	44.5 (29.2), 137	44.7 (30.2), 130	-4.4 (-13.2 to 4.5)
1 year	51.6 (29.8), 102	44.9 (29.9), 124	6.5 (-2.9 to 15.9)
2 years	43.2 (31.6), 92	44.1 (32.8), 79	-0.3 (-10.9 to 10.2)
Modular Domain: Feelings about infertility			
Baseline	49.9 (32.5), 110	48.5 (33.7), 110	
6 months	58.5 (32.6), 75	48.7 (31.1), 70	11.7 (0.9 to 22.5)
1 year	58.1 (33.1), 66	44.3 (33.9), 68	9.1 (-2.2 to 20.4)
2 years	51.6 (35.7), 51	43.9 (33.2), 50	16.1 (2.7 to 29.5)

[1] EHP-30 Scores range from 0 (best possible health status) to 100 (worst possible health status); scores<0 favour LAP

Supplementary Table 8 Results of secondary outcomes at other time points – pelvic pain

Using Visual Analogue Scale (VAS)[1]			
Time point	LAP<br>Mean (SD), n	COCP<br>Mean (SD), n	Adjusted Mean Difference (95% CI)
Pain during periods			
Baseline	7.8 (1.4), 158	7.9 (1.5), 152	
6 months	6.5 (2.6), 80	6.8 (2.3), 110	-0.2 (-0.8 to 0.4)
1 year	6.8 (2.3), 76	6.9 (1.9), 106	0.2 (-0.4 to 0.9)
2 years	6.4 (2.2), 61	6.5 (2.2), 64	-0.1 (-0.8 to 0.7)
Pain during intercourse			
Baseline	6.4 (2.4), 150	6.4 (2.6), 159	
6 months	5.1 (2.8), 119	4.9 (2.6), 109	0.0 (-0.7 to 0.7)
1 year	5.4 (2.8), 104	5.7 (2.5), 103	0.3 (-0.4 to 1.0)
2 years	5.3 (2.8), 82	5.0 (2.7), 75	-0.2 (-1.0 to 0.6)
Pain at any other time			
Baseline	6.4 (2.0), 180	5.8 (2.1), 175	
6 months	5.1 (2.7), 140	4.9 (2.7), 134	0.0 (-0.5 to 0.6)
1 year	5.5 (2.5), 131	5.2 (2.4), 134	0.0 (-0.5 to 0.6)
2 years	5.1 (2.4), 107	5.2 (2.5), 95	0.2 (-0.4 to 0.8)
Using Likert scale[2]			
**Time point**	**LAP**<br>**N (%)**	**COCP**<br>**N (%)**	**Adjusted Odds Ratio[3] (95%CI)**
**6 Months**			
Got much better	27 (18%)	19 (14%)	1.56 (0.97 to 2.51)
Got a little better	24 (16%)	22 (16%)	
Not changed much	71 (49%)	64 (46%)	
Got worse	24 (16%)	35 (25%)	

TOTAL	N=146	N=140	
1 year			
Got much better	14 (10%)	8 (6%)	
Got a little better	18 (13%)	15 (11%)	1.02 (0.64 to 1.64)
Not changed much	57 (41%)	74 (54%)	
Got worse	49 (36%)	39 (29%)	
TOTAL	N=138	N=136	
2 years			
Got much better	8 (7%)	11 (11%)	
Got a little better	10 (9%)	8 (8%)	0.83 (0.48 to 1.43)
Not changed much	56 (51%)	48 (50%)	
Got worse	35 (32%)	29 (30%)	
TOTAL	N=109	N=96	

[1]VAS Scores range from 0 (best outcome) to 10 (worse outcome); scores<0 favour LAP

[2]Baseline data included for those returned a form at either 6-month, 1 year, 2 years or 3 years.

[3]Odds ratio from proportional odds model shown; estimates<1 favour LAP.

Supplementary Table 9 Results of secondary outcomes at other time points – Fatigue Severity Score (FSS)[1]

	LAP<br>Mean (SD), n	COCP<br>Mean (SD), n	Adjusted Mean Difference (95% CI)
Baseline	43.6 (14.1), 197	42.3 (13.4), 191	
6 months	41.9 (15.0), 160	40.6 (15.6), 150	1.5 (-1.6 to 4.5)
1 years	44.0 (15.7), 151	40.7 (15.2), 152	3.2 (0.2 to 6.3)
2 years	43.4 (13.9), 125	41.4 (16.4), 109	1.7 (-1.7 to 5.0)

[1]Fatigue Severity Scale scores range from 9 to 63 (Higher the score=greater fatigue severity); scores<0 favour LAP

Supplementary Table 10 Results of secondary outcomes at other time points – EQ-5D-5L[1]

	LAP<br>Mean (SD), n	COCP<br>Mean (SD), n	Adjusted Mean Difference (95% CI)
Baseline	0.63 (0.24), 198	0.63 (0.24), 190	
6 months	0.68 (0.24), 160	0.67 (0.28), 149	-0.01 (-0.06 to 0.04)
1 year	0.67 (0.28), 151	0.67 (0.25), 152	0.01 (-0.04 to 0.07)
2 years	0.67 (0.28), 157	0.69 (0.27), 141	0.01 (-0.05 to 0.06)

[1]EQ-5D-5L scores ranges from-0.59 (worse outcome) to 1.00 (best outcome); scores>0 favour LAP;

Supplementary Table 11 Results of secondary outcomes at other time points – still experiencing periods (menstrual status)

	LAP<br>N (%)	COCP<br>N (%)
6 Months		
Yes	87 (54%)	116 (76%)
No	74 (46%)	36 (24%)
TOTAL	N=161	N=152
1 year		
Yes	81 (54%)	108 (70%)
No	70 (46%)	46 (30%)
TOTAL	N=151	N=154
2 years		
Yes	54 (43%)	72 (65%)
No	72 (57%)	38 (35%)
TOTAL	N=126	N=110

Supplementary Table 12 Results of secondary outcomes at other time points - menstrual cycle regularity

	LAP N (%)	COCP N (%)	Adjusted Odds Ratio[1] (95%CI)
**Baseline**			
Regular	23 (20%)	23 (18%)	
Fairly regular	37 (32%)	36 (28%)	
Irregular	34 (29%)	39 (31%)	
Bleeding on and off	23 (20%)	29 (23%)	
**TOTAL**	**N=117**	**N=127**	
**6 Months**			
Regular	6 (7%)	26 (23%)	
Fairly regular	23 (26%)	39 (34%)	
Irregular	35 (40%)	31 (27%)	0.32 (0.16 to 0.63)[2]
Bleeding on and off	23 (23%)	18 (16%)	
**TOTAL**	**N=87**	**N=114**	
**1 year**			
Regular	12 (15%)	25 (23%)	
Fairly regular	27 (34%)	39 (36%)	
Irregular	28 (35%)	29 (27%)	0.73 (0.39 to 1.39)[2]
Bleeding on and off	13 (16%)	14 (13%)	
**TOTAL**	**N=80**	**N=107**	
**2 years**			
Regular	9 (17%)	17 (24%)	
Fairly regular	22 (42%)	27 (38%)	
Irregular	19 (36%)	18 (25%)	0.94 (0.39 to 2.23)[2]
Bleeding on and off	3 (6%)	9 (13%)	
**TOTAL**	**N=53**	**N=71**	

Baseline data included for those returned a form at either 6 months, 1 year, 2 years or 3 years. [1]Odds ratio for 'regular' bleeding shown (regular + fairly regular); estimates>1 favour LAP;

Supplementary Table 13 Pregnancy details

	LAP<br>N	COCP<br>N
**No. of pregnancies**	**17**	**24**
**No. of deliveries**	**12**	**18**
Normal	12	18
Abnormal	0	0
Stillbirth	0	0
**Mode of Delivery**	**12**	**18**
Normal	5	11
Forceps/Ventouse	2	3
Caesarean	5	4
**No. abortions/miscarriages**	**4**	**5**
Abortion: Therapeutic	1	0
Abortion: Planned	2	2
Miscarriage	1	3

Supplementary Table 14 Details of further surgical procedures or second-line medical treatment for endometriosis over 3 years

	LAP<br>N	COCP<br>N
Hysterectomy	6	14
Surgery for endometriosis	21	30
Laparoscopy	22	28
Medical (GnRHa) treatment	24	25
**TOTAL[1]**	**73**	**97**
**Total number of women experiencing treatment failure**	**50**	**61**

[1]Women may have had more than one surgical procedure or also had medical treatment

Supplementary Figure 1 Time to first treatment change - Panel A: no longer on assigned treatment Panel B: Any treatment change

Panel A

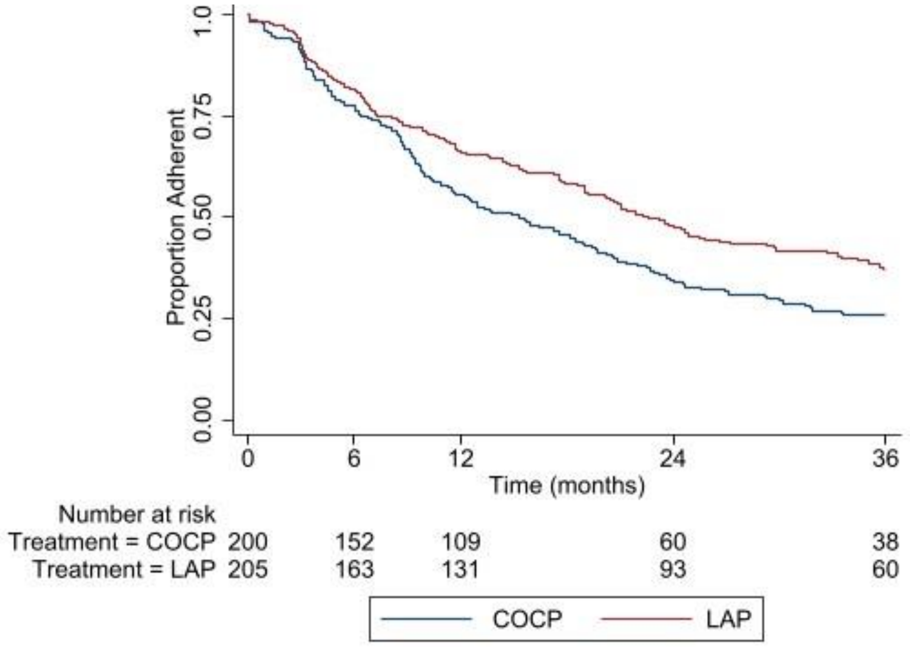

Note: changes from one LAP to another (e.g., LNG-IUS to DMPA) are not classified as a change

Panel B

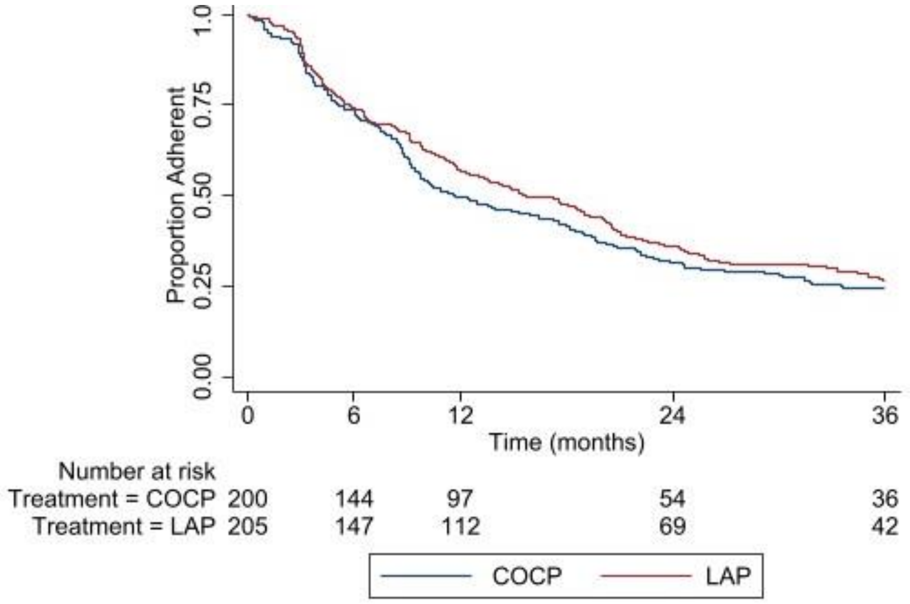

Note: includes any relevant treatment change, i.e., includes the addition of a trial or related non-trial treatment

Supplementary Figure 2 Time to first treatment change (LAP group split by intervention used) - Panel A: no longer on assigned treatment Panel B: Any treatment change

Panel A

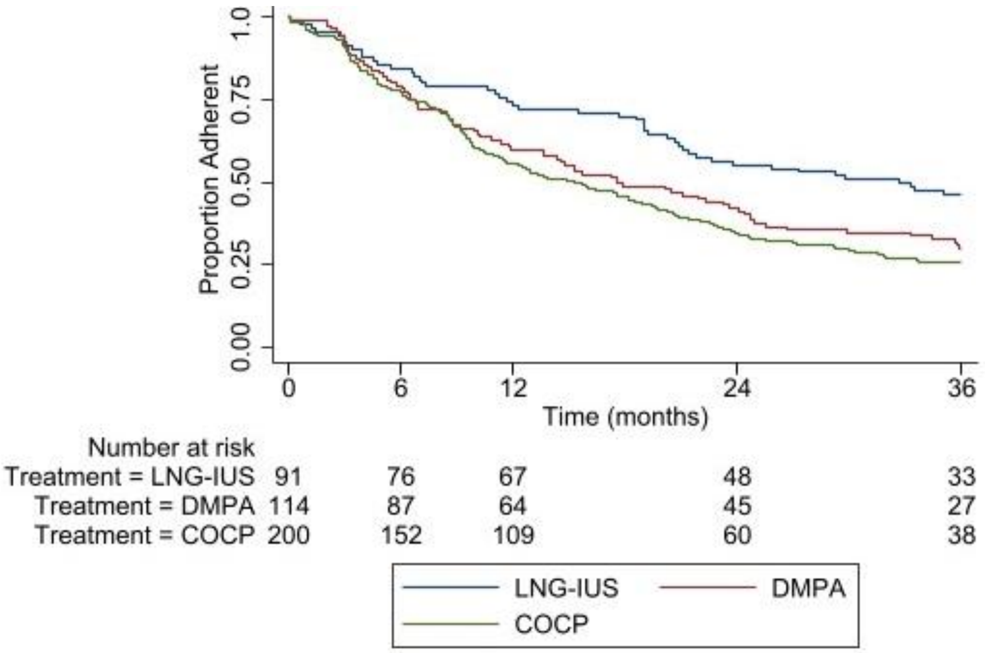

Panel B

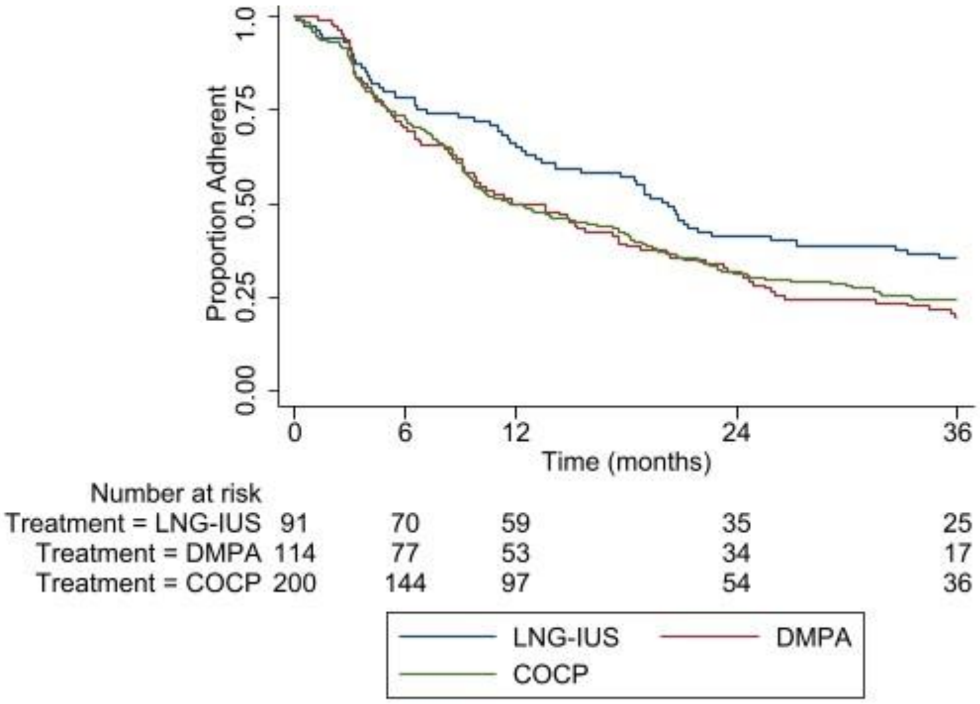

Note: includes any relevant treatment change, i.e., includes the addition of a trial or related non-trial treatment

Supplementary Figure 3 Kaplan-Meier plot of time to further therapeutic surgery or second-line treatment or EHP-30 score less than pre-randomisation score

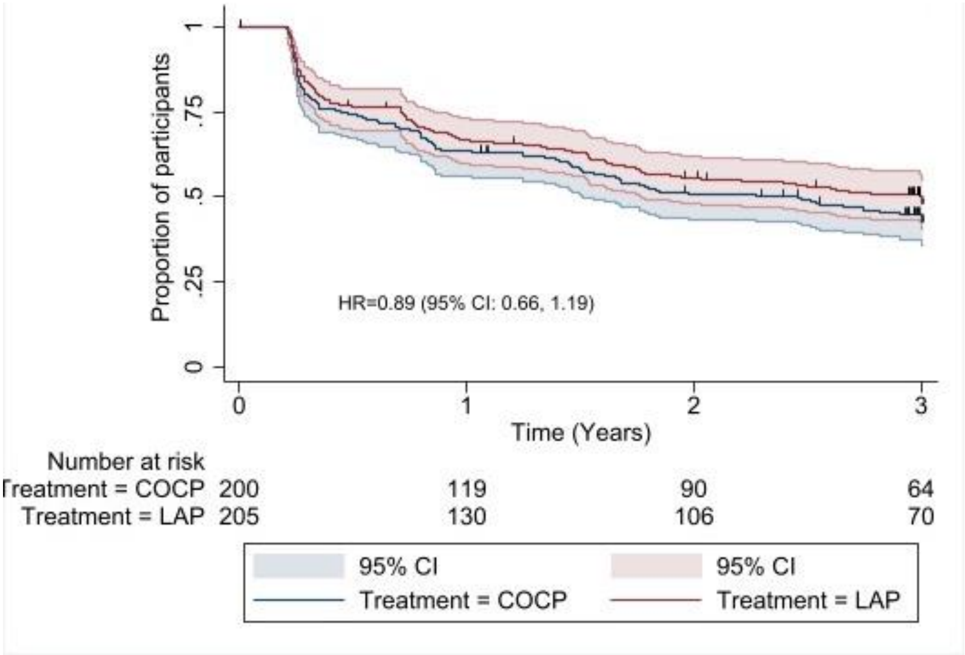

Supplement: Supplementary file 4 — Web appendix: Supplementary material 4—tables and figures [file cook079006.ww4.pdf]
